# Enantioselective Cytotoxicity of Chiral Diphosphine Ruthenium(II) Complexes Against Cancer Cells

**DOI:** 10.1002/chem.202200200

**Published:** 2022-05-05

**Authors:** Denise Lovison, Dario Alessi, Lorenzo Allegri, Federica Baldan, Maurizio Ballico, Giuseppe Damante, Marilisa Galasso, Daniele Guardavaccaro, Silvia Ruggieri, Andrea Melchior, Daniele Veclani, Chiara Nardon, Walter Baratta

**Affiliations:** ^1^ Dipartimento di Scienze Agroalimentari, Ambientali e Animali Università di Udine Via Cotonificio 108 33100 Udine Italy; ^2^ Dipartimento di Area Medica - Istituto di Genetica Medica Università di Udine Via Chiusaforte, F3 33100 Udine Italy; ^3^ Centro di Ricerca LURM Laboratorio Interdipartimentale di Ricerca Medica Università di Verona, Policlinico G.B. Rossi P.L.A. Scuro 10 37134 Verona Italy; ^4^ Dipartimento di Biotecnologie Università di Verona Strada Le Grazie, 15 37134 Verona Italy; ^5^ Dipartimento Politecnico di Ingegneria e Architettura Università di Udine Via Cotonificio 108 33100 Udine Italy

**Keywords:** antitumor agents, chirality, cytotoxicity, N Ligands, P Ligands, ruthenium

## Abstract

The chiral cationic complex [Ru(η^1^‐OAc)(CO)((*R,R*)‐Skewphos)(phen)]OAc (**2** ^***R***^), isolated from reaction of [Ru(η^1^‐OAc)(η^2^‐OAc)(*R,R*)‐Skewphos)(CO)] (**1** ^***R***^) with phen, reacts with NaOPiv and KSAc affording [RuX(CO)((*R,R*)‐Skewphos)(phen)]Y (X=Y=OPiv **3** ^***R***^; X=SAc, Y=OAc **4** ^***R***^). The corresponding enantiomers **2** ^***S***^‐**4** ^***S***^ have been obtained from **1** ^***S***^ containing (*S,S*)‐Skewphos. Reaction of **2** ^***R***^ and **2** ^***S***^ with (*S*)‐cysteine and NaPF_6_ at pH=9 gives the diastereoisomers [Ru((*S*)‐Cys)(CO)(PP)(phen)]PF_6_ (PP=(*R,R*)‐Skewphos **2** ^***R***^‐Cys; (*S,S*)‐Skewphos **2** ^***S***^‐Cys). The DFT energetic profile for **2** ^***R***^ with (*S*)‐cysteine in H_2_O indicates that aquo and hydroxo species are involved in formation of **2** ^***R***^‐Cys. The stability of the ruthenium complexes in 0.9 % w/v NaCl solution, PBS and complete DMEM medium, as well as their *n*‐octanol/water partition coefficient (logP), have been evaluated. The chiral complexes show high cytotoxic activity against SW1736, 8505 C, HCT‐116 and A549 cell lines with EC_50_ values of 2.8–0.04 *μ*M. The (*R*,*R*)‐Skewphos derivatives show higher cytotoxicity compared to their enantiomers, **4** ^***R***^ (EC_50_=0.04 *μ*M) being 14 times more cytotoxic than **4** ^***S***^ against the anaplastic thyroid cancer 8505 C cell line.

## Graphical abstract

**The chiral cationic** diphosphine ruthenium(II) complexes [RuX(CO)(PP)(phen)]Y (X, Y=carboxylates, thioacetate, PP=(*R,R*)‐ or (*S*,*S*)‐Skewphos), isolated as single stereoisomers, inhibit cancer cell proliferation and colonization with EC_50_ values ranging from 2.8 to 0.04 *μ*M, the (*R*,*R*)‐enantiomers being up to 14 times more active than the (*S*,S) ones. In water, the (*S*)‐cysteine and GSH adducts are easily formed from the carboxylate derivatives via aquo complexes.

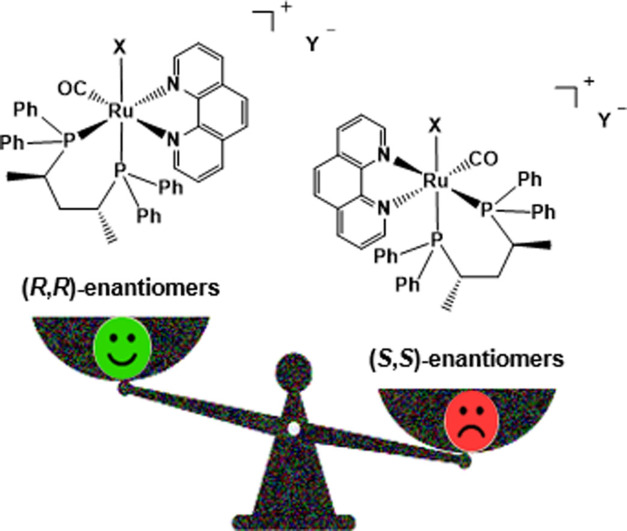

## Introduction

**Figure 1:**
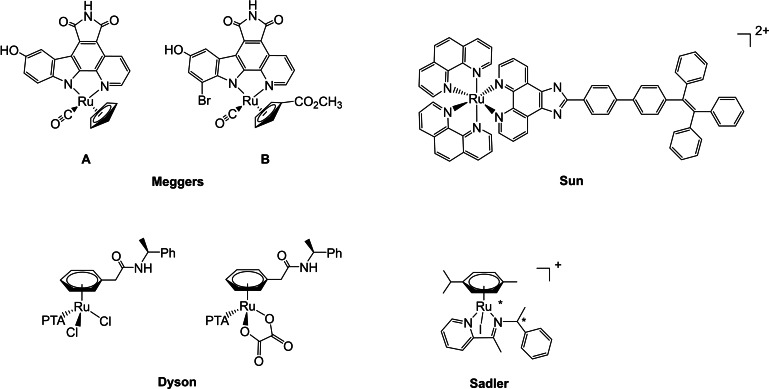
Biological active chiral ruthenium complexes.

The control of the configuration at the metal center is a key issue for the fine‐tuning of the properties of transition metal complexes, which can find applications in catalysis and pharmacology. As a matter of fact, the use of suitable chiral ligands has been demonstrated a valuable strategy for achieving highly stereoselective catalytic reactions. Recently, great effort has been devoted to the search of efficient chiral anticancer complexes, a well‐known example is oxaliplatin, bearing (*R*,*R*)‐cyclohexane‐1,2‐diamine, which forms DNA adducts with a higher rate than its (*S*,*S*)‐enantiomer.[^1^, ^2^, ^3^] Although ruthenium derivatives are considered promising candidates as chemotherapeutic agents in addition to the platinum ones,[^4^, ^5^] only few examples of chiral ruthenium complexes have been investigated.^[6]^ In 2006 Meggers and co‐workers isolated the derivatives **A** and **B** (Figure 1), containing a bidentate staurosporine, which are inhibitors of the glycogen synthase kinase‐3 (GSK‐3) with 6 and 260‐fold higher activity, compared to their enantiomers.[^7^, ^8^]

The polypyridyl Λ‐Ru(phen)_2_(TPEPIP)]^2+^ derivative, described by Sun et al., can induce apoptosis of tumor cells showing an IC_50_ as low as 7.6 μM, a value 3‐times lower than the Δ‐Ru enantiomer (Figure 1).^[9]^ Interestingly, Dyson and co‐workers developed RAPTA derivatives containing a chiral acetamide functionalized arene, the oxalate (*S*)‐enantiomer being almost 4‐fold more cytotoxic with an IC_50_ of 8.7 μM against the A2780 cancer cell line, than the analogous (*R*)‐enantiomer.^[10]^ Sadler's group investigated the arene ruthenium [RuI(η^6^‐arene)(NN)]^+^ complexes with chiral iminopyridines, affording four stereoisomers of poor anticancer activity, with a less than 2‐fold difference in cytotoxicity among the species (Figure 1).^[11]^ Notably, the related osmium complexes [OsX(η^6^‐arene)(NN)]^+^ (X=Cl, I) showed potent anticancer activity and similarities for the pair of enantiomers with IC_50_ as low as 0.6 μM.[^12^, ^13^] Recently, chiral ruthenium complexes with phosphines,[^14^, ^15^] diiminopyridine ligands^[16]^ and sulfur amino acids,^[17]^ have been reported to display cytotoxic activity against cancer cells.

Chiral ruthenium complexes have been deeply investigated in the asymmetric hydrogenation^[18]^ and transfer hydrogenation[^19^, ^20^] of carbonyl compounds. Outstanding catalysts are the arene [RuCl(η^6^‐arene)(TsDPEN)],[^21^, ^22^, ^23^] the diphosphine [RuCl_2_(PP)(NN)],[^24^, ^25^, ^26^, ^27^] as well as the carboxylate [Ru(η^1^‐OCOR)_2_(PP)(en)]^[28]^ and [Ru(η^2^‐OAc)(CO)(PP)(NN)]OAc[^29^, ^30^] (PP=diphosphine, NN=diamine, ampy) complexes.

Recently, we described that the cationic complexes [RuX(CO)(dppb)(phen)]Y (X, Y=Cl, carboxylate)[^21^, ^31^] display high cytotoxic activity against anaplastic thyroid cancer (ATC)^[32]^ cell lines with EC_50_ values much lower than that of Cisplatin, with an increment of apoptosis and reduction of cancer cell aggressiveness. It is worth pointing out that the arene ruthenium complexes which have been investigated as promising anticancer drugs[^33^, ^34^, ^35^] can be involved in the disruption of the cellular redox homeostasis via NADH transfer hydrogenation, as well as GSH metal thiol binding and oxidation.^[36]^ Conversely, only few studies have been reported on the use of diphosphine ruthenium hydrogenation catalysts as efficient anticancer systems.[^37^, ^38^, ^39^, ^40^]

Herein we report the isolation of the cationic enantiomer complexes [RuX(CO)(PP)(phen)]Y (X, Y=carboxylates, thioacetate, PP=(*R,R*)‐ or (*S*,*S*)‐Skewphos)^[21]^ and their behaviour with (*S*)‐cysteine and GSH via formation of aquo complexes. Remarkably different and very promising cytotoxic activity toward several cell lines has been observed for the couples of enantiomers.

## Results and Discussion

### Synthesis and characterization of cationic chiral ruthenium complexes

Treatment of the acetate complex [Ru(η^1^‐OAc)(η^2^‐OAc)((*R,R*)‐Skewphos)(CO)]^[30]^ (**1** ^***R***^) with phen (1 equiv) in methanol at 60 °C overnight affords the cationic derivative [Ru(η^1^‐OAc)(CO)((*R,R*)‐Skewphos)(phen)]OAc (**2** ^***R***^) in 91 % yield, as a single stereoisomer (Eq. (1)). 
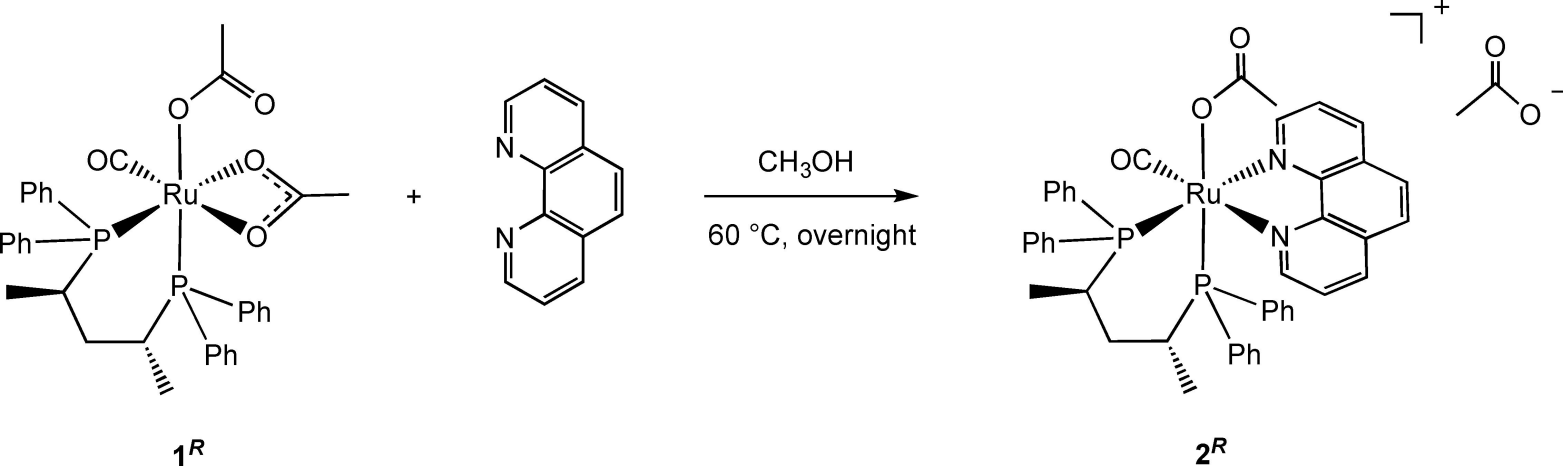


**Figure 2:**
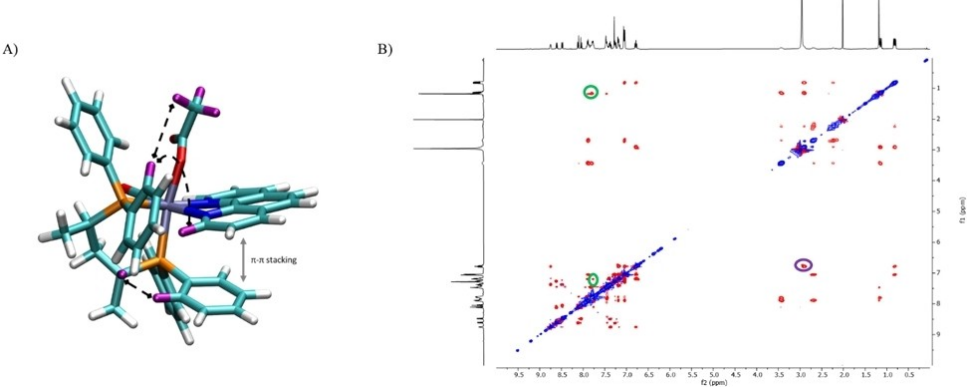
A) Most relevant NOE effects of **2** ^***R***^ displayed by black dashed lines. Involved protons are highlighted in purple; B) NOESY ^1^H‐^1^H NMR spectrum of **2** ^***R***^ in CDCl_3_. Circled in green: NOE effects of the phenyl protons at *δ* 7.78 ppm with both the acetate methyl group at *δ* 1.19 ppm and the phenanthroline *ortho* proton at *δ* 7.18; Circled in purple: NOE effects between the chiral CH proton at *δ* 2.91 ppm and the two phenyl *ortho* protons at *δ* 6.79 ppm.

The ^31^P{^1^H} NMR spectrum in CDCl_3_ of the thermally stable complex **2** ^***R***^ displays two doublets at *δ* 42.9 and 41.0 ppm (^2^ *J*_PP_=32.2 Hz) for the P atoms *trans* to N and acetate O atoms, respectively, as established by a ^31^P‐^1^H HMBC measurement, showing a long‐range coupling between the P atom at *δ*_P_ 42.9 ppm with the *ortho* phenanthroline proton at *δ*_H_ 8.75 ppm, which points toward the CO ligand. The ^1^H NMR signals at *δ* 3.43 and 1.16 ppm are for the PCH‐CH_3_ moiety of the P *trans* to N atom, while the other PCH‐CH_3_ resonances for the P *trans* to the acetate are at δ 2.91 and 0.82 ppm (Figures S1–S3). NMR measurements show that the two *ortho* protons at *δ* 7.78 ppm of the phenyl bound to the P *trans* to N show NOE effect with both the acetate methyl group (*δ* 1.19 ppm) and with the *ortho* phenanthroline proton at *δ* 7.18. In addition, the chiral CH proton at *δ* 2.91 ppm exhibits a NOE effect with the two up‐field *ortho* protons at *δ* 6.79 ppm of the phenyl bound to the P *trans* to OAc, consistent with the assigned configuration of the ruthenium center (Figure 2).

**Scheme 1:**
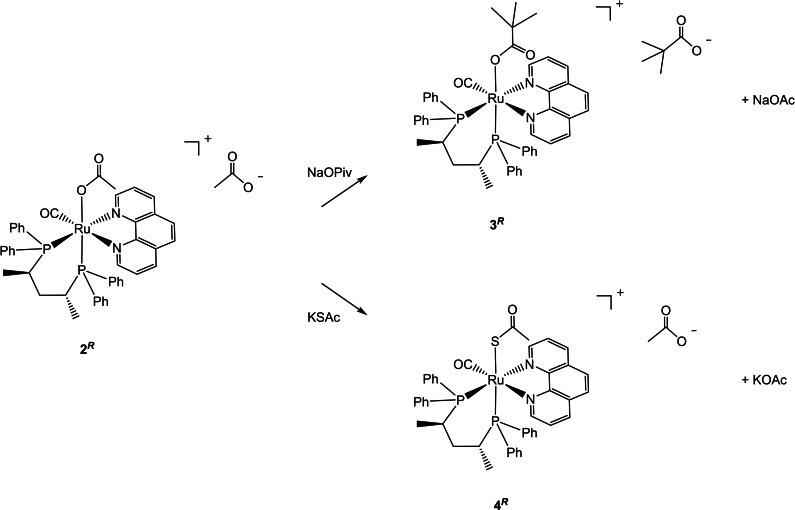
Synthesis of the complexes **3** ^***R***^ and **4** ^***R***^ from **2** ^***R***^ in MeOH at 60 °C.

The pivalate derivative [Ru(η^1^‐OPiv)(CO)((*R,R*)‐Skewphos)(phen)]OPiv (**3** ^***R***^) was easily prepared in high yield, by treatment of **2** ^***R***^ with NaOPiv (10 equiv) in methanol at 60 °C for 24 h via displacement of OAc (Scheme 1).PUT SCHEME 1 HERE

**Figure 3:**
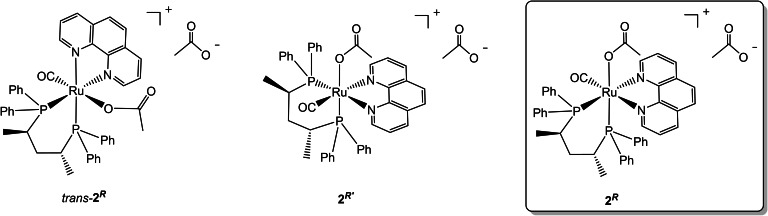
Possible *trans* and *cis*‐Ru(η^1^‐OAc)(CO)((*R,R*)‐Skewphos)(phen)]OAc stereoisomers.

The up‐field shift of two *ortho* Ph hydrogens is due to the superimposition of one phenyl with the phenanthroline ring through π‐π‐interactions, as observed in related ruthenium complexes containing pyridine ligands *cis* to the PPh_2_ moiety.^[41]^ Interestingly, no formation of the other possible stereoisomers, namely *trans*‐**2** ^***R***^ and the additional *cis* **2** ^***R’***^ complexes, has been observed upon heating, suggesting that *cis* **2** ^***R***^ is the thermodynamically most stable species in agreement with the DFT calculations (see further part) and our previous studies on the *trans*‐*cis* isomerization of phosphine‐pyridine ruthenium complexes (Figure 3).^[26]^ The ^31^P{^1^H} NMR spectrum of **3** ^***R***^ in CDCl_3_ shows two doublets at *δ* 43.7 and 41.6 ppm (^2^ *J*_PP_=31.4 Hz), whereas the ^1^H NMR singlets at *δ* 1.22 and 0.05 ppm correspond to the methyl groups of the free and coordinated pivalate, respectively. In the ^13^C{^1^H} NMR spectra, the CO carbon appears as a doublet of doublets at *δ* 204.7 ppm (^2^ *J*_CP_=20.0 and 15.2 Hz), while the free and coordinated pivalate carbonyl moieties appear as singlets at *δ* 184.0 and 183.6 ppm, respectively. Similarly, the thioacetate [Ru(η^1^‐SAc)(CO)((*R,R*)‐Skewphos)(phen)]OAc **4** ^***R***^ was obtained by treatment of **2** ^***R***^ with KSAc (10 equiv) in methanol at 60 °C overnight, by displacement of the coordinated OAc (Scheme 1). The ^31^P{^1^H} NMR spectrum of **4** ^***R***^ in CDCl_3_, exhibits a doublet at *δ* 41.0 ppm (^2^ *J*_PP_=29.6 Hz) for the P *trans* to the N atom and an up‐field shielded doublet at *δ* 31.0 ppm for the P *trans* to S atom. Complex **4** ^***R***^ has been isolated with acetate as counterion, as inferred from ^13^C{^1^H} NMR measurements showing two CO singlets at δ 204.1 and 176.1 ppm for the coordinated SAc and free OAc moieties, in addition to the doublets of doublets at δ 205.5 ppm (^2^ *J*_CP_=19.5 and 12.2 Hz) for the Ru‐CO.

**Scheme 2:**
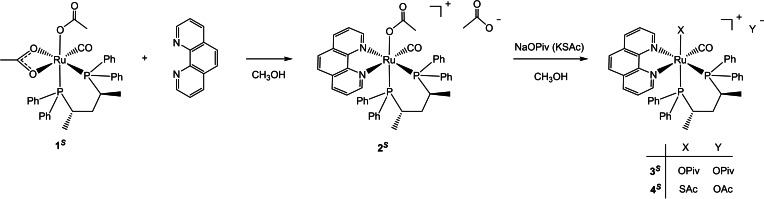
Synthesis of the enantiomers **2** ^***S***^, **3** ^***S***^ and **4** ^***S***^.

The use of the precursor [Ru(η^1^‐OAc)(η^2^‐OAc)((*S,S*)‐Skewphos)(CO)] (**1** ^***S***^), in place of the enantiomer **1** ^***R***^, with phen and following the same procedures described above, leads to the acetate [Ru(η^1^‐OAc)(CO)((*S,S*)‐Skewphos)(phen)]OAc (**2** ^***S***^) in 87 % yield (Scheme 2).

The derivative **2** ^***S***^ reacts with NaOPiv and KSAc in methanol, affording the pivalate [Ru(η^1^‐OAc)(CO)((*S,S*)‐Skewphos)(phen)]OAc (**3** ^***S***^) and thioacetate [Ru(η^1^‐SAc)(CO)((*S,S*)‐Skewphos)(phen)]OAc (**4** ^***S***^) isolated in 78 and 75 % yield, respectively.

### Reactivity of the chiral Skewphos complexes

**Scheme 3:**
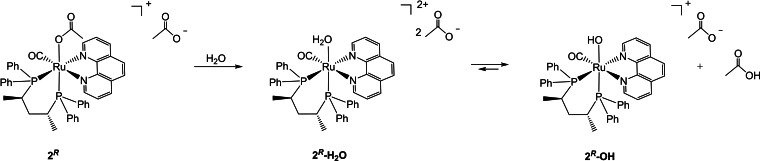
Formation of the aquo and hydroxo complexes from **2** ^***R***^ in water.

The cationic complexes [RuX(CO)(Skewphos)(phen)]Y (X, Y=OAc, OPiv, SAc) are highly soluble and stable in alcohols (MeOH, EtOH), acetone and DMSO under inert atmosphere, while in CH_2_Cl_2_ they slowly decompose (hours) by reaction of the counter ion. NMR studies carried out in CD_3_OD at 60 °C revealed that **2** ^***R***^ promptly reacts with KSAc, affording the thioacetate derivative **4** ^***R***^ by displacement of the acetate ligand, while **4** ^***R***^ does not react with NaOAc or NaOPiv, indicating a stronger Ru−S vs. R−O bond, in line with the previous investigations on related complexes.^[31]^ By difference to **4** ^***R***^, the derivatives **2** ^***R***^ and **3** ^***R***^ are soluble in water, resulting in carboxylate displacement and formation of hydroxo species (Supporting Information, Figure S4). Thus, NMR measurements show that complex **2** ^***R***^ (3 mM) in D_2_O at 37 °C leads to the formation of the hydroxo species [Ru(OH)(CO)((*R,R*)‐Skewphos)(phen)]OAc (**2** ^***R***^‐**OH**) (δ_P_=44.7 and 38.9 ppm, ^*2*^ *J_PP_* =35.5 Hz) in the presence of **2** ^***R***^ (**2** ^***R***^‐**OH**/**2** ^***R***^ ***=*** 1/2 molar ratio). The derivative **2** ^***R***^ in H_2_O provides a pH of about 4.5, which is a value close to that of a buffer solution of acetic acid‐acetate (pK_a_ of acetic acid=4.76 at 25 °C) consistent with the deprotonation of the dicationic aquo complex **2** ^***R***^‐**H_2_O**, affording the hydroxo **2** ^***R***^‐**OH** as the main species (Scheme 3).

As a matter of fact, thermodynamic studies demonstrate that hydrate carboxylates lead to the formation of strongly stabilized RCOO^−^ HOOCR species.^[42]^ Similarly, the ^31^P{^1^H} NMR spectrum of pivalate **3** ^***R***^ in D_2_O shows the signals of **3** ^***R***^‐**OH** in addition to those of **3** ^***R***^. Conversely, the thioacetate complex **4** ^***R***^, which shows poor water solubility and can be dissolved by addition of DMSO‐*d*_6_ (10 % v/v), shows no displacement of MeCOS^−^ with H_2_O, in agreement with our previous studies on [RuX(CO)(dppb)(phen)]Y.^[31]^

**Scheme 4:**
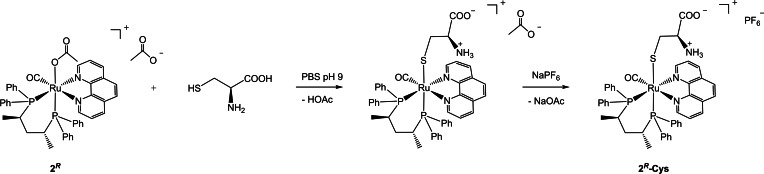
Reaction of **2** ^***R***^ with (*S*)‐cysteine in water.

Transition metal complexes are susceptible to interact with biological nucleophiles (i. e. nucleobases, glutathione, thiol‐containing proteins) leading to modulation of their concentration and activity in the physiological media. Since the main reducing agent present in mammalian cells at mM concentrations is the tripeptide glutathione (GSH)^[43]^ we studied the interaction of this class of ruthenium complexes with (*S*)‐cysteine and GSH. The complex **2** ^***R***^ promptly reacts with (*S*)‐cysteine in phosphate buffer solution (PBS) at pH=9 affording a single stereoisomer in which the amino acid is bound through a Ru−S bond, as inferred from NMR measurements, and it was isolated as complex **2** ^***R***^‐***Cys*** in 70 % yield by addition of NaPF_6_ (6 equiv) (Scheme 4).

This reaction occurs easily in water media and involves the formation of the aquo **2**‐**H_2_O** / hydroxo **2**‐**OH** complexes, according to the pH, and subsequent coordination of (*S*)‐cysteine. It is worth noting that this reaction does not take place in common organic solvents, including methanol, indicating that water appears crucial for the acetate substitution, as also evidenced by DFT calculations (see further part). The ^31^P{^1^H} NMR spectrum of **2** ^***R***^‐**Cys** in CD_3_OD displays two doublets at δ 40.5 and 28.9 ppm (^*2*^ *J*_PP_=29.3 Hz) for the P atoms *trans* to N and S ones, respectively. In the ^1^H NMR spectrum, the doublet of doublets at δ 2.89 ppm corresponds to the cysteine CH group, while the signals at δ 1.75 and 0.98 ppm are attributed to the diastereotopic CH_2_ protons of the amino acid. The attribution of P atoms is consistent with the 2D ^31^P‐^1^H HMBC spectrum, where the doublet at δ_P_ 28.9 ppm shows a long range coupling with the CH_2_ cysteine protons. In the ^13^C{^1^H} NMR spectrum, the CO carbon appears as a doublet of doublets at δ 205.6 ppm (^*2*^ *J*_CP_=20.4 and 11.6 Hz), whereas the doublet at δ 57.2 (^4^ *J*_CP_=3.3 Hz) and the singlet at δ 28.0 ppm are for the cysteine CH and CH_2_ carbon atoms, respectively, as inferred from 2D ^13^C‐^1^H HSQC spectrum. Similarly, treatment of **2** ^***S***^ with (*S*)‐cysteine in the presence of NaPF_6_ gives **2** ^***S***^‐**Cys** isolated in 65 % yield, displaying two doublets at δ_P_ 40.3 and 29.0 ppm, close to those of **2** ^***R***^‐**Cys** (Eq. (2)). 
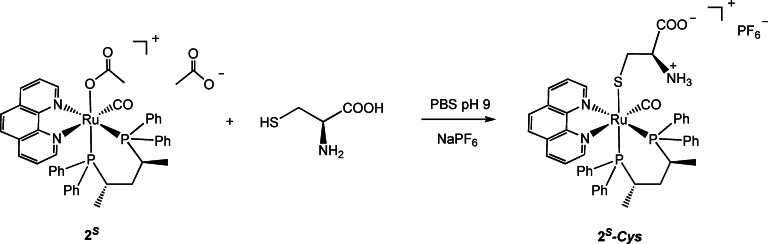


Conversely, in ^1^H NMR spectrum the cysteine signals of **2** ^***S***^‐**Cys** are at δ 2.52 ppm for the CH and δ 1.54 and 1.32 ppm for the CH_2_ protons, which significantly differ from the diastereoisomer **2** ^***R***^‐**Cys**. The NOESY ^1^H‐^1^H 2D NMR spectra of both complexes exhibit NOE effect between the low field *ortho* phen and the CH_2_ protons of cysteine. By lowering the pH in the range 5.0–7.5 the ^31^P{^1^H} NMR measurements of **2** ^***R***^‐**Cys** and **2** ^***S***^‐**Cys** reveal the formation of additional protonated species in equilibrium (Figure S22). Interestingly, complexes **2** ^***R***^ and **2** ^***S***^ quickly react with 1 equivalent of GSH with quantitative formation of the corresponding diastereoisomers **2** ^***R***^‐**SG** (δ_P_ 40.4 and 29.8 ppm) and **2** ^***S***^‐**SG** (δ_P_ 40.6 and 30.0 ppm), respectively, in PBS solutions at pH 9, in agreement to the reaction with (*S*)‐cysteine. Therefore, these results suggest that **2** ^***R***^ and **2** ^***S***^ can easily react with proteins exhibiting cysteine residues at physiological pH.

### DFT Calculations

#### Stability of 2^*R*^

**Figure 4:**
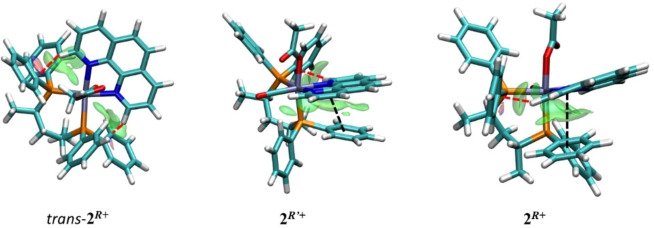
Minimum energy structures with the δg^inter^ isosurfaces (isovalue 0.0055 a.u.) for *trans*‐**2** ^***R+***^ and the *cis* isomers **2** ^***R’+***^ and **2** ^***R+***^. Nonbonded interactions: CH−C (red dashed lines), π–π (black dashed lines).

The minimum energy structures of the three possible isomers of [Ru(η^1^‐OAc)(CO)((*R,R*)‐Skewphos)(phen)]^+^ (**2** ^***R*** +^) are shown in Figure 4. The intra‐molecular non‐bonded interactions are evidenced by the spatial distribution of the δg^inter^ descriptor.[^44^, ^45^]

The calculated energies in methanol show that the isomer **2** ^***R+***^ is more stable than **2** ^***R***^’^+^ and *trans*‐**2** ^***R+***^, being G_**2**_^***R+***^–G_**2**_^***R’+***^=−4.6 kcal mol^−1^ and G_**2**_^***R+***^–G_t*rans*‐**2**_^***R+***^=−7.2 kcal mol^−1^. These results agree with the NMR spectra, which show the formation of the thermodynamically most stable **2** ^***R***^ as a single stereoisomer. Non‐covalent interactions are observed between the (*R*,*R*)‐Skewphos phenyls and the phen ligand (δg^inter^ peaks at low sign(λ_2_)ρ in Figure S30) corresponding to CH−C interactions for the three species, while the *cis* isomers **2** ^***R’+***^ and **2** ^***R+***^ display additional π–π interactions (Figure 4 and Figure S30). The bond lengths Ru−P1and Ru−P2 are nearly equal in **2** ^***R+***^ (2.374 and 2.379 Å) while for *trans*‐**2** ^***R+***^ and **2** ^***R’+***^ the difference between the Ru−P1 and Ru−P2 distance are 0.05 and 0.01 Å, respectively (Table S1 and Figure S31). The Ru−N1 and Ru−N2 (*trans* to CO and P, respectively) distances are 2.219 and 2.144 Å for **2** ^***R+***^, similar to those of **2** ^***R’+***^, in agreement with strong *trans* influence of CO with respect to the phosphine, whereas for *trans*‐**2** ^***R+***^ the Ru−N1 and Ru−N2 are 2.180 and 2.187 Å. DFT calculations show that the diphosphine adopts a distorted boat conformation, with axial and pseudo‐equatorial CH_3_ groups in the three isomers (Figure S32).

### Hydrolysis of 2^*R*^

**Figure 5:**
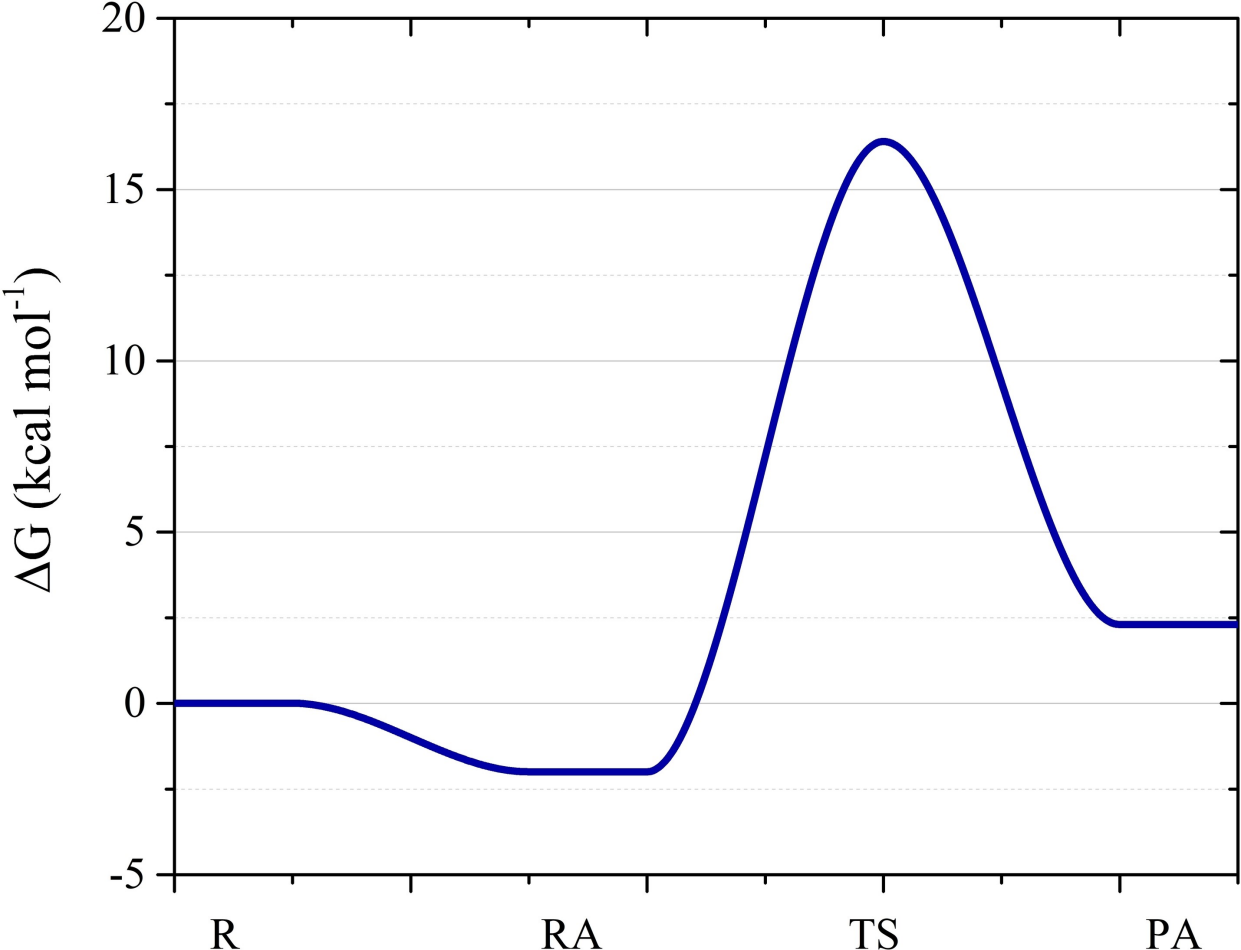
Free energy profile for the hydrolysis reaction leading to the formation the aquo **2** ^***R***^ **‐H_2_O^++^**‐ species. The data for the reaction leading **2** ^***R***^ **‐OH^+^** are reported in Table S2. The energies of the separated reactants (R) are taken as reference.

Experimental data show that the reactivity of **2** ^***R***^ is enhanced by the interaction with water through the formation of the aquo **2** ^***R***^‐**H_2_O** and hydroxo **2** ^***R***^‐**OH** species (Scheme 3). The minimum energy structure of the **2** ^***R+***^ **+H_2_O** reactant adduct (RA) presents the water molecule interacting via hydrogen bond with the acetate group that stabilizes the system with G_**2*R+***‐**H2O**_–G_**2*R+***_=−2.0 kcal mol^−1^, as reported in Figure 5 (see also Figure S33 and Table S2).

**Figure 6:**
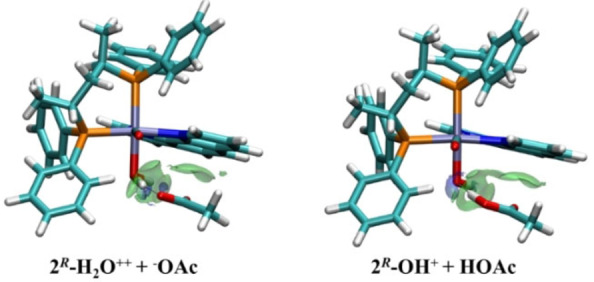
Minimum energy structures for the PA of aquo **2** ^***R***^ **‐H_2_O^++^+^−^OAc** and the hydroxo **2** ^***R***^ **‐OH^+^+HOAc** complexes. δg^inter^ isosurfaces (isovalue 0.0055 a.u.) in green.

The vibrational mode, with imaginary frequency in the transition state (TS), corresponds to the rupture of the Ru‐OAc bond and the simultaneous formation of Ru‐OH_2_ bond, with an activation energy ΔG^≠^=G_TS_–G_***R*** **A**_=18.4 kcal mol^−1^ (Table S2 and Figure S33). This ΔG^≠^ is similar to that observed for the hydrolysis of [(η^6^‐*p*‐cymene)RuCl(methyl 1‐butyl‐2‐arylbenzimidazolecarboxylate)] (18.8 kcal mol^−1^)^[46]^ and lower compared to the calculated[^47^, ^48^] and experimental (ΔG^≠^∼23 kcal mol^−1^)^[49]^ values for NAMI−A. In the aquo **2** ^***R***^‐**H_2_O^++^** product adduct (PA, Figure S33) the coordinated water interacts with the external acetate, while the transfer of the water proton to acetate results in the formation of acetic acid and the hydroxo **2** ^***R***^‐**OH^+^** species, leading in both cases to strong hydrogen‐bond interactions (Figure 6). The reaction profile shows that **2** ^***R***^‐**H_2_O^++^** and **2** ^***R***^‐**OH^+^** are isoenergetic (G_**2**_^***R***^_‐**OH**_^**+**^−G_**2**_^***R***^_‐**H2O**_^**++**^∼0.1 kcal mol^−1^, Table S2), indicating that these two species coexist in solution. The overall reaction results thermodynamically slightly disfavored (ΔG=+2.3 and+2.4 kcal mol^−1^, Table S2), in line with the NMR data of **2** ^***R***^ in water, as observed for the hydrolysis of related complexes.^[46]^

### Reactions of 2^*R*+^, 2^*R*^‐H_2_O^++^ and 2^*R*^‐OH^+^ with (*S*)‐cysteine

**Figure 7:**
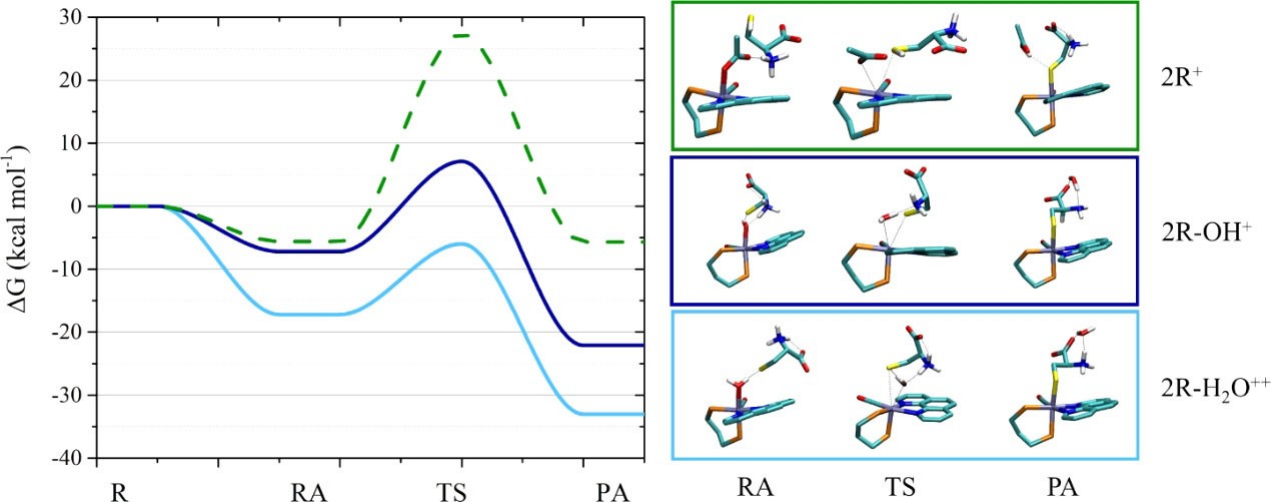
Left: free energy profiles in basic condition for the reactions of the thiol‐deprotonated (*S*)‐cysteine with **2** ^***R***^ **‐H_2_O^++^** (light blue); **2** ^***R***^ **‐OH^+^** (blue). The energy profile for the reaction between zwitterionic *(S)‐*cysteine and **2R^+^** is also reported (green dashed line). Right: structures of the RA, TS and PA (some atoms were removed for clarity, complete structures in Figure S34).

In the case of **2^R+^** (Figure 7), the ΔG^≠^ is significantly higher with respect to the acetate replacement by water (ΔG^≠^=32.9 vs. 18.4 kcal mol^−1^). This result supports a reaction model where the coordination of (*S*)‐cysteine proceeds after the hydrolysis of **2** ^***R*** **+**^ (Figure 6, Table S2). As reported above, **2** ^***R***^ reacts with (*S*)‐cysteine in water at pH=9.0 giving **2** ^***R***^‐**Cys** where the thiol group is coordinated to the metal. The comparison of the energy profile for the reaction of the Ru complexes with the thiol‐deprotonated (*S*)‐cysteine (Scheme S1) are shown in Figure 7. The formation of RA is thermodynamically favored in both cases, due to the formation of H‐bonds between thiolate group of (*S*)‐cysteine and H_2_O or OH ligand (Figure S34, Table S2). The greater stabilization of RA between **2** ^***R***^‐**H_2_O^++^** and (*S*)‐cysteine compared to **2** ^***R***^‐**OH^+^** is likely related to the different charge of the complexes, +2 and +1, respectively. The TS of **2** ^***R***^‐**H_2_O^++^** presents two H‐bonds between the H_2_O and (*S*)‐cysteine (Table S2), while in the TS of **2** ^***R***^‐**OH^+^** a proton transfer from ‐NH_3_^+^ to OH occurs (Figure S34). The formation of Ru−S‐(*S*)‐cysteine‐NH_3_^+^ product from **2** ^***R***^‐**H_2_O^++^** and the thiol is the kinetically (ΔG^≠^=11.2 kcal mol^−1^) and thermodynamically favored reaction path.

**Figure 8:**
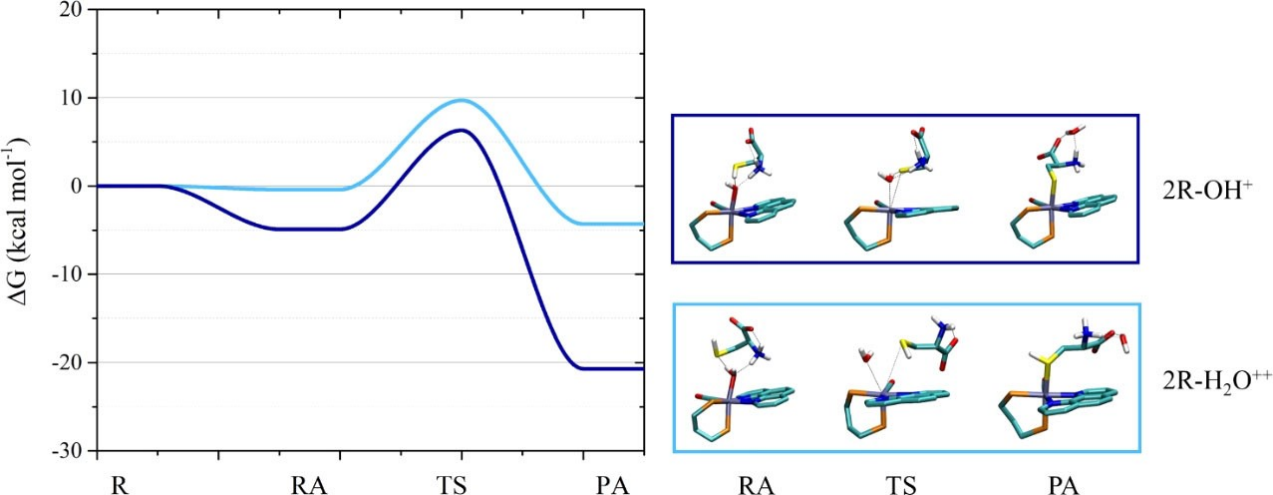
Left: reaction energy profiles in acidic‐neutral conditions for the reactions between (*S*)‐cysteine and **2** ^***R***^ **‐H_2_O^++^**, (light blue), **2** ^***R***^ **‐OH^+^** (blue). Right: structures of the RA, TS and PA (some atoms were removed for clarity, complete structures in Figure S35).

The reaction energy profiles for the ligand replacement in **2** ^***R***^‐**H_2_O^++^** and **2** ^***R***^‐**OH^+^** by the zwitterionic form of (*S*)‐cysteine which is prevalent at acidic‐neutral pH (Scheme S1)^[50]^ are shown in Figure 8 (Table S2) and the optimized structures of RA, TS and PA species are reported in Figure S35.

The formation of RA species with (*S*)‐cysteine is thermodynamically favored for **2** ^***R***^‐**OH^+^**, while no significant stabilization is observed in the case of **2** ^***R***^‐**H_2_O^++^** (Figure 8, Table S2). The substitution of water in **2** ^***R***^‐**H_2_O^++^** by the SH group of (*S*)‐cysteine is kinetically favored with respect to the same reaction for OH^−^ in **2** ^***R***^‐**OH^+^** (ΔG^≠^=10.1 kcal mol^−1^ and 17.4 kcal mol^−1^, respectively). It is worth pointing out that in the TS of **2** ^***R***^‐**OH^+^** a proton transfer from SH group of (*S*)‐cysteine to OH group occurs, with water as leaving group (Figure S35, Table S2).^[51]^ However, the formation of the Ru−S‐(*S*)‐cysteine product from **2** ^***R***^‐**OH^+^** is thermodynamically favored (Figure 8, S35, Table S2).

The path leading to the formation of Ru‐OOC‐(*S*)‐cysteine‐NH_3_^+^ from **2** ^***R***^‐**H_2_O^++^** is kinetically favored with respect to that starting from **2** ^***R***^‐**OH^+^** (ΔG^≠^=9.7 kcal mol^−1^ and 15.5 kcal mol^−1^, respectively, Table S2 and Figure S36) while the relative stability of the final PAs is very similar, consistent with presence of multiple species at lower pH observed by NMR. The Ru−S‐(*S*)‐Cysteine‐NH_3_^+^ product results to be the most stable among the possible products considered but presents the highest ΔG^≠^.

The relative stability of the two diastereoisomers obtained by reaction of **2** ^***R+***^ with protonated and deprotonated (*R*)‐ and (*S*)‐cysteine (Figure S37) has been evaluated. The complex **2** ^***R+***^ presents similar interaction with HS‐(*S*)‐cysteine and HS‐(*R*)‐cysteine, the structures being almost isoenergetic (ΔG=G_**2**_^***R*** **+**^_‐**(*S*)**‐***HS***‐**cysteine**_−G_**2**_^***R*** **+**^_‐**(*R*)**‐***HS***‐**cysteine**_=0.5 kcal mol^−1^). Conversely, a slightly different interaction has been observed for **2** ^***R+***^ with the deprotonated (*S*)‐cysteine with respect to (*R*)‐ species (ΔG=G_**2**_^***R*** **+**^_‐**(*S*)**‐_^**−**^_**S**‐**cysteine**_−G_**2**_^***R*** **+**^_‐**(*R*)**‐_^**−**^_**S**‐**cysteine**_=−1.2 kcal mol^−1^).

### Biological activity of the ruthenium complexes

#### Solution stability of the complexes 2^*R*^‐4^*R*^ and 2^*S*^‐4^*S*^ over time

**Figure 9:**
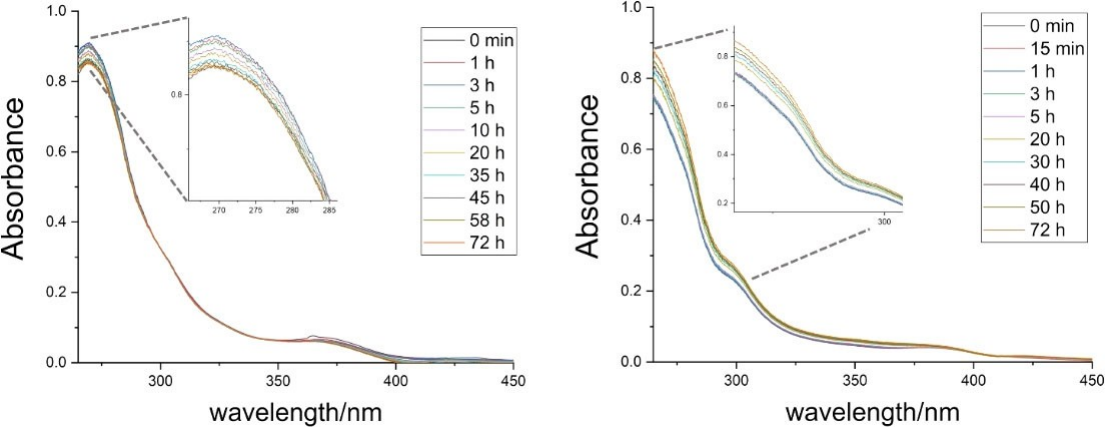
Selection of electronic spectra of the complex **2** ^***R***^ (left) and **4** ^***R***^ (right) in DMEM (25 % v/v) acquired during a 72‐h scanning kinetics.

Prior to in vitro cell testing, the stability of each chiral compound (*S* enantiomers taken as a model) has been checked in different media, starting from the organic solvent DMSO (vehicle approved for clinical use, if properly diluted). The analysis has been carried out in aqueous media endowed with increasing complexity and biocompatibility (i. e., deionized water, 0.9 % NaCl w/v saline solution and PBS). Afterwards, the complete DMEM (Dulbecco's Modified Eagle Medium supplemented with 10 % v/v FBS) has been considered for all the six complexes, in light of the chiral nature of the components (e. g., amino acids, vitamins, growth factors, saccharides). Each obtained scanning kinetics is reported in Supporting Information (Figures S38–S53). The Figures S38–S40 report a selection of UV‐Vis spectra collected in DMSO, overlapped over 72 h. The most intense band at ca. 270 nm and the shoulder at about 300 nm have been ascribed to intraligand π→π* transitions (Figures S54 and S55 display the UV‐Vis spectra of the free ligands in DMSO and in water). The band at around 375 nm is likely due to a M−L charge transfer.^[52]^ On passing from the spectra collected in pure DMSO (where all compounds are stable) to those in water, the complexes **2** ^***S***^ and **4** ^***S***^ and the related aquo/hydroxo species have proved stable over 72 h, whereas **3** ^***S***^ has been associated with an hyperchromic effect, occurring after 5 h. No bathochromic or hypsochromic shift has been detected, thus highlighting no significant change of the compound structure. Interestingly, the spectrum of **3** ^***S***^ shows no changes in saline solution (NaCl 0.9 % w/v) over 72 h (Figure S45). By contrast, **4** ^***S***^ has showed an absorbance decrease in saline solution after the first 5 h with a slight precipitate, as also observed in PBS after 3 h (Figure S49), as a result of the counterion or ligand (carboxylate, OH, H_2_O) replacement. A hypochromic shift has been observed also for **2** ^***S***^ and **3** ^***S***^ in PBS but to a lesser extent. It is worth pointing out that in complete DMEM (25 % v/v) the aquo/hydroxo species of the (*S*)‐enantiomers are stable over time (Figures S50‐52), while the spectra of the (*R*)‐enantiomers in the same medium do not overlap during the kinetics. As a matter of fact, under these physiological conditions **3** ^***R***^ and **4** ^***R***^ show a hyperchromic effect, while **2** ^***R***^ displays a hypochromic effect, pointing out a change in the electronic transition probability, due to the formation of new species (Figure 9 and Figure S53).

In this regard, the higher cytotoxicity of the *R* enantiomers, namely complexes **2** ^***R***^ and **4** ^***R***^ and the corresponding hydroxo and aquo complexes, compared to the *S* derivatives (Table 1), may be related to their larger reactivity in the cell culture medium (Figure 9), leading to adducts with biomolecules (i. e. sugars, vitamins, serum albumin and growth factors), thus highlighting the key role of the transition metal complex chirality.

**Table 1:** EC_50_ (μM ± SD) of the complexes **2** ^***R***^/**2** ^***S***^, **3** ^***R***^/**3** ^***S***^, **4** ^***R***^/**4** ^***S***^ and cisplatin in SW1736, 8505 C, HCT‐116 and A549 cell lines.

	Human cancer cell lines^[a]^			
Complex	SW1736	8505 C	HCT‐116	A549
**2^R^**	0.29±0.03	1.4±0.2	0.24±0.05	0.9±0.1
**2^S^**	2.0±0.1	2.3±0.3	1.2±0.1	2.8±0.6
**3^R^**	1.35±0.04	0.35±0.02	0.81±0.08	1.9±0.8
**3^S^**	2.3±0.2	0.7±0.1	0.9±0.1	1.66±0.04
**4^R^**	0.7±0.1	0.04±0.01	0.37±0.09	0.54±0.05
**4^S^**	1.28±0.09	0.58±0.05	1.1±0.3	2.51±0.01
**Cisplatin**	6±2	5±2	5.7±0.2	3.6±0.7

[a] Each value represents the mean value of at least three‐fold determinations after a 72‐h treatment.

### Effects of the enantiomeric ruthenium complexes on cell viability

The effectiveness of the three pairs of enantiomeric ruthenium complexes **2** ^***R***^/**2^S^**, **3** ^***R***^/**3** ^***S***^ and **4** ^***R***^/**4** ^***S***^ was first evaluated in anaplastic thyroid cancer (ATC) cell lines (SW1736 and 8505 C), and their results have been compared with those of cisplatin. To test the effects of the chiral complexes on the viability of ATC cells, an MTT assay has been performed after the administration of different doses of the complexes for 24, 48 and 72 h. The EC_50_ values (the concentration of the test complex inducing 50 % reduction in cell number compared with control cultures) have been calculated at 72 h and reported in Table 1. Regarding the incubation time, 72‐h treatments are commonly carried out in preliminary screening tests wherein cisplatin is used as a reference drug due to its slow ligand‐substitution kinetics.^[53]^ Interestingly, the chirality at the metal center has a strong influence on the activity towards these two cancer cell lines, these ruthenium complexes being considerably more active than cisplatin. As a matter of fact, all the (*R*,*R*)‐enantiomers reduce the cell viability at significantly lower concentrations than the corresponding (*S*,*S*)‐derivatives.

The complex **2** ^***R***^ is almost 7 times more active than **2** ^***S***^, exhibiting EC_50_ values of 0.29 and 1.98 μM in the SW1736 cells, respectively. Surprisingly, the derivative **4** ^***R***^ reaches the lowest EC_50_ ever observed in this study, achieving 0.04 μM of EC_50_ in the 8505 C ATC cell line, a value more than 14 times lower with respect to the corresponding enantiomer **4** ^***S***^ (0.58 μM). By contrast, the pivalate derivative **3** ^***R***^ does not exhibit a great difference in activity compared to **3** ^***S***^, with EC_50_ values of 0.35 and 0.68 μM in the 8505 C cell line, respectively. The chiral complexes have been further investigated against the HCT‐116 and A549 cancer cell lines and the obtained EC_50_ values are collected in Table 1. It is worth pointing out that all compounds are more active than cisplatin and **2** ^***R***^ and **4** ^***R***^ are 4‐to‐7‐fold more cytotoxic against A549 cells and 15‐to‐24‐fold more active against HCT‐116 cells. Interestingly, as for the ATC cell lines, the (*R*,*R*)‐enantiomers display the most promising antitumor activity, with the **2** ^***R***^ and **4** ^***R***^ derivatives exhibiting 3‐to‐5‐fold lower EC_50_ values, compared to the (*S*,*S*) counterparts in both the HCT‐116 and A549 cell lines. Conversely, the pivalate enantiomers **3** ^***R***^ **/3** ^***S***^ showed comparable EC_50_ data, in line with those obtained with the ATC cells. For comparison reasons, the ligands phen and the (*R,R*)‐ and (*S,S*)‐Skewphos were tested in vitro under the same experimental conditions. Briefly, the NN ligand phen proved more cytotoxic against the HCT‐116 cell line (EC_50_ in the range 1÷10 μM) compared to the A549 cell line (50 μM<EC_50_<100 μM). Interestingly, once again chirality is fundamental when studying biological systems. Thus, the (*S,S*)‐Skewphos ligand has been found more cytotoxic against A549 cells (50 μM<EC_50_<100 μM) compared to the (*R*,*R*) one, which shows an EC_50_ value higher than 100 μM. By contrast, the (*R*,*R*)‐enantiomer diphosphine is more active against HCT‐116 cells (10 μM<EC_50_<25 μM) compared to the (*S*,*S*)‐ligand (50 μM<EC_50_<100 μM), clearly indicating that chirality affects the cytotoxicity and that the ruthenium complexes display higher activity compared to the free ligands.

### Effects of enantiomeric ruthenium complexes on cell colony forming ability

**Figure 10:**
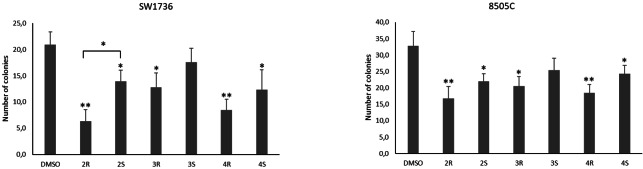
Enantiomeric ruthenium complexes affect the colony formation ability of ATC cells. Colony formation in SW1736 (A) and 8505 C (B) cells treated with enantiomeric ruthenium complexes couples or vehicle (DMSO) for 48 h. Representative bar chart of the number of colonies after 21 days. Each compound was administered at its own EC_50_. *P<0.05, **P<0.01 by the Student's t‐test. All data are representative of three independent experiments.

To assess the effects of the enantiomeric ruthenium complexes on marker of aggressiveness in the two ATC cell lines, its influence on the ability of cells to form colonies in an anchorage‐independent manner was analyzed using a soft‐agar colony formation assay. As shown in Figure 10, we have observed a significant reduction in the number of colonies in cells treated with the ruthenium complexes, each used at its own EC_50_, except for **3** ^***S***^, compared to those treated with DMSO alone.A trend can be observed, according to which the (*S*,*S*)‐enantiomers have a less effectiveness in reducing the number of colonies, although still used at their EC_50_. In SW1736 cells the enantiomers **2** ^***R***^/**2** ^***S***^ show significantly different effects, with the **2** ^***S***^ enantiomer inducing a significantly smaller reduction in the number of colonies compared to **2** ^***R***^.

### Effects in terms of cell death, morphology and migration

**Figure 11:**
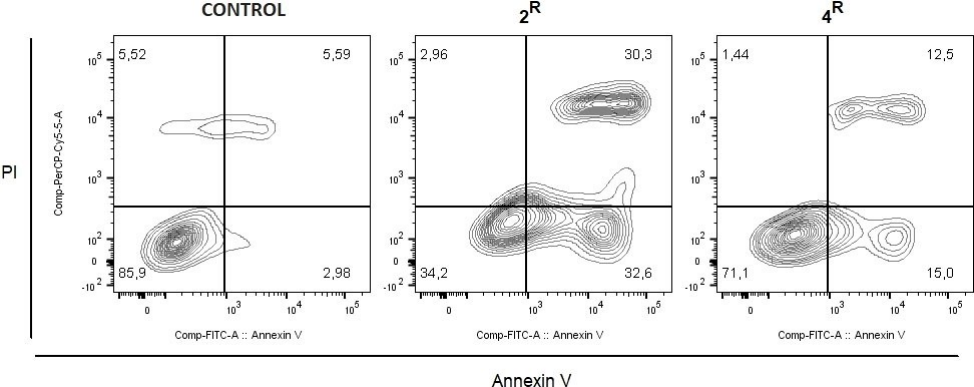
Flow cytofluorimetry assay of the complexes **2** ^***R***^ and **4** ^***R***^. Percentages of viable (lower left), apoptotic (lower and upper right), and necrotic (upper left) cells are reported in the corner of each quadrant.

To check whether cell death occurs via apoptosis, we carried out an Annexin V/Propidium Iodide (PI) assay. Considering 0.5 μM as EC_50_ cut‐off value, we have chosen the HCT‐116 cell line for this investigation. As a matter of fact, Table 1 points out that only against colon carcinoma cells the complexes **2** ^***R***^ and **4** ^***R***^ have comparable antiblastic activity. HCT‐116 cells have been hence treated at 0.5 μM with the selected compounds for 72 h. Then, cells have been harvested and labeled with Annexin‐V FITC and PI prior to flow cytometry, aimed at evaluating the percentage of apoptotic cells. In these experiments, apoptotic cells at early stage occur in the lower right quadrant, while those at late stage set in the up‐right part. The percentage in the lower left quadrant represents viable cells whereas the upper left part corresponds to cells undergoing non‐apoptotic cell death. Remarkably, the number of cells undergoing non‐apoptotic cell death is comparable for both treatments to the vehicle (DMSO) control. Both complexes trigger apoptosis with similar percentages between the early‐stage‐apoptosis cell population and the late‐stage one (Figure 11).

The **2** ^***R***^‐treated sample is associated with the highest percentage of apoptotic cell death (total 62.9 %), confirming its greater potency when compared to **4** ^***R***^ (total 27.5 % of cells undergoing apoptosis) in this cell line.

**Figure 12:**
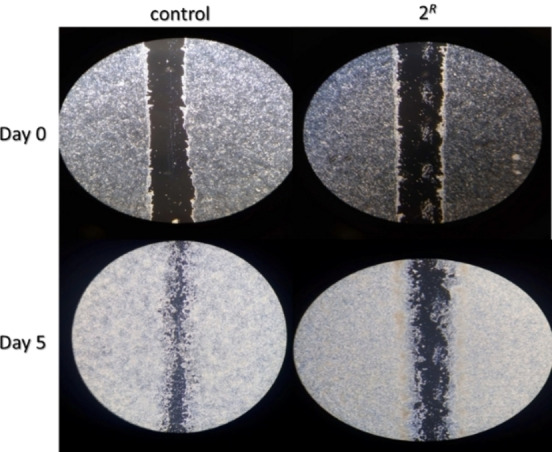
Cell migration ‐ scratch assay: collection of microscope (Leica DMi1; inverted phase contrast) images (4X) concerning day 0 and day 5 after treatment with and without (Control) complex **2** ^***R***^.

Regarding cell morphology, treatment of HCT‐116 cells with **2** ^***R***^ and **4** ^***R***^ complexes (72 h at 0.5 μM) decreased cell proliferation. In both cases cells change shape as well as apoptotic bodies and cell debris are visible. In addition, **2** ^***R***^ induced cell disaggregation whereas the **4** ^***R***^ compound did not (Figure S56). Cell migration and invasion are key phenomena in physiologic and pathologic processes, such as wound healing and cancer metastasis. To test whether the **2** ^***R***^ complex affects cell migration, cells have been seeded in a Petri dish and allowed to attach, spread and form a confluent monolayer. A pin tool or needle is usually exploited to scratch and remove cells from a discrete area of the confluent monolayer so to form a cell‐free zone.[^54^, ^55^] We examined cell migration in response to the mechanical scratch wound, carried out after treatment (or not, control) with the model compound **2** ^***R***^ (Figure 12). Additional microscope images pointed out – after a 24‐h treatment at 3 μM of HCT‐116 cells – a reduction of about 30 % of the cell migration rate on the fifth day (Figure S57).

Therefore, the cells previously treated with the selected Ru(II)‐based compound migrated at the edges of the wound to a lesser extent compared to the control.

### Log *P* evaluation

In light of the very promising data collected in vitro, it was of paramount importance to evaluate the *n*‐octanol‐water partition coefficient (log *P*) to shed light on the capability of this class of compounds to pass through the biological barriers (e. g., cell membrane, gastrointestinal barrier).^[56]^ In addition, the collected values point out possible scenarios in terms of future nanoformulation for advanced preclinical testing.^[57]^ We considered the (*S,S*)‐enantiomers as model compounds and the results are collected in Table 2.

**Table 2:** Log *P* values of the chiral ruthenium complexes **2** ^***S***^, **4** ^***S***^ and **3** ^***S***^, as *n*‐octanol/water partition coefficient.

Complex	Log *P* (pH 7; 25 °C)
**2^S^**	−0.15±0.02
**3^S^**	+0.56±0.06
**4^S^**	+0.04±0.01

Despite the same molecular design, metal‐to‐ligand stoichiometry and ionic character, the studied complexes display quite different log *P* values. In fact, the positive recorded value for the compound **3** ^*S*^ highlights its hydrophobic nature. Conversely, the complex **2** ^*S*^ bearing acetate as a ligand and counterion is associated with a negative log *P* value. A value around zero was recorded for the compound **4** ^*S*^. Taken together, the nature of the counterion/ligand reflects an increasing lipophilic character in the order **2** ^***S***^<**4** ^***S***^<**3** ^***S***^. These results underline the possibility for this new class of complexes to show affinity to both the phospholipidic layer of cell membrane and hydrophilic physiological conditions, *conditio sine qua non* for future biological applications.

## Conclusions

In summary, we have described the preparation of the chiral cationic complexes of formula [RuX(CO)(diphosphine)(phen)]Y, bearing (*R,R*)‐ and (*S,S*)‐Skewphos as diphosphine, carboxylate, thioacetate and thiolate as X ligand and RCO_2_^−^ and PF_6_^−^ as Y counterion. These derivatives have been easily obtained as single stereoisomers in high yield from [Ru(η^1^‐OAc)(η^2^‐OAc)(diphosphine)(CO)] and phen, followed by acetate substitution, their structure being established by NMR and DFT studies. The carboxylate complexes display facile displacement of the RCO_2_^−^ ligand with (*S*)‐cysteine in water due to the formation of reactive aquo/hydroxo species via hydrogen bond interactions as established by DFT calculations which also provide rather similar energy for the two cysteine diastereoisomers [Ru((*S*)‐Cys)(CO)(Skewphos)(phen)]PF_6_. The derivatives [RuX(CO)(diphosphine)(phen)]Y inhibit cancer cell proliferation and colonization and display high cytotoxic activity in the range of 2.8–0.04 *μ*M against the SW1736, 8505 C, HCT‐116 and A549 cell lines, strongly depending on the chirality at metal center. As a matter of fact, the thioacetate complex [Ru(η^1^‐SAc)(CO)((*R,R*)‐Skewphos)]OAc (**4** ^***R***^) shows an EC_50_ value of 0.04 *μ*M for the anaplastic thyroid cancer cell line 8505 C, for which no effective treatments are available. This value is significantly lower than that of Cisplatin and it is 14 times lower with respect to its enantiomer **4** ^***S***^. On account of this straightforward synthetic protocol, entailing the use of commercial chiral diphosphines, this class of air stable [RuX(CO)(PP)(phen)]Y complexes appears attracting for applications as metallodrugs. Ongoing studies are focused on improving their antitumor activity through the combination of suitable ligands and investigating the mechanism of action.

## Experimental Section

**General**: All reactions were carried out under an argon atmosphere by using standard Schlenk techniques. The precursors [Ru(η^1^‐OAc)(η^2^‐OAc)(PP)(CO)] (PP=(*R,R*)‐Skewphos, **1** ^***R***^; (*S,S*)‐Skewphos, **1** ^***S***^) were prepared according to literature procedures,^[30]^ whereas (*R,R*)‐Skewphos, (*S,S*)‐Skewphos, phen and all other chemicals and solvents were purchased from Aldrich and Strem and used without further purification. NMR measurements were performed using a Bruker Advance III HD NMR 400 MHz spectrometer and the chemical shifts, in ppm, are internally referred to TMS for ^1^H and ^13^C{^1^H} NMR and 85 % H_3_PO_4_ for ^31^P{^1^H} NMR. Elemental analyses (C, H, and N) were carried out with a Carlo Erba 1106 elemental analyzer, whereas IR analyses were performed with a Bruker Vector 22 FTIR spectrometer. The electronic spectra of the ligands and the compounds (evaluation of the *n*‐octanol/water partition coefficient, log *P*) were recorded by the Evolution 201 (Thermo Fisher Scientific, Inc.) UV‐Visible spectrophotometer. Adopted conditions: wavelength range: 190–500 nm, dual beam mode, scan rate 400 nm/min, integration time 0.30 sec, data range: 1 nm. The solution stability tests of the compounds were performed by serial acquisition of electronic spectra in the UV‐Vis domain, generally from 265 nm to 500 nm, by means of the spectrophotometer Cary 60 (Agilent Technologies). Experimental set up: scanning time 0.088 sec, scan rate 102.9 nm/min, slit 1 nm, 149 total cycles of which 9 in the first 2 h and 140 in the next 70 h. For the spectrophotometric lecture of the 96‐well plates (cell viability tests) the INFINITE M PLEX (Tecan) equipment was used.

**Synthesis of [Ru(η^1^**‐**OAc)(CO)((*R,R*)**‐**Skewphos)(phen)]OAc (2** ^***R***^ **)**: Complex [Ru(η^1^‐OAc)(η^2^‐OAc)((*R,R*)‐Skewphos)(CO)] (**1** ^***R***^) (100.0 mg; 0.145 mmol) and 1,10‐phenanthroline (26.1 mg; 0.145 mmol) were dissolved in methanol (1.5 mL) and the mixture was stirred at 60 °C overnight. The solvent was evaporated under reduced pressure and the residue was dissolved in dichloromethane (0.5 mL). Addition of diethyl ether (5 mL) afforded a pale‐yellow precipitate, which was filtered off and dried under reduced pressure. Yield: 115 mg (91 %). Elemental analysis (%) calcd for C_46_H_44_N_2_O_5_P_2_Ru: C 63.66, H 5.11, N 3.23; found: C 63.50, H 4.99, N 3.11. ^1^H NMR (400.1 MHz, CDCl_3_, 298 K): δ=8.75 (t, ^3^ *J*_HH_=4.3 Hz, 1H; phen), 8.68 (d, ^3^ *J*_HH_=8.1 Hz, 1H; phen), 8.47 (d, ^3^ *J*_HH_=7.9 Hz, 1H; phen), 8.16 (d, ^3^ *J*_HH_=8.8 Hz, 1H; phen), 8.04 (d, ^3^ *J*_HH_=8.8 Hz, 1H; phen), 7.95‐7.74 (m, 7H; Ph), 7.47 (m, 4H; Ph), 7.38 (m, 2H; aromatic protons), 7.28 (m, 1H; phen), 7.18 (m, 3H; Ph), 7.06 (m, 4H; aromatic protons), 6.79 (t, ^3^ *J*_HH_=8.6 Hz, 2H; Ph), 3.43 (br m, 1H; PCH), 2.91 (br m, 1H; PCH), 2.69 (m, 1H; CH_2_), 2.35–2.08 (m, 1H; CH_2_), 2.02 (s, 3H; COCH_3_) 1.19 (s, 3H; COCH_3_), 1.16 (dd, ^3^ *J*_HP_=15.1 Hz, ^3^ *J*_HH_=7.5 Hz, 3H; CHC*H*_3_), 0.82 (dd, ^3^ *J*_HP_=13.0 Hz, ^3^ *J*_HH_=6.8 Hz, 3H; CHC*H*_3_). ^13^C{^1^H} NMR (100.6 MHz, CDCl_3_, 298 K): δ=204.7 (dd, ^2^ *J*_CP_=20.0 Hz, ^2^ *J*_CP_=15.2 Hz; CO), 177.3 (d; ^3^ *J*_CP_=2.5 Hz; *C*OCH_3_), 177.2 (s; *C*OCH_3_), 154.0‐122.7 (m; aromatic carbon atoms), 37.2 (t, ^2^ *J*_CP_=4.1 Hz; *C*H_2_), 32.8 (d, ^1^ *J*_CP_=30.4 Hz; P*C*H), 24.8 (s; CO*C*H_3_), 23.6 (d, ^4^ *J*_CP_=4.3 Hz; CO*C*H_3_), 22.9 (dd, ^1^ *J*_CP_=31.6, ^3^ *J*_CP_=2.3 Hz; P*C*H), 18.5 (d, ^2^ *J*_CP_=5.9 Hz; CH*C*H_3_), 17.2 (d, ^2^ *J*_CP_=1.8 Hz; CH*C*H_3_). ^31^P{^1^H} NMR (162 MHz, CDCl_3_, 298 K): δ 42.9 (d, ^2^ *J*_PP_=32.2 Hz), 41.0 ppm (d, ^2^ *J*_PP_=32.2 Hz). IR (Nujol): $\tilde{\nu }$ =1957 (s) (C≡O), 1602 (s), 1579 (s) (C=O) cm^−1^.

**Synthesis of [Ru(η^1^**‐**OAc)(CO)((*S,S*)**‐**Skewphos)(phen)]OAc (2** ^***S***^ **)**: Complex [Ru(η^1^‐OAc)(CO)((*S,S*)‐Skewphos)(phen)]OAc (**2** ^***S***^) was prepared following the procedure described for **2** ^***R***^, starting from [Ru(η^1^‐OAc)(η^2^‐OAc)((*S,S*)‐Skewphos)(CO)] (**1** ^***S***^) (100.0 mg; 0.145 mmol), in place of **1** ^***R***^, and 1,10‐phenanthroline (26.1 mg; 0.145 mmol). Yield: 110 mg (87 %). Elemental analysis (%) calcd for C_46_H_44_N_2_O_5_P_2_Ru: C 63.66, H 5.11, N 3.23; found: C 63.52, H 4.97, N 3.09.

**Synthesis of [Ru(η^1^**‐**OPiv)(CO)((*R,R*)**‐**Skewphos)(phen)]OPiv (3** ^***R***^ **)**: Complex **2** ^***R***^ (50.0 mg, 0.058 mmol) was dissolved in degassed methanol (2 mL) and then NaOPiv (71.5 mg, 0.576 mmol, 10 equiv) was added to the solution. The reaction mixture was stirred for 48 h at 60 °C and the solvent was evaporated. Dichloromethane (2 mL) was added and the excess of salt was filtered off, obtaining a solution that was concentrated at almost 0.5 mL evaporating the solvent under reduced pressure. Addition of diethyl ether (2 mL) afforded an orange product that was filtered, washed with diethyl ether (2 x 2 mL) and dried under reduced pressure. Yield: 45.5 mg (82 %). Elemental analysis (%) calcd for C_52_H_56_N_2_O_5_P_2_Ru: C 65.60, H 5.93, N 2.94; found: C 65.47, H 5.82, N 2.75. ^1^H NMR (400.1 MHz, CDCl_3_, 298 K): δ=8.88 (dd, ^3^ *J*_HH_=8.2 Hz, ^4^ *J*_HH_=0.5 Hz, 1H; phen), 8.60 (t, ^3^ *J*_HH_=3.5 Hz, 1H; phen), 8.51 (dd, ^3^ *J*_HH_=8.2 Hz, ^4^ *J*_HH_=1.2 Hz, 1H; phen), 8.27 (d, ^3^ *J*_HH_=8.8 Hz, 1H; phen), 8.06 (d, ^3^ *J*_HH_=8.8 Hz, 1H; phen), 7.93‐7.67 (m, 6H; Ph), 7.48‐7.33 (m, 7H; aromatic protons), 7.31‐7.18 (m, 3H; aromatic protons), 7.16‐7.04 (m, 5H; Ph), 6.96 (t, ^3^ *J*_HH_=8.8 Hz, 2H; Ph), 3.48 (br m, 1H; PCH), 2.91 (m, 1H; PCH), 2.80 (m, 1H; CH_2_), 2.41–2.17 (m, 1H; CH_2_), 1.22 (s, 9H; C(CH_3_)_3_), 1.20 (dd, ^3^ *J*_HP_=15.0 Hz, ^3^ *J*_HH_=7.4 Hz, 3H; CHC*H*_3_), 0.89 (dd, ^3^ *J*_HP_=12.9 Hz, ^3^ *J*_HH_=6.8 Hz, 3H; CHC*H*_3_), 0.05 ppm (s, 9H; C(CH_3_)_3_). ^13^C{^1^H} NMR (100.6 MHz, CDCl_3_, 298 K): δ=204.7 (dd, ^2^ *J*_CP_=20.0 Hz, ^2^ *J*_CP_=15.2 Hz; CO), 184.0 (br s; *C*OC(CH_3_)_3_), 183.6 (s; *C*OC(CH_3_)_3_), 153.9‐123.6 (m; aromatic carbon atoms), 65.9 (s; *C*(CH_3_)_3_), 37.3 (br s; *C*H_2_), 32.7 (d, ^1^ *J*_CP_=30.3 Hz; P*C*H), 28.9 (br s; COC(*C*H_3_)_3_), 27.3 (s; COC(*C*H_3_)_3_), 23.1 (d, ^1^ *J*_CP_=31.2; P*C*H), 18.5 (d, ^2^ *J*_CP_=5.8 Hz; CH*C*H_3_), 16.9 (s; CH*C*H_3_). ^31^P{^1^H} NMR (162 MHz, CDCl_3_, 298 K): δ 43.7 (d, ^2^ *J*_PP_=31.4 Hz), 41.6 ppm (d, ^2^ *J*_PP_=31.4 Hz). IR (Nujol): $\tilde{\nu }$ =1965 (s) (C≡O), 1600 (s), 1556 (s) (C=O) cm^−1^.

**Synthesis of [Ru(η^1^**‐**OPiv)(CO)((*S,S*)**‐**Skewphos)(phen)]OPiv (3** ^***S***^ **)**: Complex [Ru(η^1^‐OPiv)(CO)((*S,S*)‐Skewphos)(phen)]OPiv (**3** ^***S***^) was prepared following the procedure described for **3** ^***R***^, starting from **2** ^***S***^ (50.0 mg, 0.058 mmol), in place of **2** ^***R***^, and NaOPiv (66.0 mg, 0.576 mmol, 10 equiv). Yield: 43 mg (78 %). Elemental analysis (%) calcd for C_52_H_56_N_2_O_5_P_2_Ru: C 65.60, H 5.93, N 2.94; found: C 65.42, H 5.86, N 2.78.

**Synthesis of [Ru(η^1^**‐**SAc)(CO)((*R,R*)**‐**Skewphos)(phen)]OAc (4** ^***R***^ **)**: Complex **2** ^***R***^ (50.0 mg, 0.058 mmol) was dissolved in degassed methanol (2 mL) and KSAc (64.5 mg, 0.565 mmol, 10 equiv) was added to the solution. The reaction mixture was stirred for 24 h at 60 °C. The solvent was evaporated, acetone (2 mL) was added and the excess of salt was filtered off. The resulting solution was concentrated to almost 0.5 mL and addition of *n*‐pentane (2 mL) afforded an orange precipitate, which was filtered, washed with *n*‐pentane (2 x 2 mL) and dried under reduced pressure. Yield: 40 mg (78 %). Elemental analysis (%) calcd for C_46_H_44_N_2_O_4_P_2_RuS: C 62.50, H 5.02, N 3.17; found: C 62.47, H 5.05, N 3.15. ^1^H NMR (400.1 MHz, CDCl_3_, 298 K): δ=8.63 (d, ^3^ *J*_HH_=8.0 Hz, 1H; phen), 8.56 (m, 2H; phen), 8.17 (s, 2H; phen), 7.93 (m, 3H; Ph), 7.81 (br m, 3H; Ph), 7.53–7.25 (m, 8H; aromatic protons), 7.15 (td, ^3^ *J*_HH_=7.9 Hz, ^4^ *J*_HH_=2.4 Hz, 2H; Ph), 7.07 (td, ^3^ *J*_HH_=7.9 Hz, ^4^ *J*_HH_=2.1 Hz, 2H; Ph), 6.97 (t, ^3^ *J*_HH_=8.5 Hz, 2H; Ph), 6.92 (d, ^3^ *J*_HH_=5.3 Hz, 1H; Ph), 6.62 (t, ^3^ *J*_HH_=8.4 Hz, 2H; Ph), 3.35 (br m, 1H; PCH), 3.07 (m, 1H; PCH), 2.63 (m, 1H; CH_2_), 2.28‐2.04 (m, 1H; CH_2_), 2.09 (s, 3H; SCOCH_3_), 1.90 (s, 3H; COCH_3_), 1.09 (dd, ^3^ *J*_HP_=15.0 Hz, ^3^ *J*_HH_=7.4 Hz, 3H; CHC*H*_3_), 0.80 (dd, ^3^ *J*_HP_=12.5 Hz, ^3^ *J*_HH_=6.9 Hz, 3H; CHC*H*_3_). ^13^C{^1^H} NMR (100.6 MHz, CDCl_3_, 298 K): δ=205.5 (dd, ^2^ *J*_CP_=19.5 Hz, ^2^ *J*_CP_=12.2 Hz; CO), 204.1 (s; S*C*OCH_3_), 176.1 (s; *C*OCH_3_), 152.9‐121.8 (m; aromatic carbon atoms), 37.4 (br t; *C*H_2_), 34.3 (d, ^1^ *J*_CP_=28.9 Hz; P*C*H), 33.4 (d, ^4^ *J*_CP_=2.8 Hz; SCO*C*H_3_), 23.3 (s; CO*C*H_3_), 21.9 (d, ^1^ *J*_CP_=28.5; P*C*H), 18.8 (d, ^2^ *J*_CP_=6.1 Hz; CH*C*H_3_), 17.6 (s; CH*C*H_3_). ^31^P{^1^H} NMR (162 MHz, CDCl_3_, 298 K): δ 41.0 (d, ^2^ *J*_PP_=29.6 Hz), 31.0 ppm (d, ^2^ *J*_PP_=29.6 Hz). IR (Nujol): $\tilde{\nu }$ =1967 (s) (C≡O), 1620 (s), 1587 (s) (C=O) cm^−1^.**Synthesis of [Ru(η^1^**‐**SAc)(CO)((*S,S*)**‐**Skewphos)(phen)]OAc (4** ^***S***^ **)**: Complex [Ru(η^1^‐SAc)(CO)((*S,S*)‐Skewphos)(phen)]SAc (**4** ^***S***^) was prepared following the procedure described for **4** ^***R***^, starting from **2** ^***S***^ (50.0 mg, 0.058 mmol), in place of **2** ^***R***^, and KSAc (63.4 mg, 0.565 mmol, 10 equiv). Yield: 38.5 mg (75 %). Elemental analysis (%) calcd for C_46_H_44_N_2_O_4_P_2_RuS: C 62.50, H 5.02, N 3.17; found: C 62.49, H 5.00, N 3.14.

**Synthesis of [Ru((*S*)**‐**Cys)(CO)((*R,R*)**‐**Skewphos)(phen)]PF_6_ (2** ^***R***^‐**Cys)**: Complex **2** ^***R***^ (50.0 mg, 0.058 mmol) was dissolved in PBS at pH 9 (4 mL) and (*S*)‐cysteine (7 mg, 0.058 mmol) was added to the solution. The reaction mixture was stirred for 1 h at 50 °C and NaPF_6_ (58.5 mg, 0.348 mmol, 6 equiv) was added, resulting in the formation of a fine suspension. The solvent was completely evaporated and water (2 mL) was added to the residue. The obtained solid was filtered, washed with cold water (2 x 2 mL) and dried under reduced pressure. Yield: 41 mg (70 %). Elemental analysis (%) calcd for C_45_H_44_F_6_N_3_O_3_P_3_RuS: C53.26, H 4.37, N 4.14; found: C 53.23, H 4.38, N 4.11. ^1^H NMR (400.1 MHz, CD_3_OD, 298 K): δ=8.63 (d, ^3^ *J*_HH_=8.2 Hz, 1H; phen), 8.60 (m, 1H; phen), 8.46 (d, ^3^ *J*_HH_=8.0 Hz, 1H; phen), 8.25 (t, ^3^ *J*_HH_=8.3 Hz, 2H; Ph), 8.11 (d, ^3^ *J*_HH_=8.8 Hz, 1H; phen), 8.01 (d, ^3^ *J*_HH_=8.8 Hz, 1H; phen), 7.99–7.89 (m, 3H; Ph), 7.85 (td, ^3^ *J*_HH_=7.6 Hz, ^4^ *J*_HH_=2.1 Hz, 2H; phen), 7.71–7.48 (m, 4H; aromatic protons), 7.44 (td, ^3^ *J*_HH_=7.5 Hz, ^4^ *J*_HH_=3.5 Hz, 1H; Ph), 7.34 (dd, ^3^ *J*_HH_=8.1 Hz, ^4^ *J*_HH_=5.4 Hz, 1H; phen), 7.31–7.17 (m, 4H; Ph), 7.10‐6.98 (m, 4H; Ph), 6.75 (t, ^3^ *J*_HH_=8.4 Hz, 2H; Ph), 3.48 (br m, 1H; PCH), 3.16 (m, 1H; PCH), 2.89 (dd, ^3^ *J*_HH_=9.9 Hz, ^3^ *J*_HH_=3.5 Hz, 1H; CH Cys), 2.67 (m, 1H; CH_2_), 2.36‐2.11 (m, 1H; CH_2_), 1.75 (dt, ^2^ *J*_HH_=12.8 Hz, ^3^ *J*_HH_=3.2 Hz, 1H; CH_2_ Cys), 1.09 (dd, ^3^ *J*_HP_=15.0 Hz, ^3^ *J*_HH_=7.4 Hz, 3H; CHC*H*_3_), 0.98 (ddd, ^2^ *J*_HH_=14.4 Hz, ^3^ *J*_HH_=10.2 Hz, ^4^ *J*_HP_=1.6 Hz, 1H; CH_2_ Cys), 0.77 (dd, ^3^ *J*_HP_=12.7 Hz, ^3^ *J*_HH_=6.8 Hz, 3H; CHC*H*_3_). ^13^C{^1^H} NMR (100.6 MHz, CD_3_OD, 298 K): δ=205.6 (dd, ^2^ *J*_CP_=20.4 Hz, ^2^ *J*_CP_=11.6 Hz; CO), 171.1 (s; Cys *C*OOH), 152.6–122.4 (m; aromatic carbon atoms), 57.2 (d, ^4^ *J*_CP_=2.8 Hz; Cys *C*H), 36.6 (dd, ^2^ *J*_CP_=5.0 Hz, ^2^ *J*_CP_=3.1 Hz; *C*H_2_), 33.8 (dd, ^1^ *J*_CP_=29.6 Hz, ^3^ *J*_CP_=2.5 Hz; P*C*H), 28.0 (s; Cys *C*H_2_), 22.7 (dd, ^1^ *J*_CP_=28.5, ^3^ *J*_CP_=1.8 Hz; P*C*H), 17.7 (d, ^2^ *J*_CP_=6.5 Hz; CH*C*H_3_), 16.3 (d, ^2^ *J*_CP_=1.6 Hz; CH*C*H_3_). ^31^P{^1^H} NMR (162 MHz, CD_3_OD, 298 K): δ 40.5 (d, ^2^ *J*_PP_=29.4 Hz), 28.9 ppm (d, ^2^ *J*_PP_=29.4 Hz), −144.5 (hept, ^1^ *J*_PF_=706.3 Hz).

**Synthesis of [Ru((*S*)**‐**Cys)(CO)((*S,S*)**‐**Skewphos)(phen)]PF_6_ (2** ^***S***^‐**Cys)**: Complex [Ru((*S*)‐Cys)(CO)((*S,S*)‐Skewphos)(phen)]PF_6_ (**2** ^***S***^‐**Cys**) was prepared following the procedure described for **2** ^***R***^‐**Cys**, starting from **2** ^***S***^ (50.0 mg, 0.058 mmol), in place of **2** ^***R***^, and (*S*)‐cysteine (7 mg, 0.058 mmol). Yield: 38 mg (65 %). Elemental analysis (%) calcd for C_45_H_44_F_6_N_3_O_3_P_3_RuS: C53.26, H 4.37, N 4.14; found: C 53.22, H 4.36, N 4.13. ^1^H NMR (400.1 MHz, CD_3_OD, 298 K): δ=8.64 (d, ^3^ *J*_HH_=8.1 Hz, 1H; phen), 8.60 (br m, 1H; phen), 8.47 (d, ^3^ *J*_HH_=8.0 Hz, 1H; phen), 8.22 (t, ^3^ *J*_HH_=8.6 Hz, 2H; Ph), 8.12 (d, ^3^ *J*_HH_=8.8 Hz, 1H; phen), 8.03 (d, ^3^ *J*_HH_=8.8 Hz, 1H; phen), 8.00–7.88 (m, 3H; Ph), 7.85 (t, ^3^ *J*_HH_=7.4 Hz, 2H; phen), 7.61–7.48 (m, 4H; aromatic protons), 7.45 (t, ^3^ *J*_HH_=7.5 Hz, 1H; Ph), 7.35 (dd, ^3^ *J*_HH_=8.1 Hz, ^4^ *J*_HH_=5.4 Hz, 1H; phen), 7.31–7.17 (m, 4H; Ph), 7.10–6.97 (m, 4H; Ph), 6.78 (t, ^3^ *J*_HH_=8.6 Hz, 2H; Ph), 3.50 (br m, 1H; PCH), 3.15 (m, 1H; PCH), 2.68 (m, 1H; CH_2_), 2.52 (dd, ^3^ *J*_HH_=9.2 Hz, ^3^ *J*_HH_=3.5 Hz, 1H; CH Cys), 2.39–2.11 (m, 1H; CH_2_), 1.54 (ddd, ^2^ *J*_HH_=12.2 Hz, ^3^ *J*_HH_=9.2 Hz, ^4^ *J*_HP_=2.4 Hz, 1H; CH_2_ Cys), 1.32 (dt, ^2^ *J*_HH_=12.9 Hz, ^3^ *J*_HH_=2.8 Hz, 1H; CH_2_ Cys), 1.09 (dd, ^3^ *J*_HP_=15.3 Hz, ^3^ *J*_HH_=7.3 Hz, 3H; CHC*H_3_*), 0.78 (dd, ^3^ *J*_HP_=12.7 Hz, ^3^ *J*_HH_=6.8 Hz, 3H; CHC*H_3_*). ^13^C{^1^H} NMR (100.6 MHz, CD_3_OD, 298 K): δ=205.4 (dd, ^2^ *J*_CP_=20.3 Hz, ^2^ *J*_CP_=11.6 Hz; CO), 170.5 (s; Cys *C*OOH), 152.7‐122.4 (m; aromatic carbon atoms), 56.8 (s; Cys *C*H), 36.6 (br t; *C*H_2_), 33.8 (d, ^1^ *J*_CP_=28.5 Hz; P*C*H), 27.7 (s; Cys *C*H_2_), 22.7 (dd, ^1^ *J*_CP_=28.5, ^3^ *J*_CP_=1.8 Hz; P*C*H), 17.7 (d, ^2^ *J*_CP_=6.4 Hz; CH*C*H_3_), 16.3 (s; CH*C*H_3_). ^31^P{^1^H} NMR (162 MHz, CD_3_OD, 298 K): δ 40.3 (d, ^2^ *J*_PP_=29.5 Hz), 29.0 ppm (d, ^2^ *J*_PP_=29.5 Hz), −144.5 (hept, ^1^ *J*_PF_=707.9 Hz).

**DFT calculations**: Density functional theory (DFT) calculations have been performed to: i) determine the relative stability of [Ru(η^1^‐OAc)(CO)((*R*,*R*)‐Skewphos)(phen)]^+^ isomers; ii) study the hydrolysis reactions of **2** ^***R+***^ and the reactions of its derivatives with cysteine; iii) energy stability of **2** ^***R+***^ complex with (*S*)‐ and (*R*)‐cysteine. All calculations were performed only on the complex containing the (*R*,*R*)‐Skewphos ligand. As reported experimentally, the (*S*,*S*)‐Skewphos complex showed a reactivity quite similar to that observed for (*R*,*R*), thus only the latter was considered in the calculations. All DFT calculations were performed using the B3LYP functional, composed of Becke's three‐parameter hybrid exchange functional (B3)^[58]^ and the correlation functional of Lee, Yang, and Parr (LYP).^[59]^ Additionally, Grimme's dispersion contribution correction (D3) was added,^[60]^ as previous works have shown that B3LYP−D3 scheme provides reliable results for thermochemistry of organometallic compounds.[^61^, ^62^, ^63^, ^64^, ^65^] The def2SVP basis set, including the ECP for Ru, was employed.[^66^, ^67^] Due to the key role of solvation in influencing thermodynamic and kinetic parameters, solvent effects were introduced by the polarizable continuum method (PCM).^[68]^ Geometry optimizations have been run in methanol, free energies have been by adding the zero‐point energy and thermal correction terms to the electronic energy of the complex. The nature of stationary points (minima or transition states) has been checked by vibrational analysis. The hydrolysis reaction of **2** ^***R+***^ complex is key step in the activation of this species,[^46^, ^47^, ^48^, ^69^] which has been studied with the same computational protocol reported above using water as solvent. In a similar way, the reactions of **2** ^***R+***^ and its aquo‐derivate with (*S*)‐cysteine were studied in different conditions: acidic‐neutral (pH 4–7), where the zwitterionic form of (*S*)‐cysteine prevails, while at pH 9.0–9.5 about 60 % is in the anionic form (deprotonation of S atom).^[50]^ All calculations were performed in using Gaussian 16 program.^[70]^ The analysis of intermolecular and intramolecular interactions, previously applied in the analysis of non‐covalent interactions for Pt‐anticancer compounds,^[71]^ was performed by means of the Independent Gradient Model (IGM) method[^44^, ^45^] employing the IGMPlot software version 2.6.7. This analysis allows to calculate a descriptor, δg, which represents the difference between a virtual upper limit of the electron density gradient for a non‐interacting system (|∇ρIGM|) and the actual electron density gradient (|∇ρ|). In this analysis, the term associated to all interactions can be separated in an intermolecular (δg^inter^=|∇ρIGM,^inter^|–|∇ρ|) and intramolecular (δg^intra^=|∇ρIGM|–|∇ρIGM,^inter^| part (with δg=δg^inter^+δg^intra^).

**Materials for biological testing**: DMEM (Dulbecco's Modified Eagle Medium) w/GlutaMAX^TM^‐I (pyruvate 1 mM) cell growth medium was purchased from Thermo Fisher Life Technologies while fetal bovine serum, sterile DMSO, *cis*‐diammineplatinum(II) dichloride (hereinafter, cisplatin) and 3‐(4,5‐dimethyl‐2‐thiazolyl)‐2,5‐diphenyl‐2H‐tetrazolium bromide (MTT) were from Merck. Penicillin‐streptomycin (solution; 5000 U/mL) were acquired from Thermo Fisher Life Technologies. FITC Annexin V apoptosis detection kit I was purchased from Thermo Fisher Scientific, (Waltham, MA, USA). All chemicals were of high‐grade pureness and used as purchased without any further purification.

**Solution stability evaluation through a 72**‐**h scanning kinetics in different physiological media**: Given the span of the in vitro tests subsequentially carried out (72 h), several UV‐Vis spectra were automatically acquired at regular time intervals at room temperature. Based on the enantiomeric relationship between the three couples of studied complexes, first the (*S*,*S*)‐enantiomers were tested, as model compounds, in not chiral media (DMSO, deionized water, saline solution (0.9 % NaCl w/v) and PBS). The analysis started with the preparation of a 1 mM DMSO solution, followed by the acquisition of the spectra over time at a compound final concentration ranging from 20 to 50 μM (the latter allowed to check the behavior of the band at about 370 nm; DMSO content=4 % v/v). All the scanning kinetics in DMSO, water, saline and PBS are reported in Supporting Information (Figures S38‐S49).

Finally, the stability in the complete cell culture medium DMEM (Dulbecco's Modified Eagle Medium), supplemented with 10 % fetal bovine serum, was investigated for each enantiomer of the three pairs (compound concentration=40 μM; 25/75 % v/v DMEM/PBS). The scanning kinetics in DMEM cell culture medium are reported in Supporting Information (Figures S50–S53).

**Cell culture and cell viability assay**: Human colon carcinoma HCT‐116 cells and non‐small cell lung carcinoma A549 cells were obtained from American Type Culture Collection (Manassas, VA) and grown in DMEM supplemented with 10 % fetal bovine serum. SW1736 and 8505 C cells, derived from ATC, were cultured in RPMI‐1640 medium (Euroclone S.p.A) supplemented with 10 % FBS (Gibco; Thermo Fisher Scientific, Inc.), 2 mM L‐glutamine (Euroclone S.p.A) and 50 mg/mL gentamicin (Gibco; Thermo Fisher Scientific, Inc.). The cell lines were validated using short tandem repeat analysis and confirmed to be mycoplasma‐free.

Cells were grown in a humidified incubator with 5 % CO_2_ at 37 °C. Cells were seeded in 96‐well plates (volume=100 μL; 4000 cells/well for SW1736 and 8505 C cell lines; 5500 cells/well and 7000 cells/well for HCT‐116 and A549, respectively) and grown to 70–75 % confluence, followed by treatment with DMSO (control) or each chiral compound (dissolved in DMSO) in fresh medium at different concentrations in the micromolar or sub‐micromolar domain (both in control and in treatment wells a final DMSO concentration of 0.1 % v/v; quadruplicate conditions). Likewise, cells were seeded in quadruplicate in 96‐well plates and grown to the same confluence to be treated with cisplatin (dissolved in 0.9 % w/v NaCl(aq)) in fresh medium at different concentrations, for comparison purposes.

After 72‐h incubation at 37 °C, inhibition of cell proliferation was measured by MTT assay, as previously described.^[72]^ The cytotoxicity of the compounds was quantified as the percentage of surviving cells compared to untreated cells. At least three MTT tests for each compound were carried out in order to evaluate the corresponding EC_50_ values.

**Soft agar assay**: The clonogenic ability of the SW1736 and 8505C cells after treatment with chiral ruthenium complexes each used at its own EC_50_ was evaluated using a soft agar assay. Briefly, 48 h after treatment, cells were collected, and 1×10^4^ cells were suspended in 4 mL complete medium containing 0.25 % agarose (Sigma‐Aldrich), then seeded to the top of a 1 % agarose complete medium layer in 6‐cm plates. The colonies were counted by eye in four different fields, under a Leica DMI‐600B inverted microscope (Leica Microsystems Ltd.). Data are representative of three independent experiments.

**Apoptosis assay**: Apoptosis indexes were measured using the Annexin‐V fluorescein isothiocyanate (FITC) apoptosis detection kit I from by Thermo Fisher Scientific, (Waltham, MA, USA). HCT‐116 cells were grown to approximately 75 % confluence, treated with the most promising chiral complexes **2** ^***R***^ and **4** ^***R***^ (at 0.5 μM) or DMSO vehicle as a control (0.1 % v/v) for 72 h, harvested by trypsinization and centrifugation. Cells were rinsed twice with ice cold PBS (1X) and re‐suspended in binding buffer (1X) at a concentration of 3x10^6^ cells/mL. The suspension (200 μL) was then transferred to a 5 mL flow cytometry tube. Cells were incubated with 5 μL of annexin V‐FITC for 10 minutes in the dark. Propidium iodide (PI; 10 μL) was added in each tube just before the acquisition of the sample on the flow cytometry instrument. For annexin V/PI assay analysis, approximately 1.0×10^4^‐gated events were acquired for each sample by a FACSCanto flow cytometer (Becton Dickinson). Flow cytometry data were processed using FlowJo software (v10 TreeStar). The excitation wavelength was 488 nm and the detection wavelengths were 530±15 and 620±21 nm for Annexin V and PI, respectively.

Data (Figure 11) are shown as density plots of Annexin‐V (x‐axis) and propidium iodide (PI, y‐axis) staining. Viable cells were defined as annexin V‐negative and PI‐negative. Early apoptotic cells were defined as annexin V‐positive and PI‐negative, late apoptotic cells were defined as annexin V‐ and PI‐positive whereas cells positive for PI only were considered dead by necrosis. Percentages of viable, apoptotic, and necrotic cells are reported in the corner of each quadrant.

**Cellular morphology and cell migration assay**: An Olympus IX70 inverted tissue culture microscope was used for evaluating cellular morphology changes upon treatment and microscopic imaging with phase contrast. Cell migration was assessed using the scratch wound healing assay, as described elsewhere.^[73]^ Cells were grown to confluence in tissue culture dishes, then the very promising compound **2** ^***R***^ (final concentration=3 μM) or drug‐free medium were added. After 24 h, cells were rinsed twice with PBS and scraped up using a sterile 1,000 μL pipette tip, then cultured in the abovementioned medium. The migration rate is associated with change of the distance between the edges of the wound (defined by the lines), indicating the cell‐free surface area. Pictures here reported are representative of one of three different experiments (original magnification 4X; scale bar=100 μm).

**Log** ***P*** **evaluation**: When evaluating the partition coefficient *P*, *n*‐octanol was pre‐saturated with milli‐Q water for 24 h under vigorous stirring, followed by equilibration at 25 °C for 24 h. After that, weighted amounts of the (*S*,*S*)‐enantiomers were dissolved in a defined volume of the organic phase (final concentration in the 50–100 μM range), then evaluated their actual concentration by measuring the absorbance at the maximum wavelength of the electronic band at about 270 nm. The solution was mixed with water and let to stir for 2 h at 25 °C. Later, the mixture was left to equilibrate for at least 30 min. The concentration of every complex in the organic phase before (C_0_) and after partitioning (C_1_) was evaluated by UV‐Vis spectrophotometry, resulting in the calculation of the corresponding *n*‐octanol/water partition coefficient (*P*) as log *P*=log (C_1_)/(C_0_–C_1_).^[74]^ The procedure was repeated at least three times for each compound.

**Statistical analysis**: Data are presented as the mean±standard deviation. All results were analyzed using the unpaired Student's t‐test or one‐way ANOVA in GraphPAD Prism version 6 (GraphPAD Software, Inc.). After one‐way ANOVA, the Dunnett's post hoc test was performed. P<0.05 was considered to indicate a statistically significant difference.

## Supporting Information

NMR data, DFT calculated thermochemical data and structures, solution stability data, electronic spectra of free ligands, microscope images.

## Conflict of interest

The authors declare no conflict of interest.

1

## Supporting information

As a service to our authors and readers, this journal provides supporting information supplied by the authors. Such materials are peer reviewed and may be re‐organized for online delivery, but are not copy‐edited or typeset. Technical support issues arising from supporting information (other than missing files) should be addressed to the authors.

Supporting Information

## Data Availability

The data that support the findings of this study are available in the supplementary material of this article.
