# Supplementary material for: Enantioselective Cytotoxicity of Chiral Diphosphine Ruthenium(II) Complexes Against Cancer Cells
Source: Chemistry. 2022 May 5;28(33):e202200200. doi: 10.1002/chem.202200200 (PMC9322675; doi:10.1002/chem.202200200)
Supplement: Supplementary file 1 — Supporting Information [file CHEM-28-0-s001.pdf]

Chemistry–A European Journal

Supporting Information

Enantioselective Cytotoxicity of Chiral Diphosphine Ruthenium(II) Complexes Against Cancer Cells

Denise Lovison, Dario Alessi, Lorenzo Allegri, Federica Baldan, Maurizio Ballico, Giuseppe Damante,* Marilisa Galasso, Daniele Guardavaccaro, Silvia Ruggieri, Andrea Melchior, Daniele Veclani, Chiara Nardon,* and Walter Baratta*

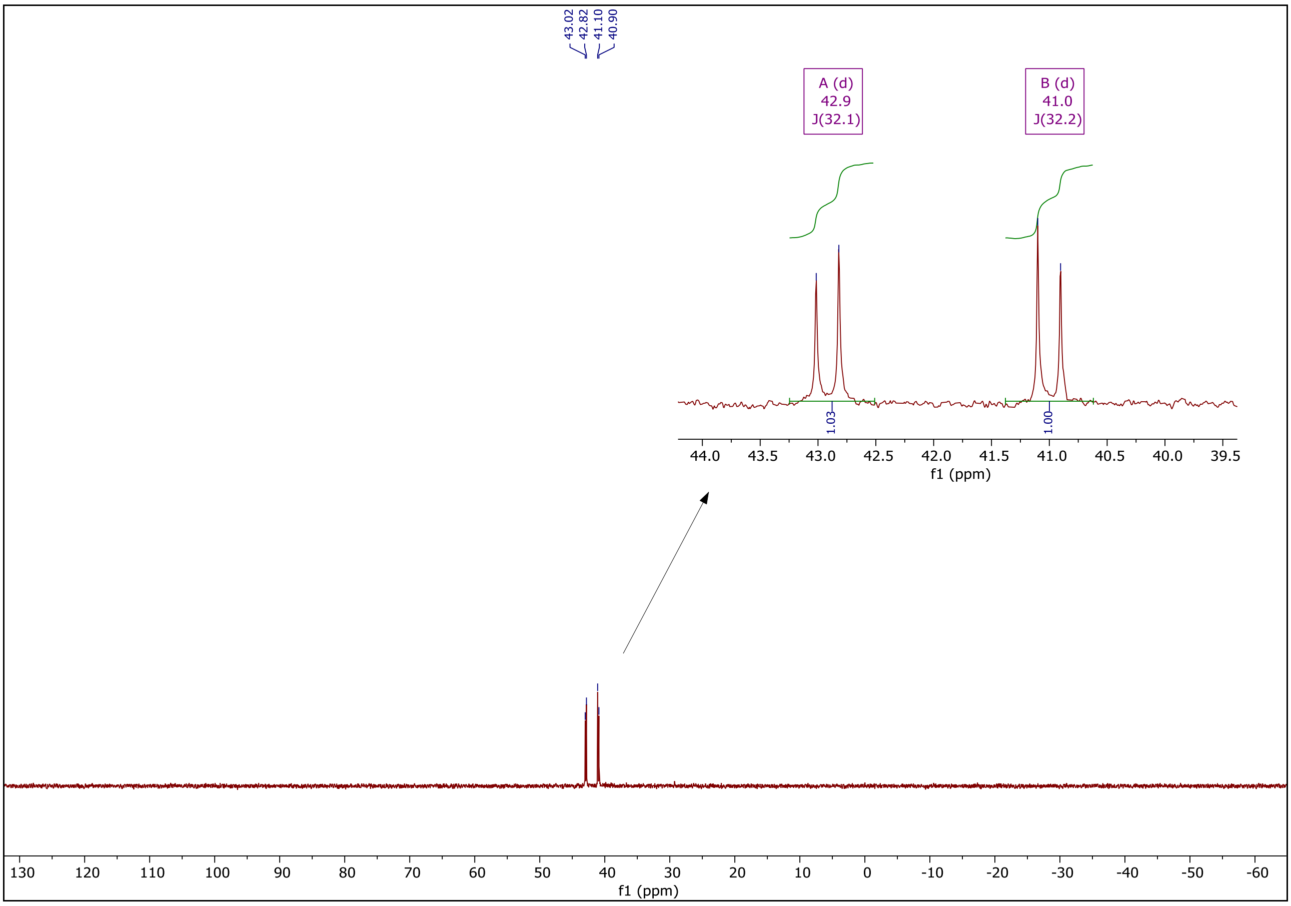

Figure S1. $^{31}P\{^1H\}$ NMR spectrum (162 MHz) of [Ru(η^1-OAc)(CO)((R,R)-Skewphos)(phen)]OAc (**2**$^{\boldsymbol{R}}$) in $CDCl_3$ at 298 K.

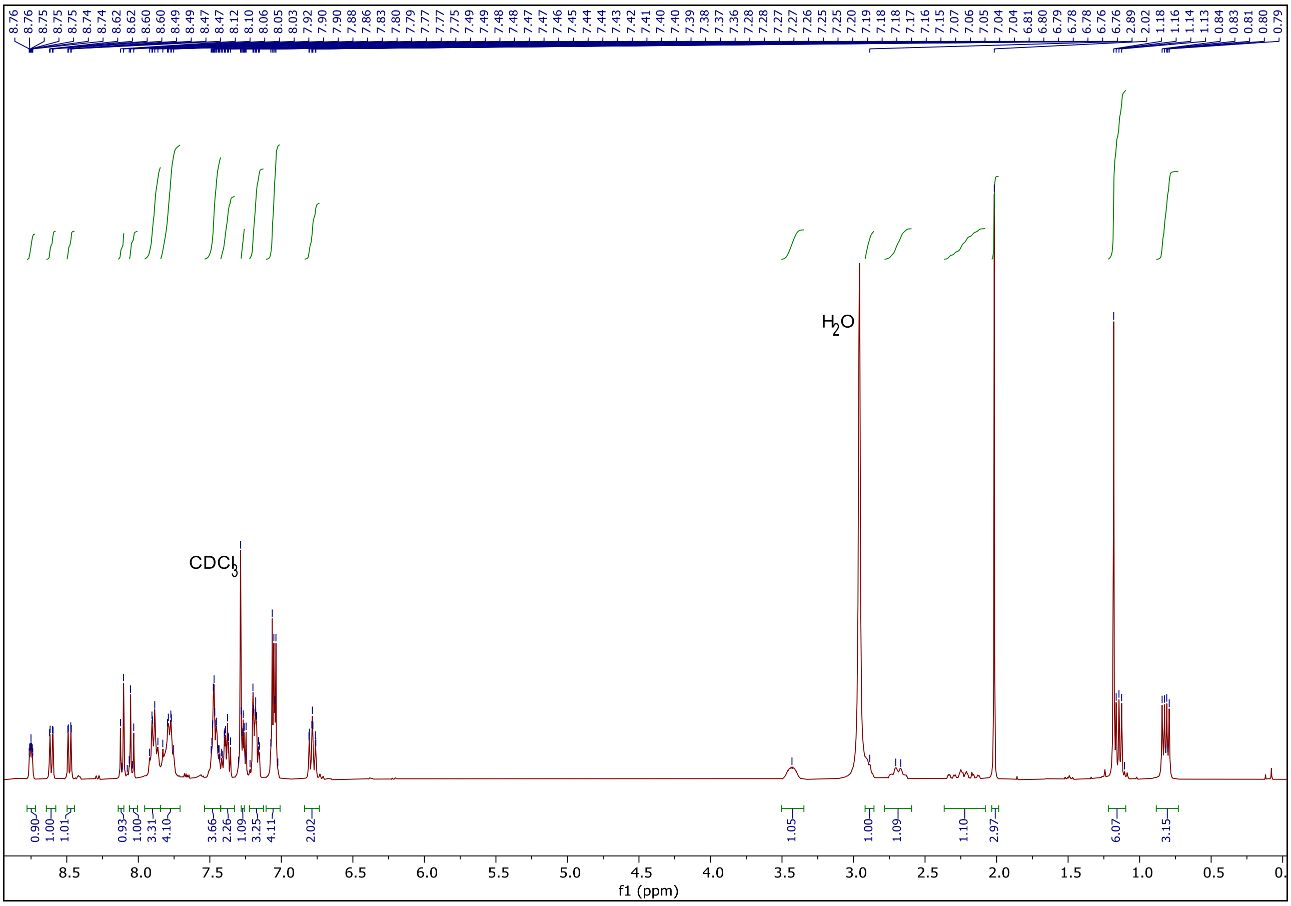

Figure S2. ^{1}H NMR spectrum (400.1 MHz) of [Ru(η^{1}-OAc)(CO)((R,R)-Skewphos)(phen)]OAc (**2*R***) in $CDCl_3$ at 298 K.

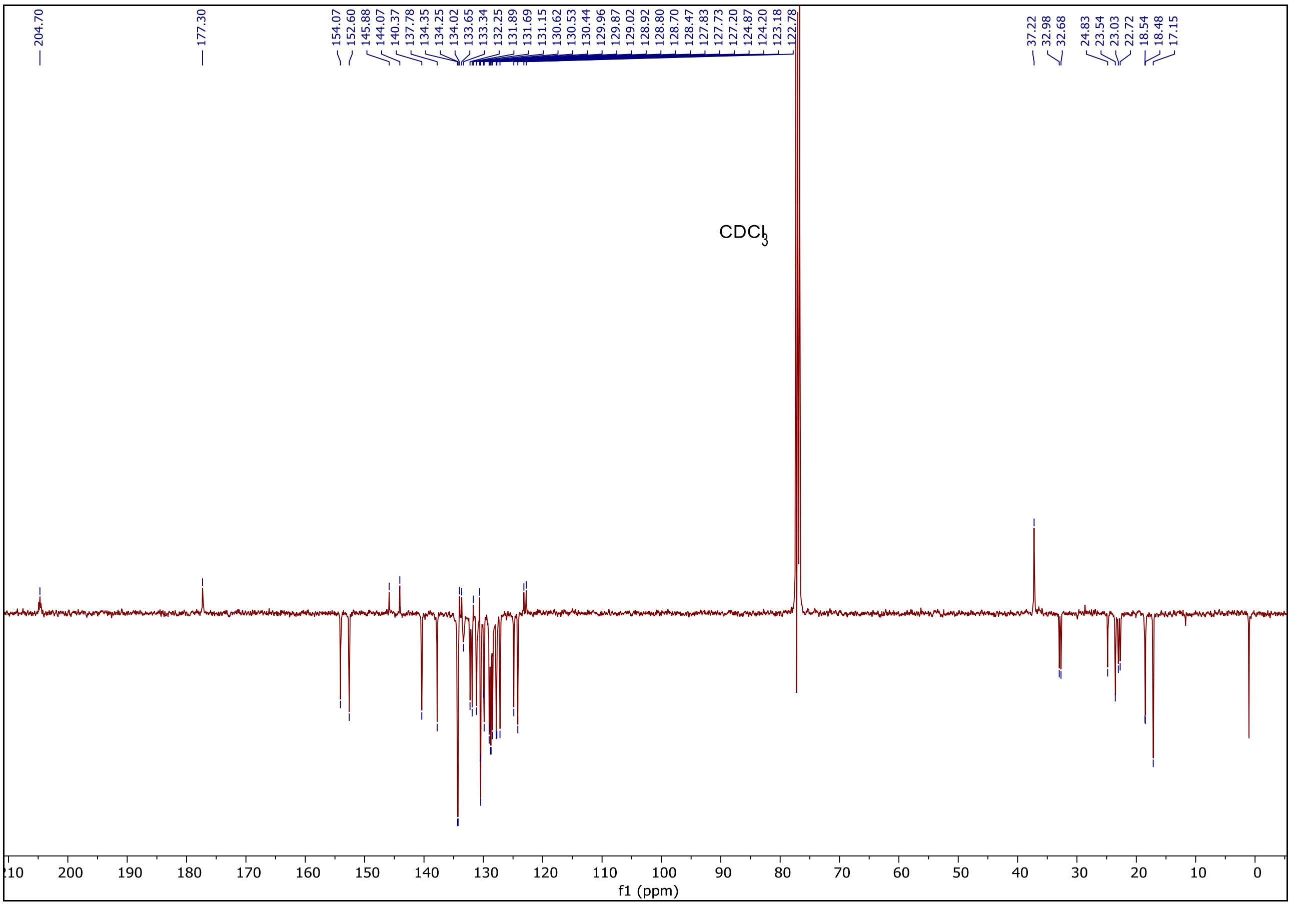

Figure S3. $^{13}C\{^{1}H\}$ DEPTQ NMR spectrum (100.6 MHz) of [Ru(η^1-OAc)(CO)((R,R)-Skewphos)(phen)] OAc (**2*^R^***) in $CDCl_3$ at 298 K.

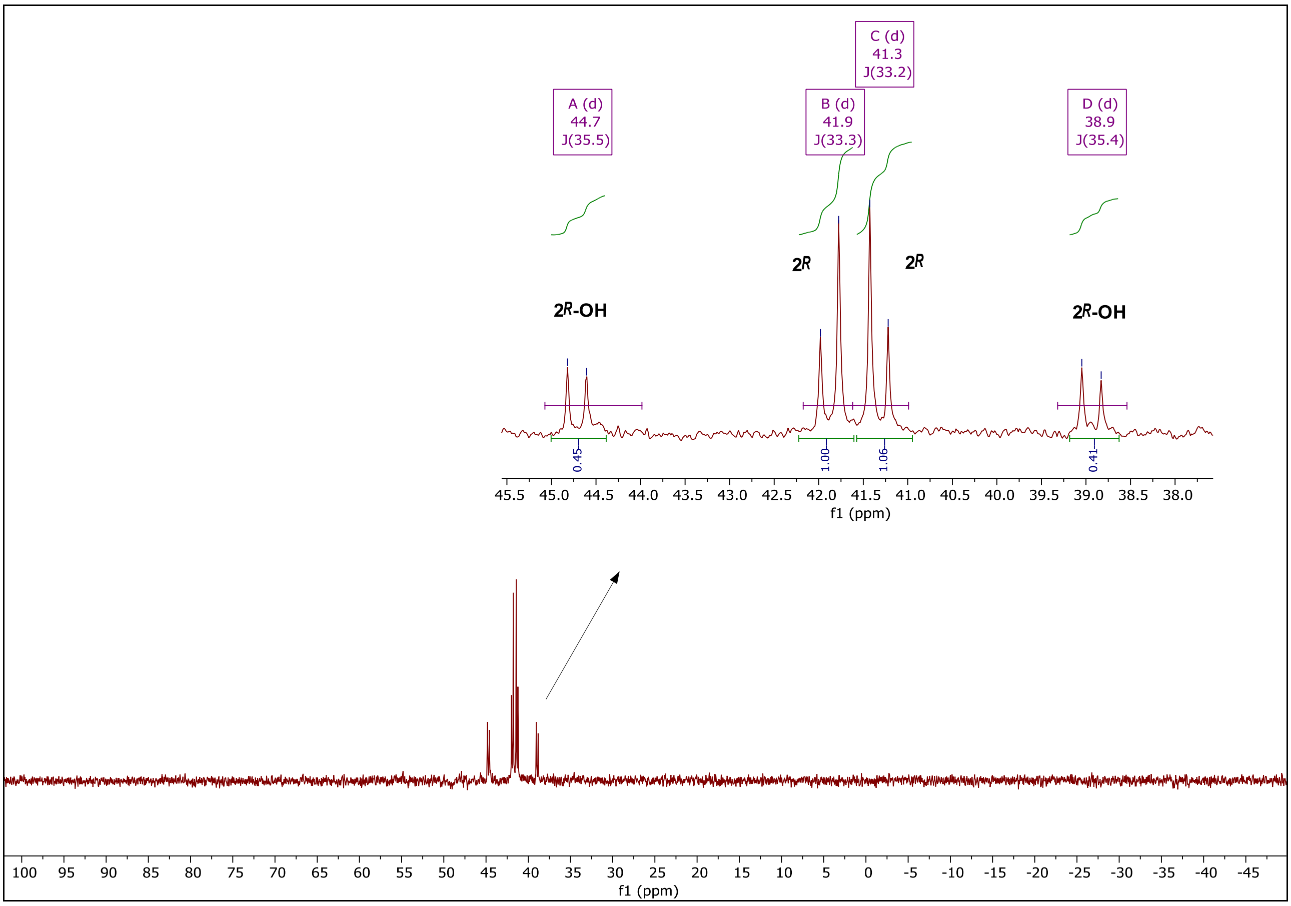

Figure S4. $^{31}P\{^1H\}$ NMR spectrum (162 MHz) of [Ru(η^1-OAc)(CO)((R,R)-Skewphos)(phen)] OAc (**2**$^{\boldsymbol{R}}$) in D_2O at 298 K.

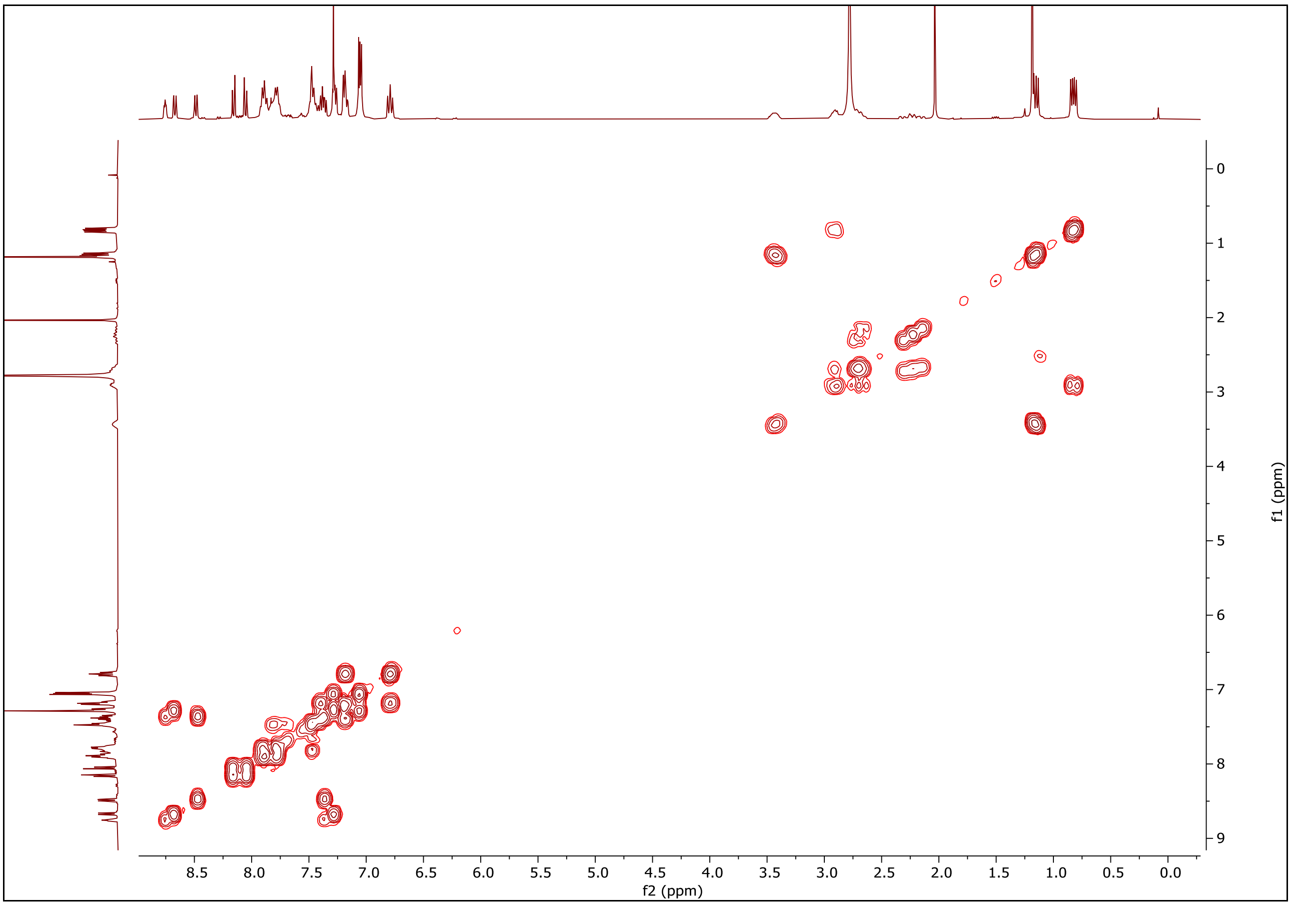

Figure S5. ^1H-^1H COSY NMR spectrum (400.1 MHz) of [Ru(η^1-OAc)(CO)((R,R)-Skewphos)(phen)]OAc (**2**$^{\boldsymbol{R}}$) in $CDCl_3$ at 298 K.

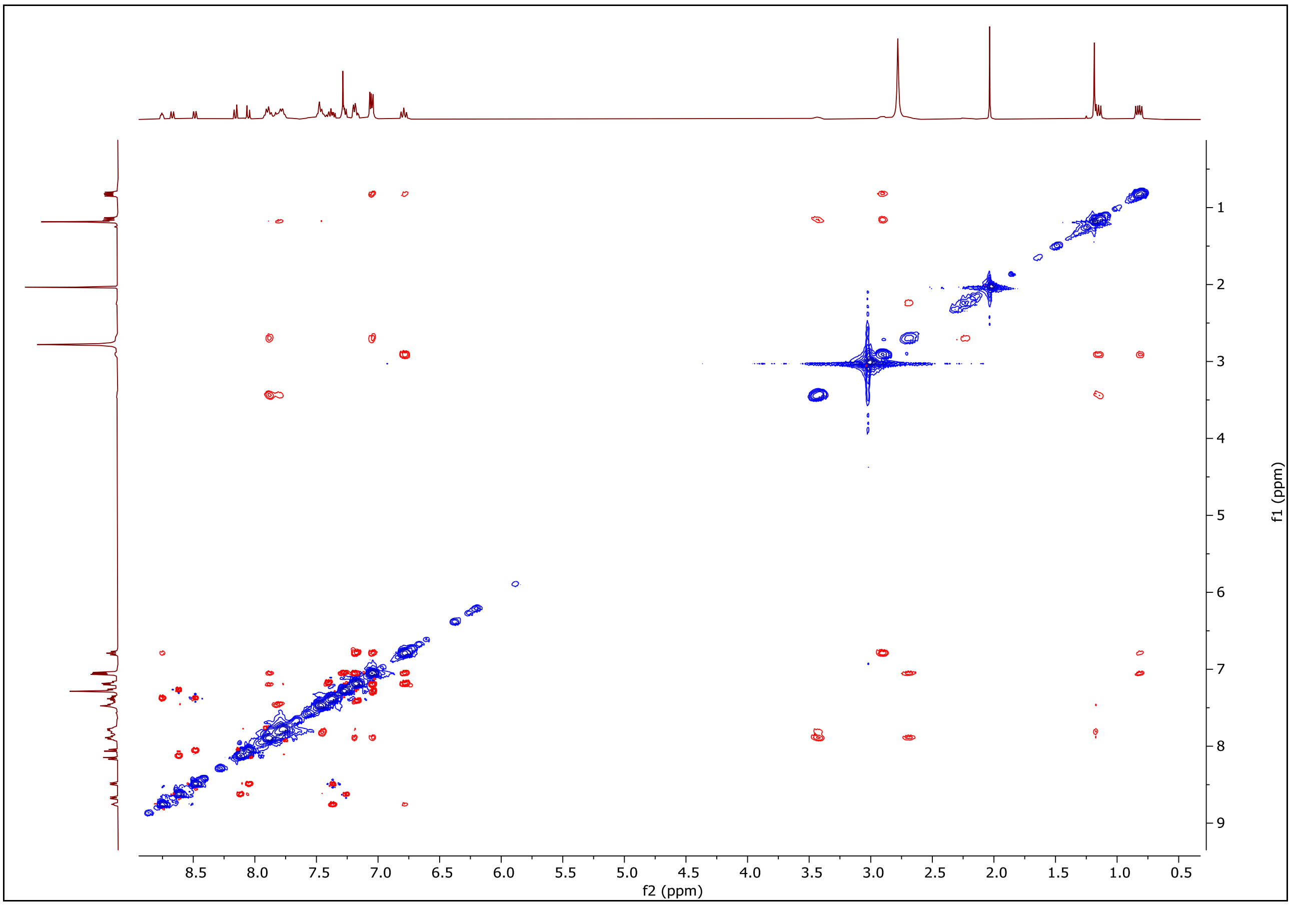

Figure S6. ^{1}H-^{1}H NOESY NMR spectrum (400.1 MHz) of [Ru(η[1]-OAc)(CO)((R,R)-Skewphos)(phen)]OAc (**2**[*R*]) in $CDCl_3$ at 298 K.

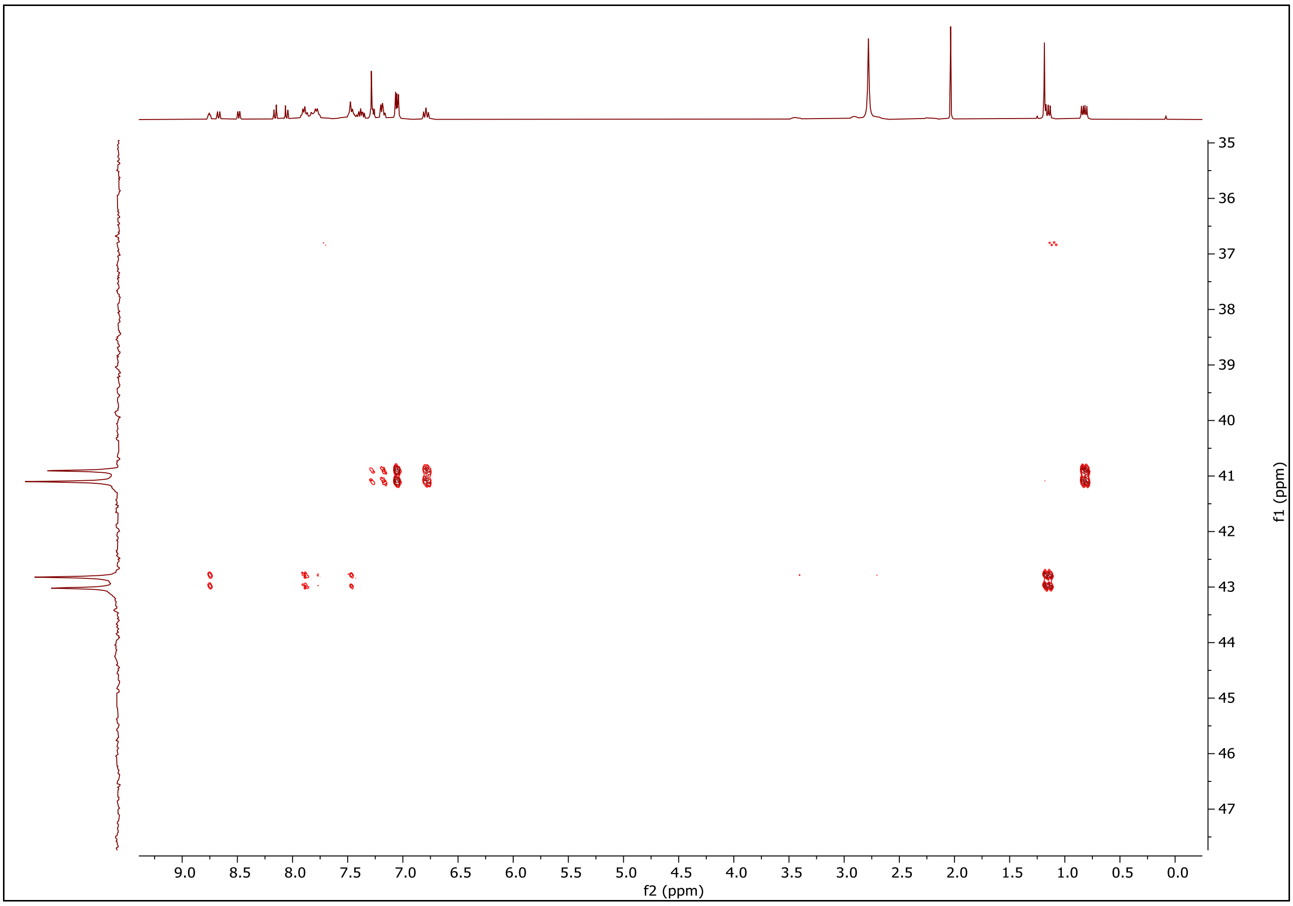

Figure S7. ^{31}P-^{1}H HMBC NMR spectrum of [Ru(η^{1}-OAc)(CO)((R,R)-Skewphos)(phen)]OAc (**2*R***) in $CDCl_3$ at 298 K.

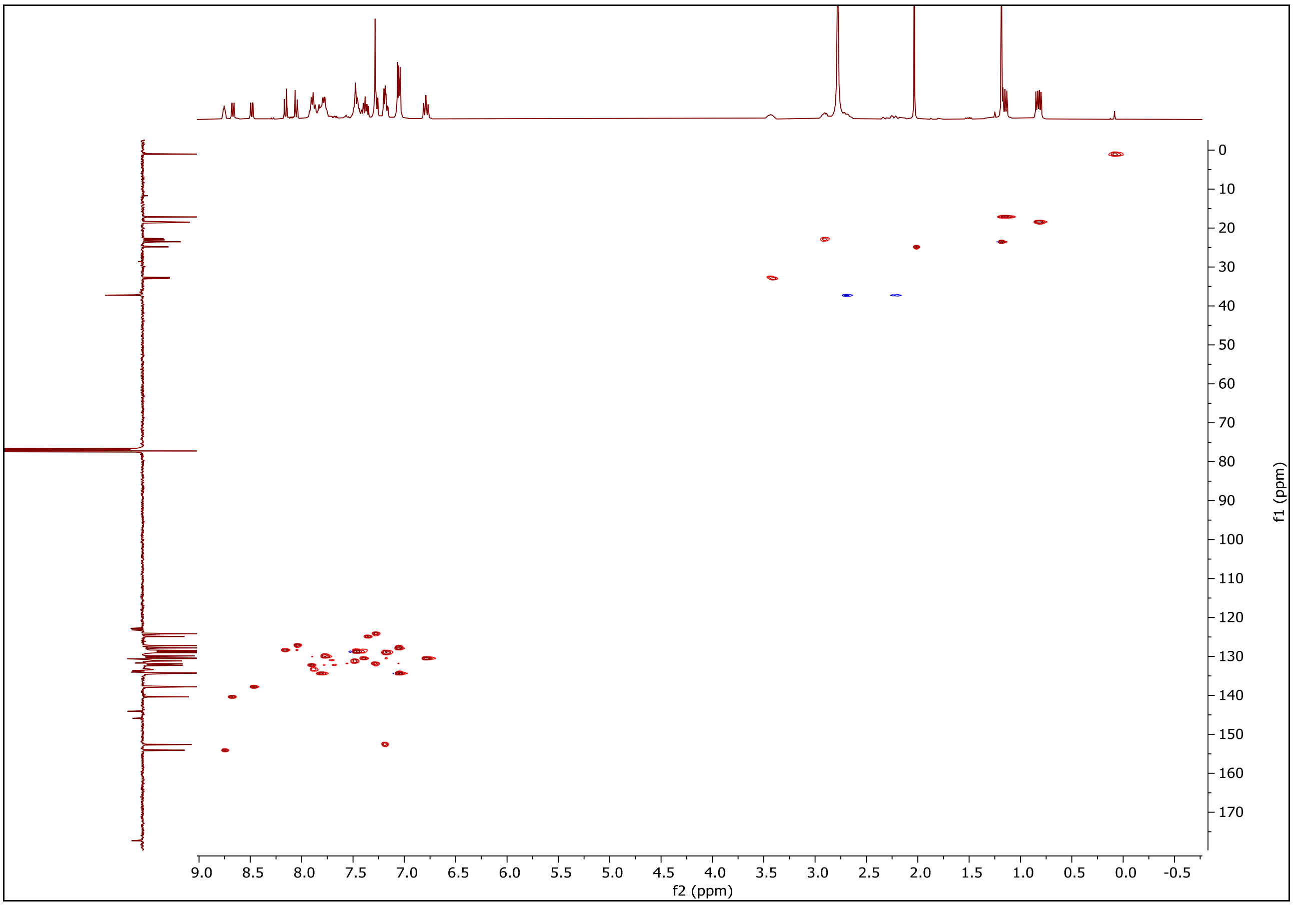

Figure S8. ^{13}C-^{1}H HSQC NMR spectrum of [Ru(η^1-OAc)(CO)((R,R)-Skewphos)(phen)]OAc (**2**$^{\boldsymbol{R}}$) in $CDCl_3$ at 298 K.

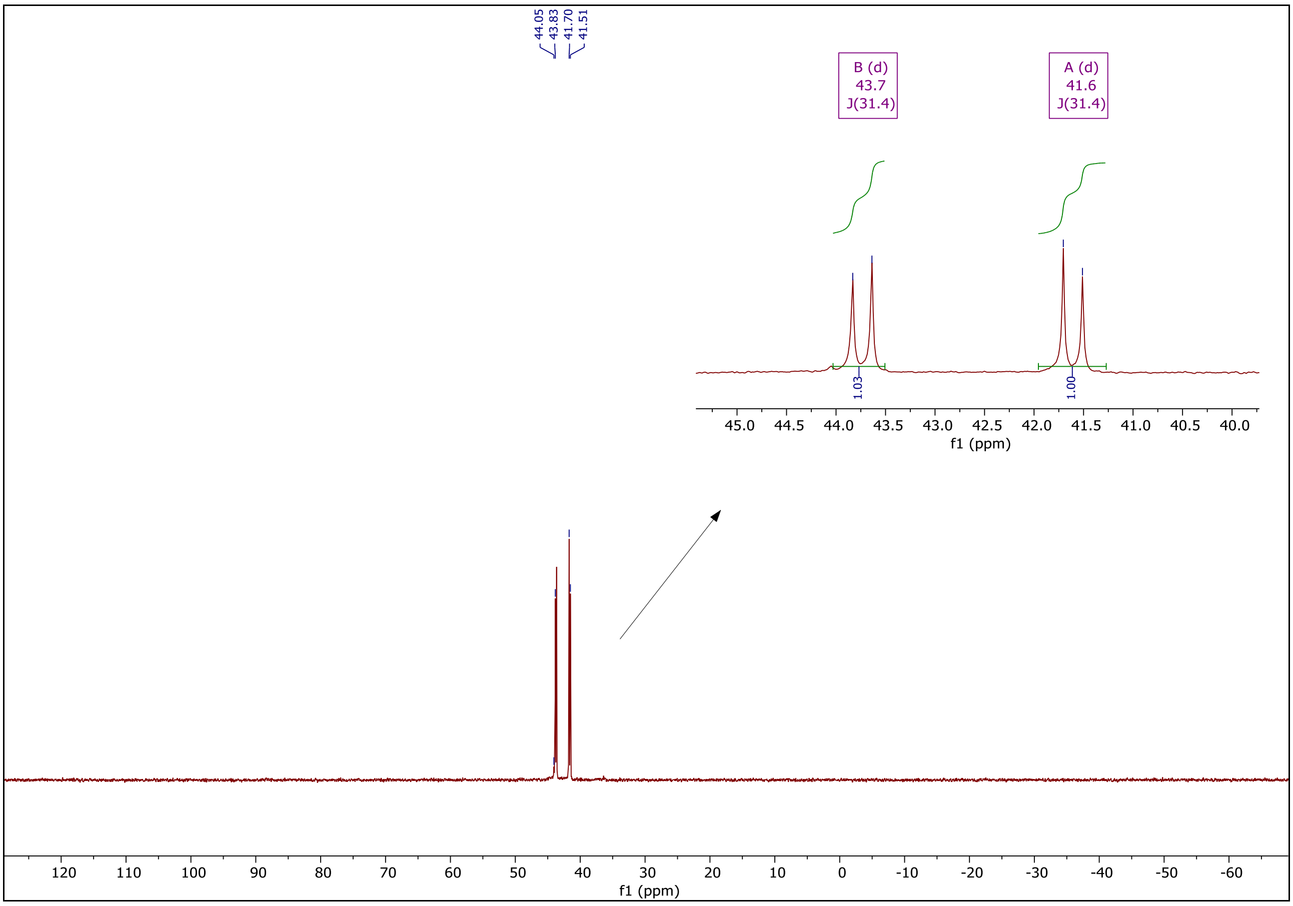

Figure S9. $^{31}P\{^{1}H\}$ NMR spectrum (162 MHz) of [Ru(η^1-OPiv)(CO)((R,R)-Skewphos)(phen)]OPiv (**3*R***) in $CDCl_3$ at 298 K.

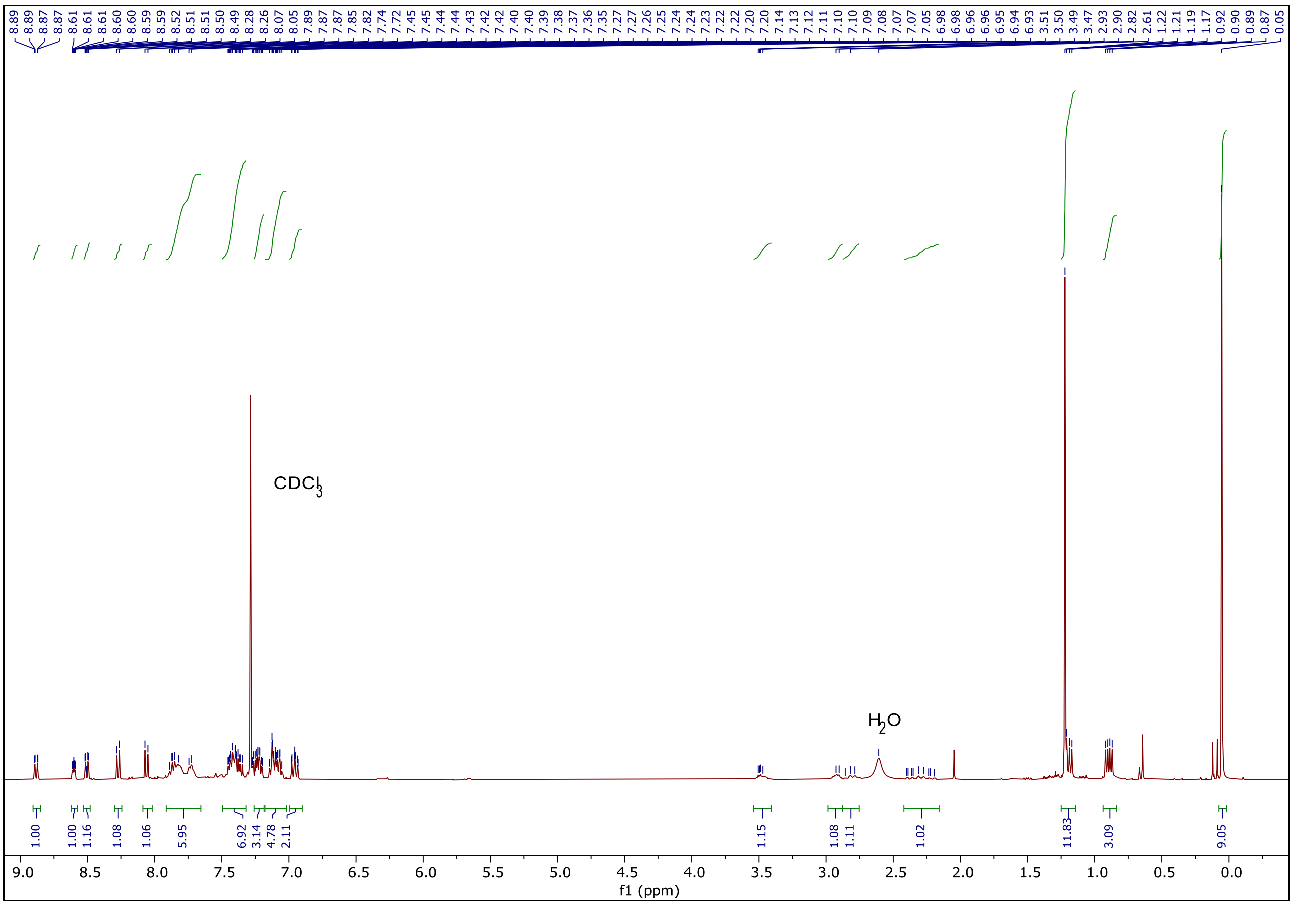

Figure S10. ^{1}H NMR spectrum (400.1 MHz) of [Ru(η^1-OPiv)(CO)((R,R)-Skewphos)(phen)]OPiv ($\mathbf{3}^{\boldsymbol{R}}$) in $CDCl_3$ at 298 K.

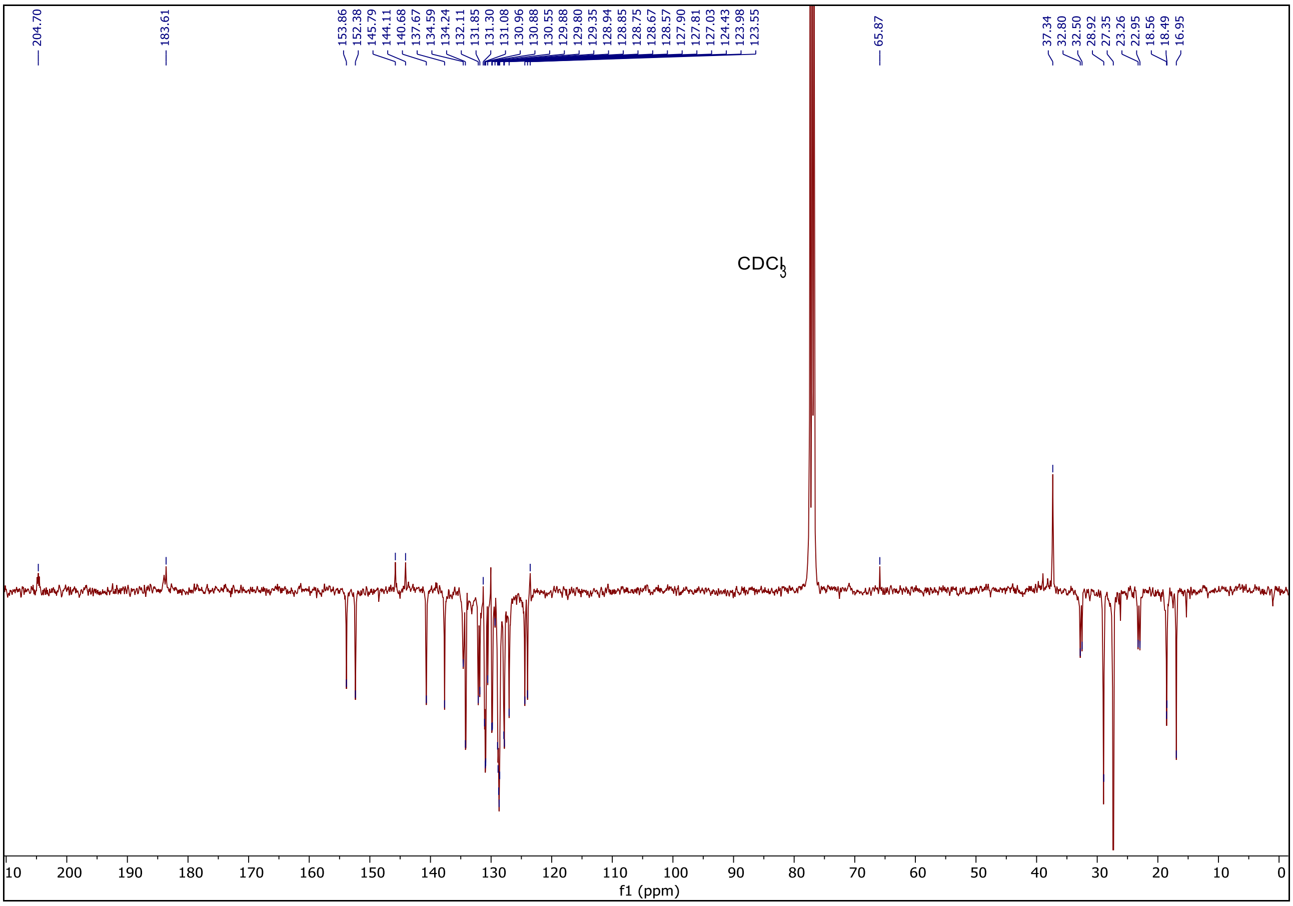

Figure S11. $^{13}C\{^{1}H\}$ DEPTQ NMR spectrum (100.6 MHz) of [Ru(η^{1}-OPiv)(CO)((R,R)-Skewphos)(phen)]OPiv (**3*^R^***) in $CDCl_3$ at 298 K.

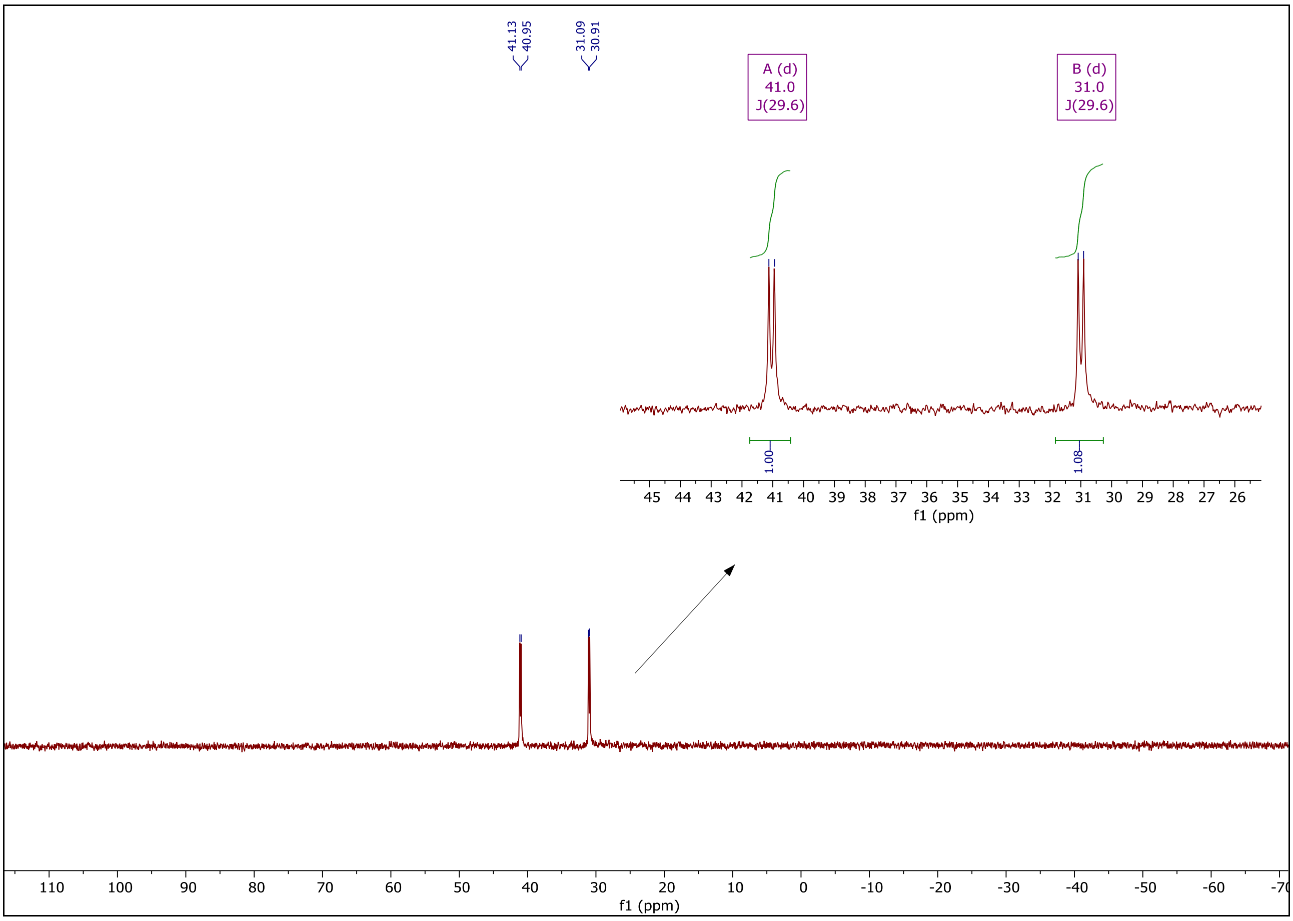

Figure S12. $^{31}P\{^{1}H\}$ NMR spectrum (162 MHz) of [Ru(η^{1}-SAc)(CO)((R,R)-Skewphos)(phen)]OAc (**4*^R^***) in $CDCl_3$ at 298 K.

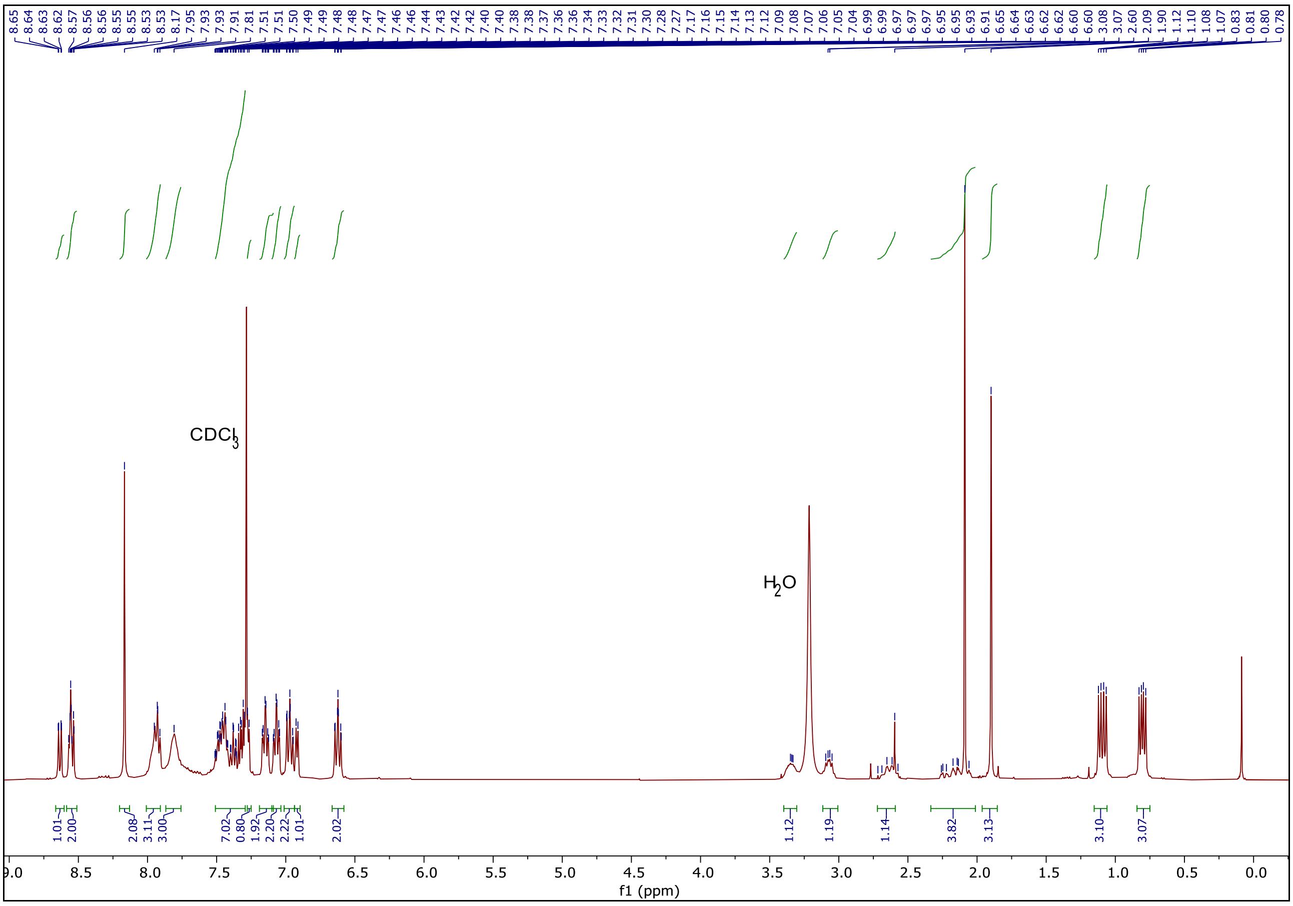

Figure S13. ^{1}H NMR spectrum (400.1 MHz) of [Ru(η^1-SAc)(CO)((R,R)-Skewphos)(phen)]OAc (**4*R***) in $CDCl_3$ at 298 K.

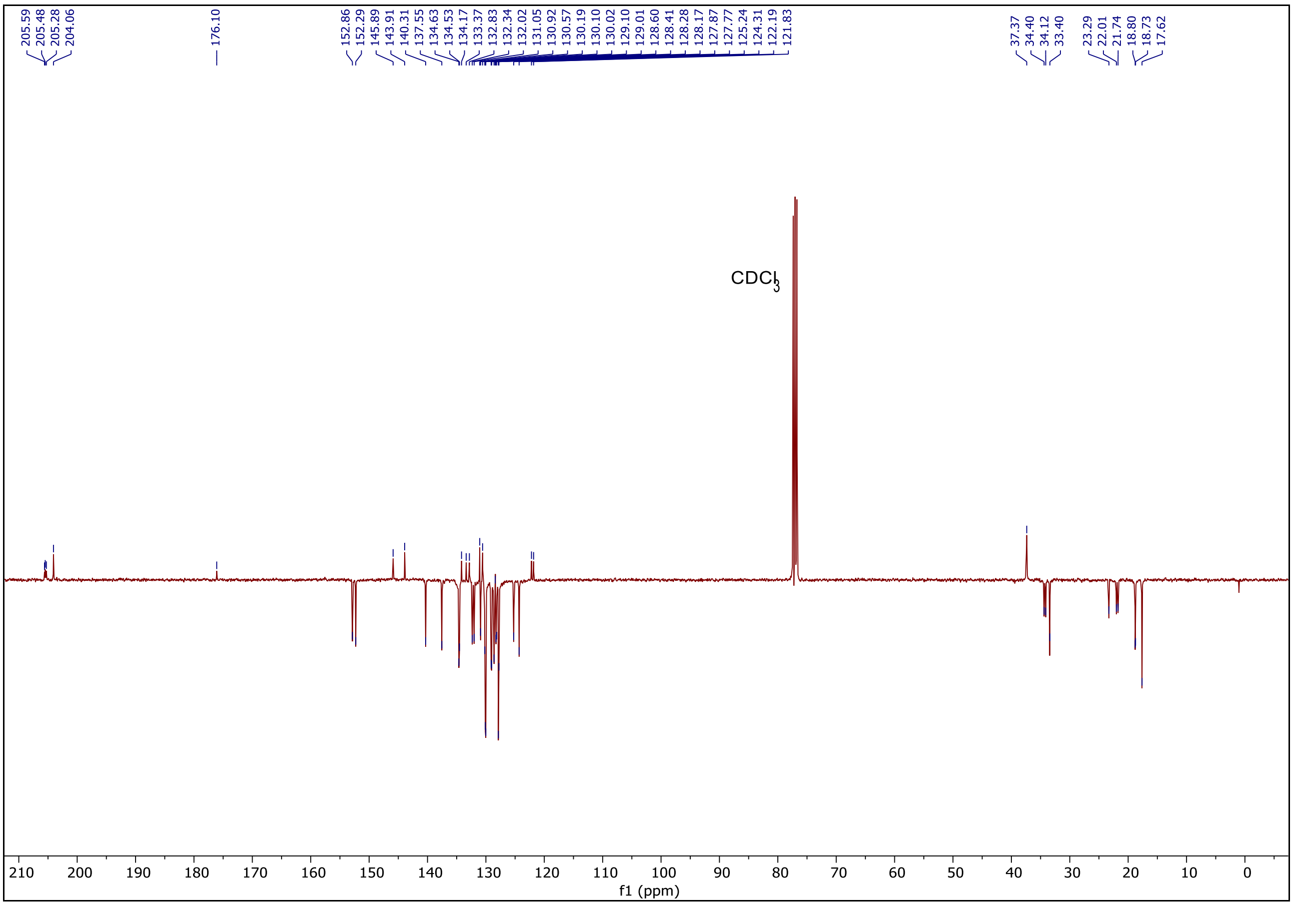

Figure S14. $^{13}C\{^1H\}$ DEPTQ NMR spectrum (100.6 MHz) of [Ru(η^1-SAc)(CO)((R,R)-Skewphos)(phen)] OAc (**4*R***) in $CDCl_3$ at 298 K.

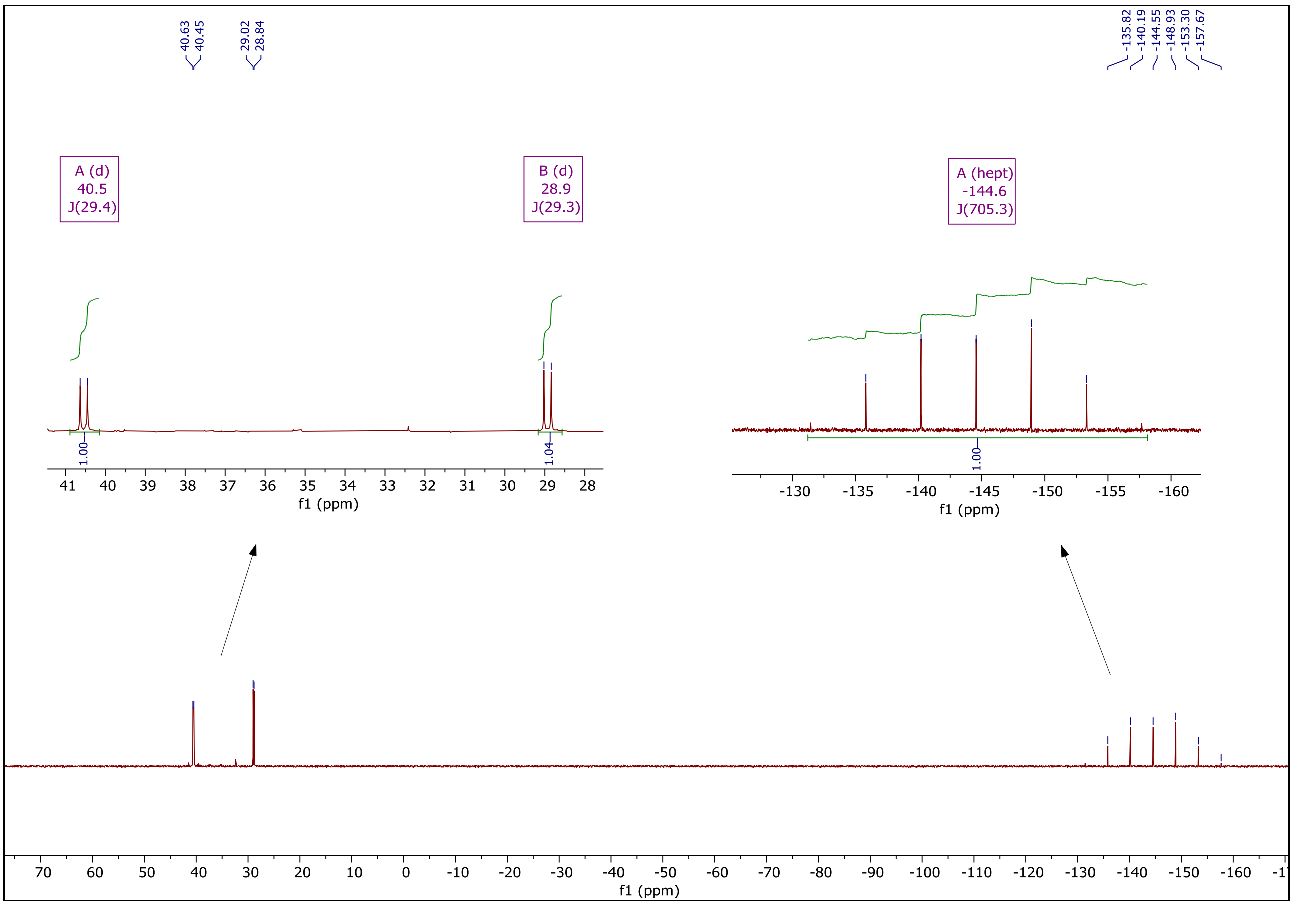

Figure S15. $^{31}P\{^1H\}$ NMR spectrum (162 MHz) of [Ru(L-Cys)(CO)((*R*,*R*)-Skewphos)(phen)]PF_6 (**2**$^{\boldsymbol{R}}$**-Cys**) in CD_3OD at 298 K.

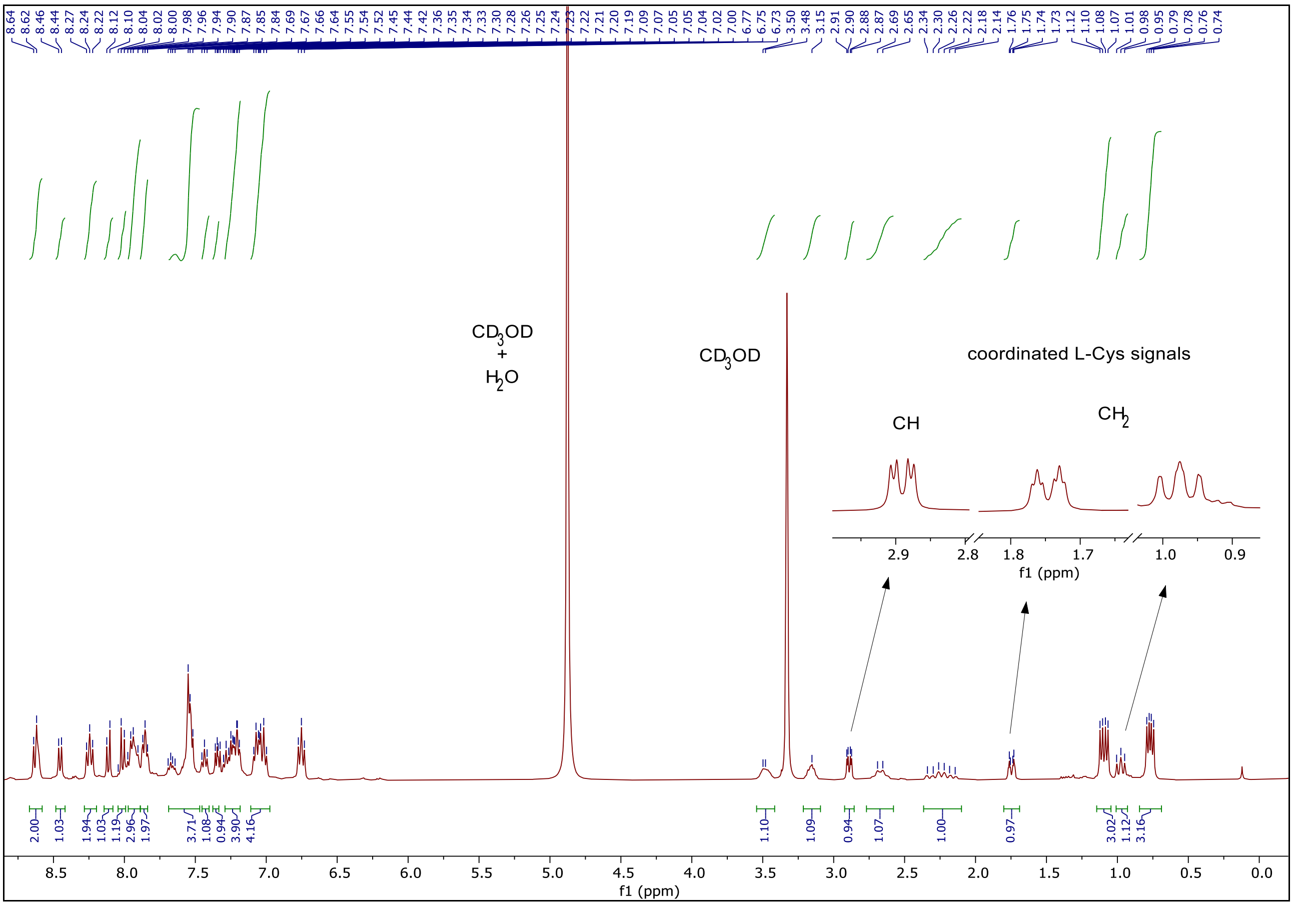

Figure S16. ^{1}H NMR spectrum (400.1 MHz) of [Ru(L-Cys)(CO)((*R*,*R*)-Skewphos)(phen)]PF_6 (**2**$^{\boldsymbol{R}}$**-Cys**) in CD_3OD at 298 K.

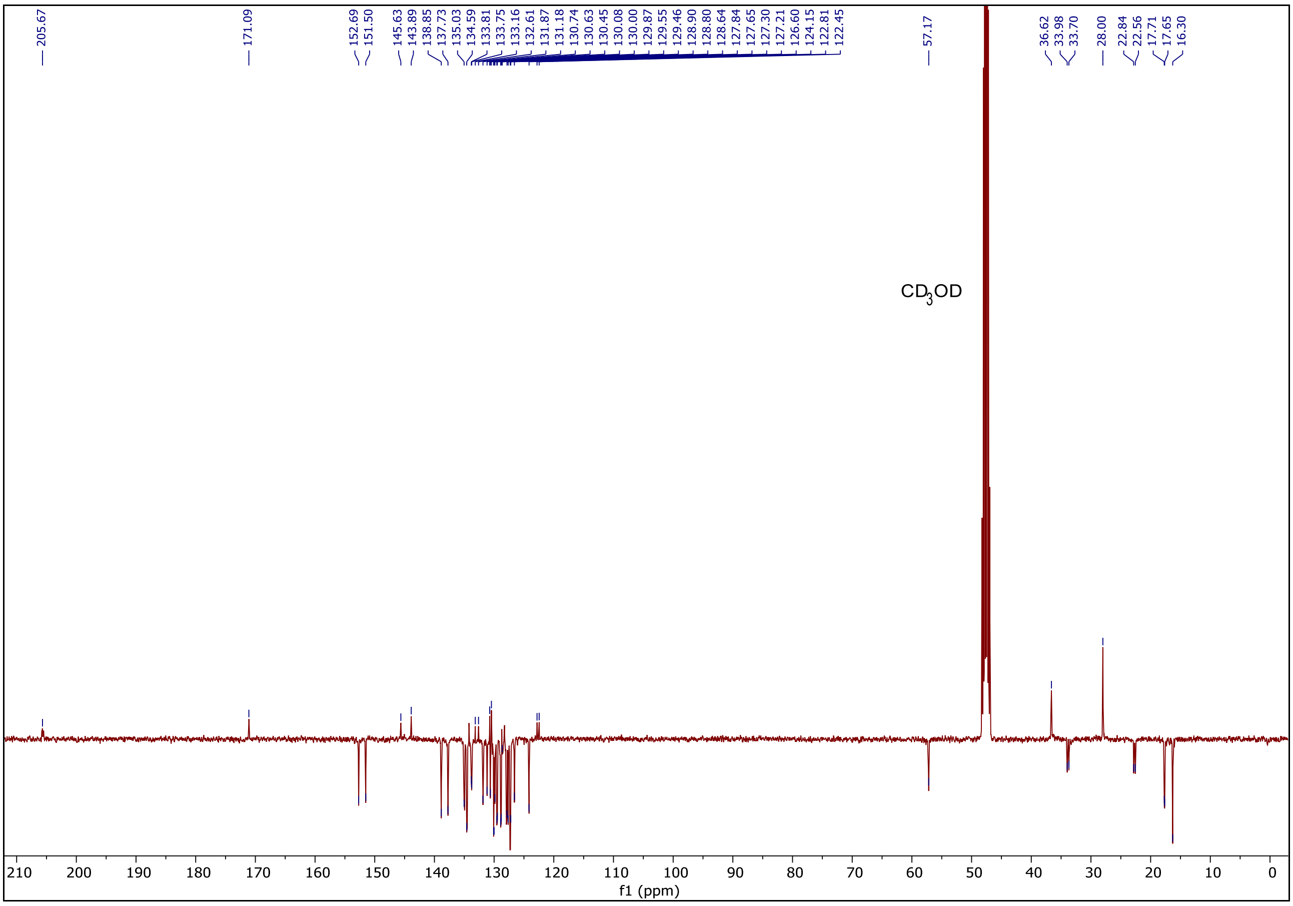

Figure S17. $^{13}C\{^{1}H\}$ DEPTQ NMR spectrum (100.6 MHz) of [Ru(L-Cys)(CO)((*R*,*R*)-Skewphos)(phen)]PF_6 (**2**$^{\boldsymbol{R}}$**-Cys**) in CD_3OD at 298 K.

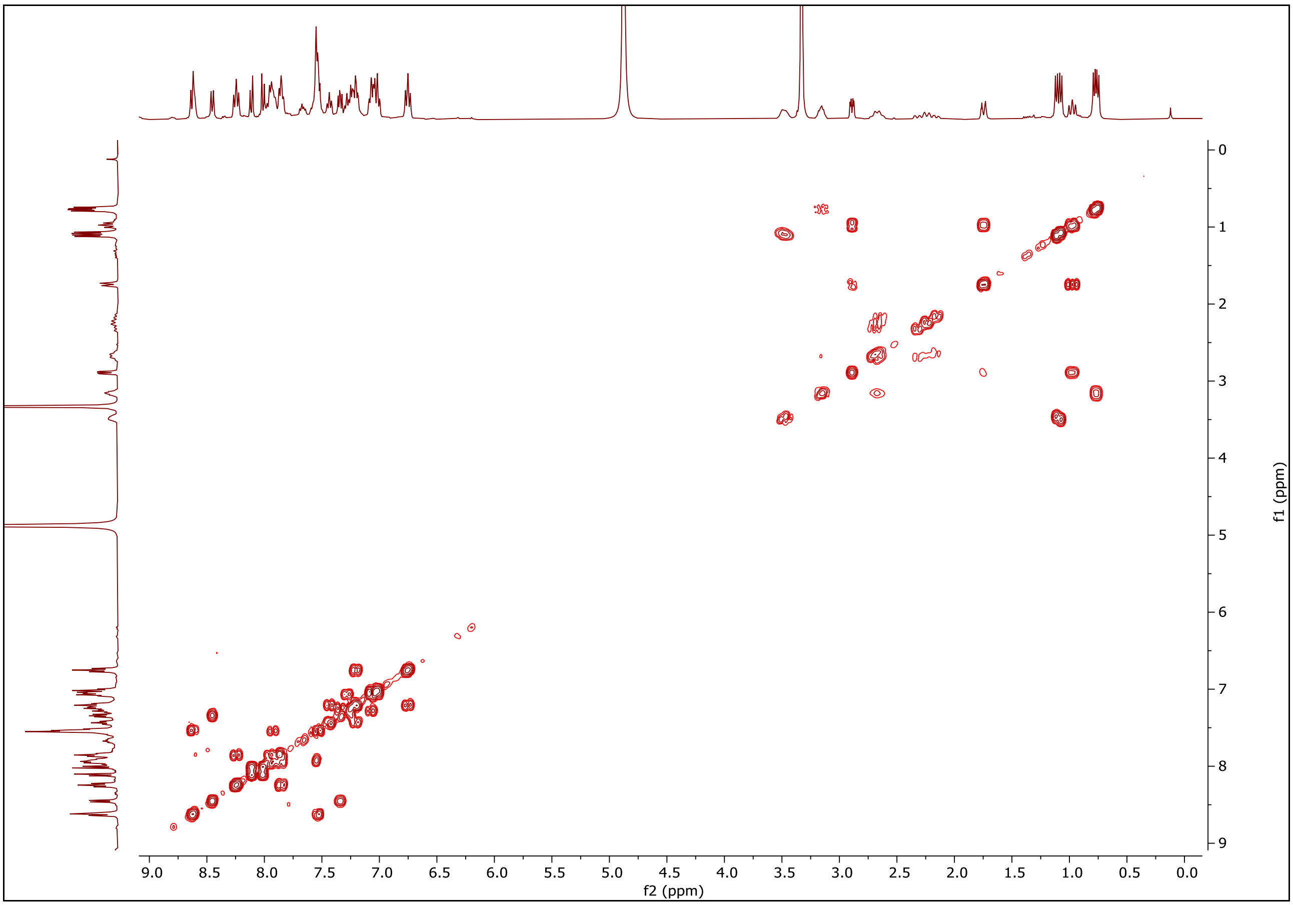

Figure S18. ^{1}H-^{1}H COSY NMR spectrum (400.1 MHz) of [Ru(L-Cys)(CO)((*R*,*R*)-Skewphos)(phen)]PF_6 (**2**$^{\boldsymbol{R}}$**-Cys**) in CD_3OD at 298 K.

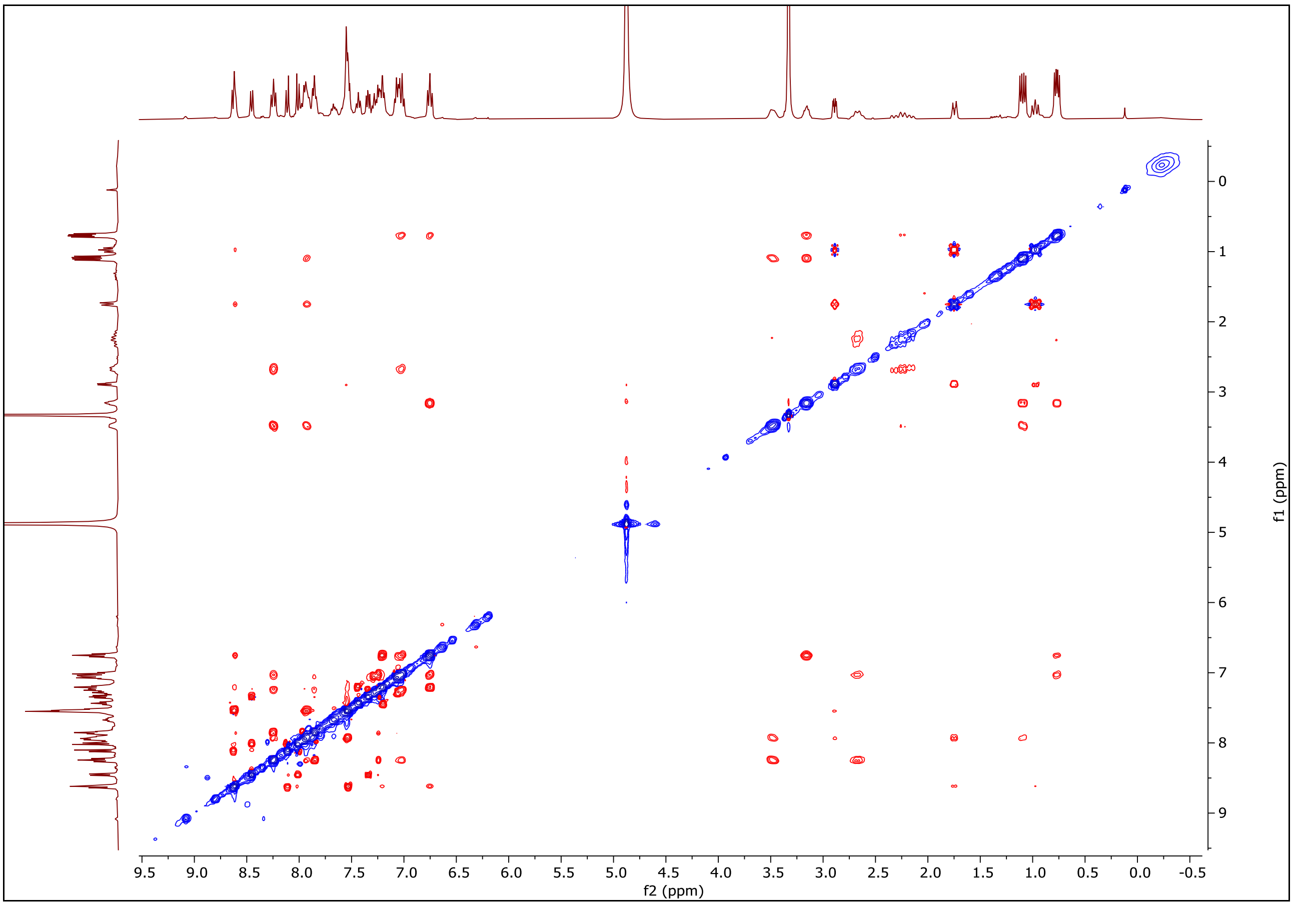

Figure S19. ^{1}H-^{1}H NOESY NMR spectrum (400.1 MHz) of [Ru(L-Cys)(CO)((*R*,*R*)-Skewphos)(phen)]PF_6 (**2^{R}-Cys**) in CD_3OD at 298 K.

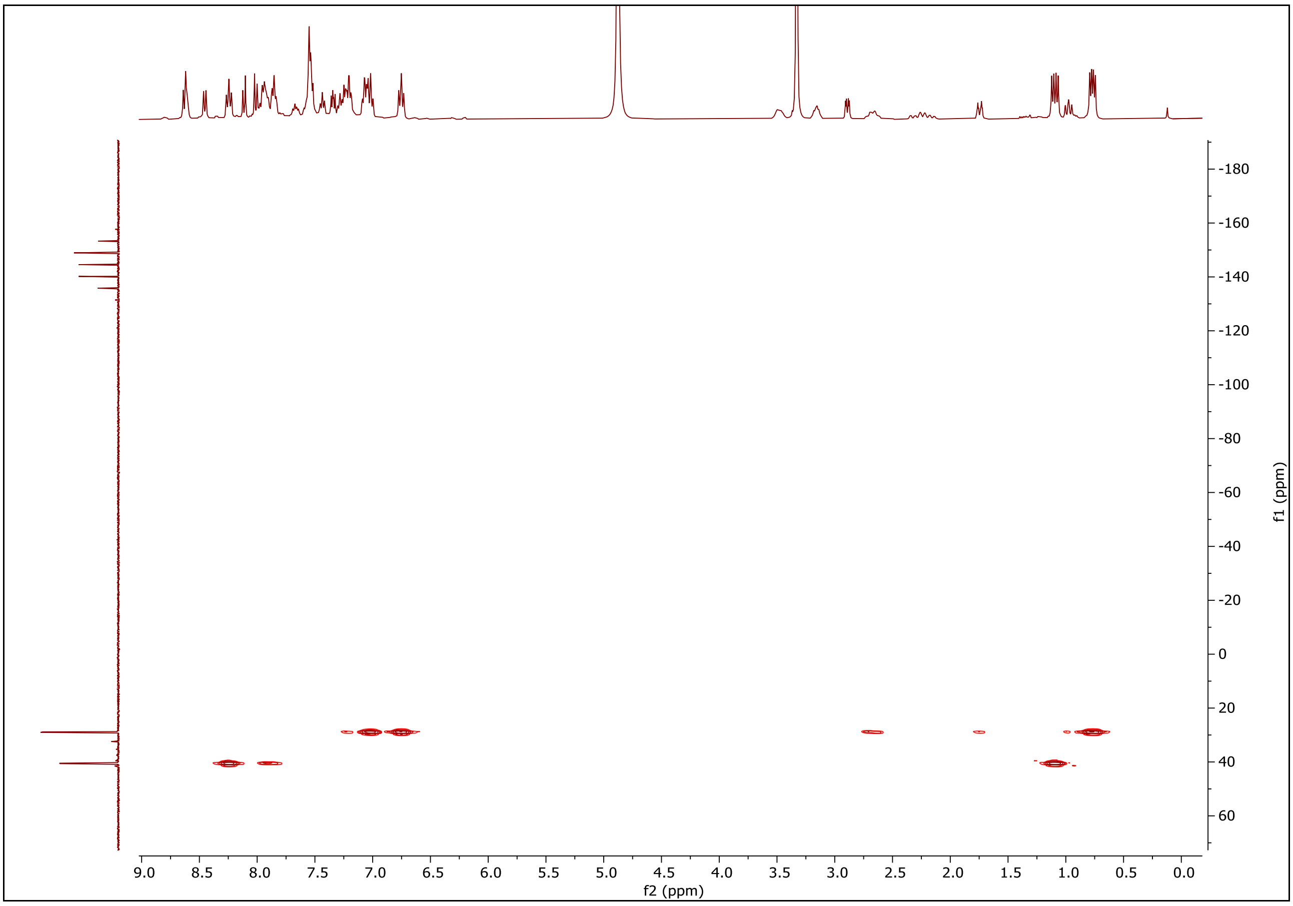

Figure S20. ^{31}P-^{1}H HMBC NMR spectrum of [Ru(L-Cys)(CO)((*R*,*R*)-Skewphos)(phen)]PF_6 (**2^{R}-Cys**) in CD_3OD at 298 K.

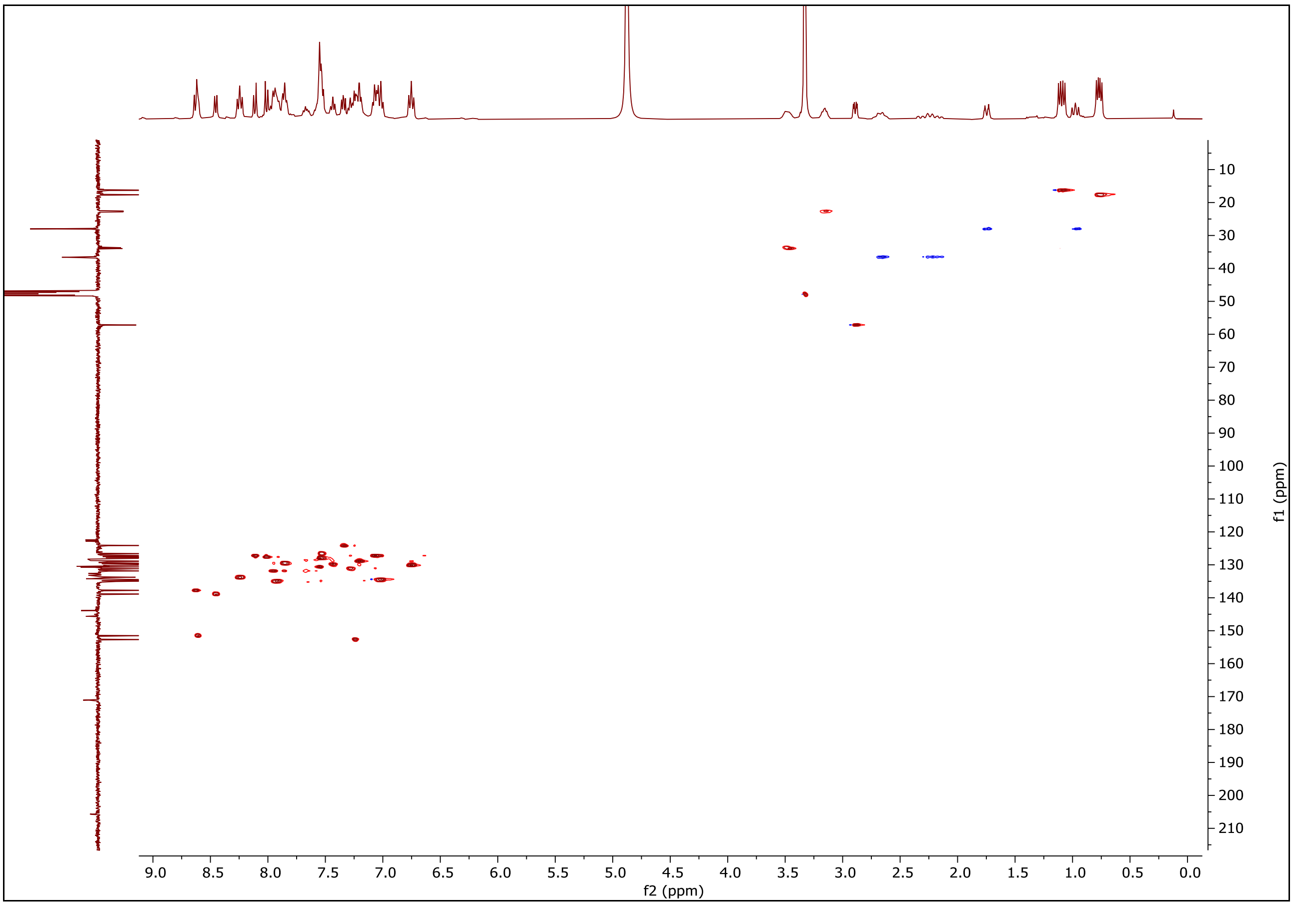

Figure S21. ^{13}C-^{1}H HSQC NMR spectrum of [Ru(L-Cys)(CO)((*R*,*R*)-Skewphos)(phen)]PF_6 (**2^{R}-Cys**) in CD_3OD at 298 K.

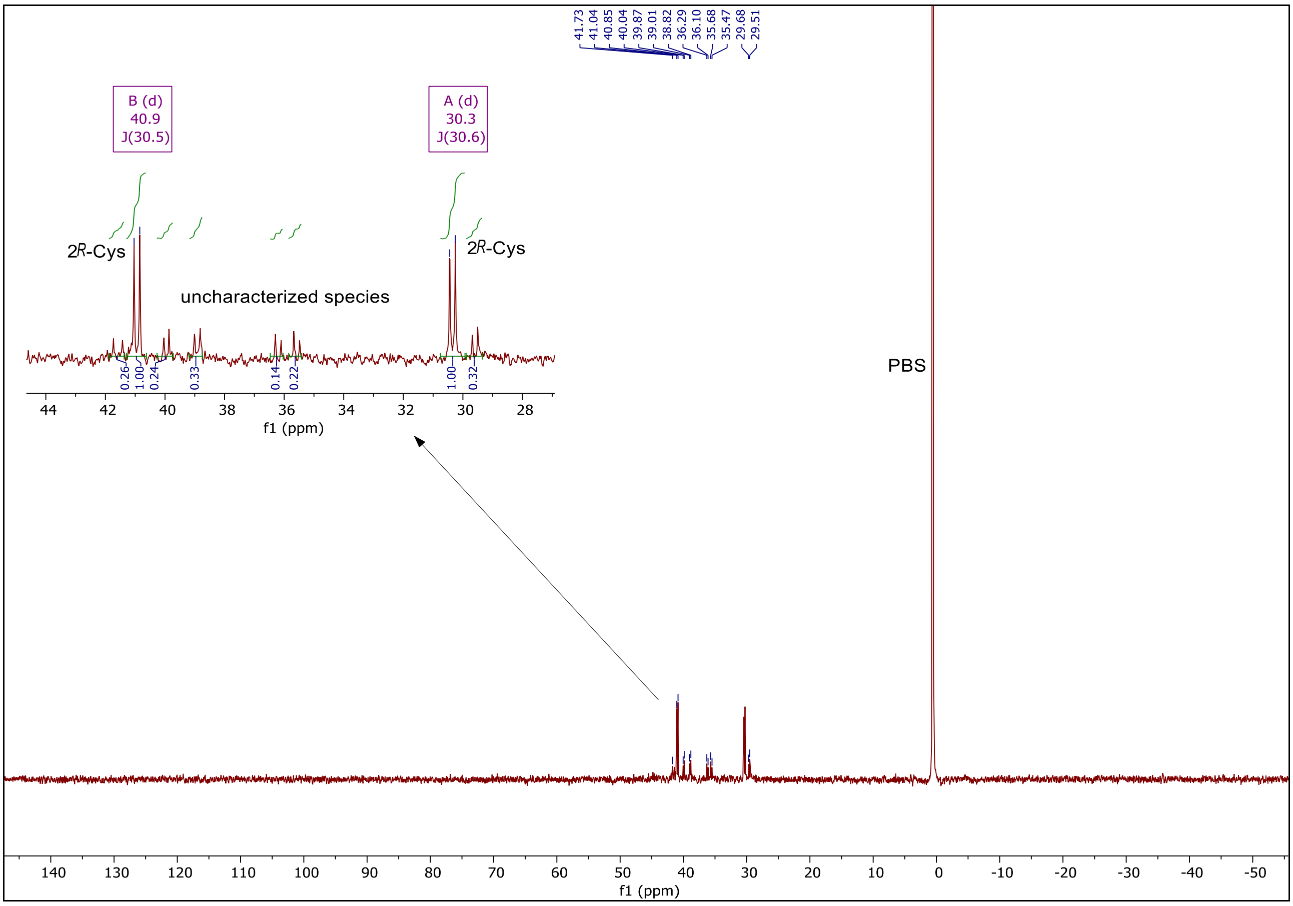

Figure S22. $^{31}P\{^{1}H\}$ NMR spectrum (162 MHz) of [Ru(η^{1}-OAc)(CO)((R,R)-Skewphos)(phen)] OAc (**2*R***) + L-Cysteine in PBS pH 6.5 (90% H_2O/10% D_2O) at 298 K.

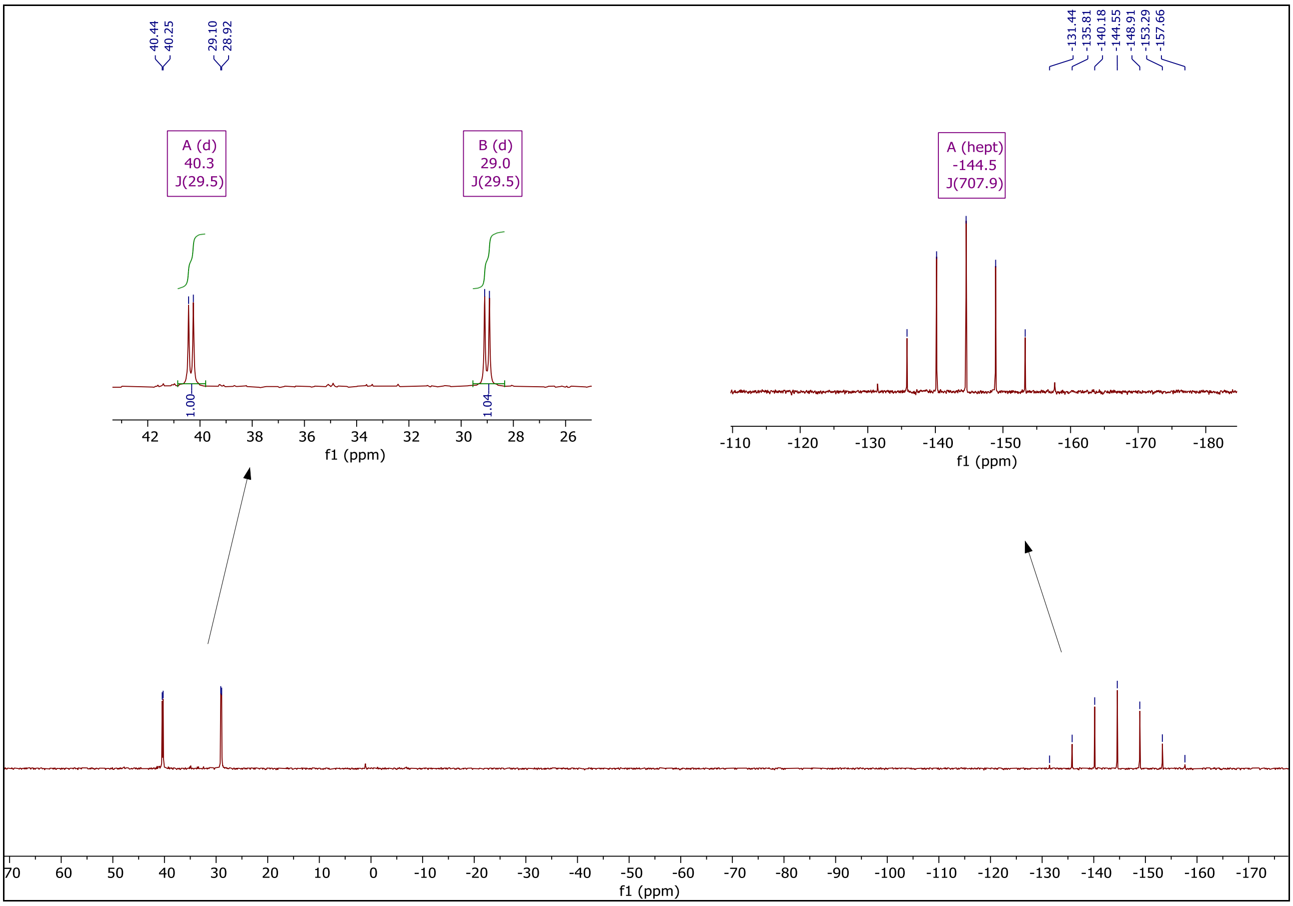

Figure S23. $^{31}P\{^{1}H\}$ NMR spectrum (162 MHz) of [Ru(L-Cys)(CO)((*S*,*S*)-Skewphos)(phen)]PF_6 (**2*^S^*-Cys**) in CD_3OD at 298 K.

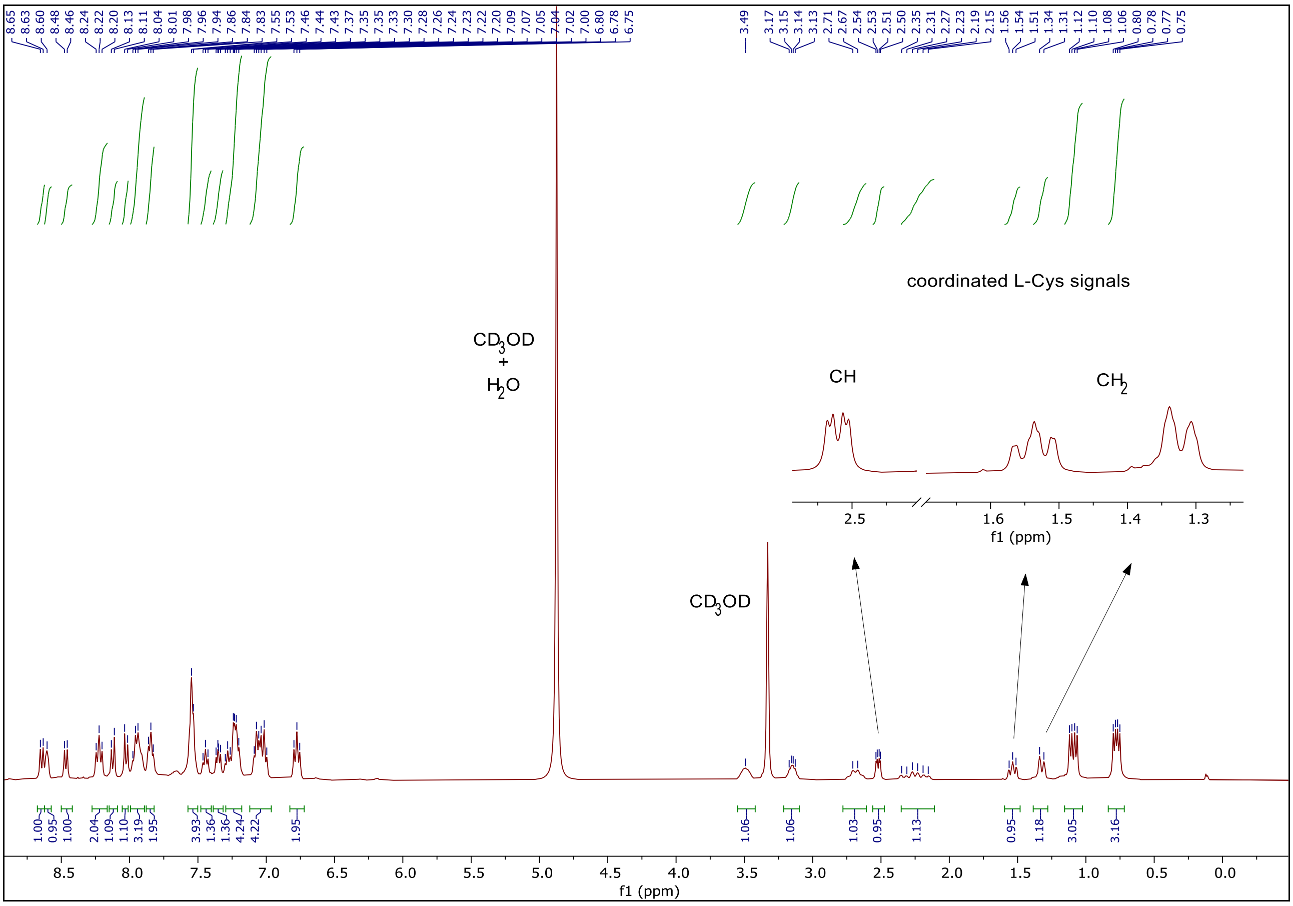

Figure S24. ^{1}H NMR spectrum (400.1 MHz) of [Ru(L-Cys)(CO)((*S*,*S*)-Skewphos)(phen)]PF_6 (**2^{S}-Cys**) in CD_3OD at 298 K.

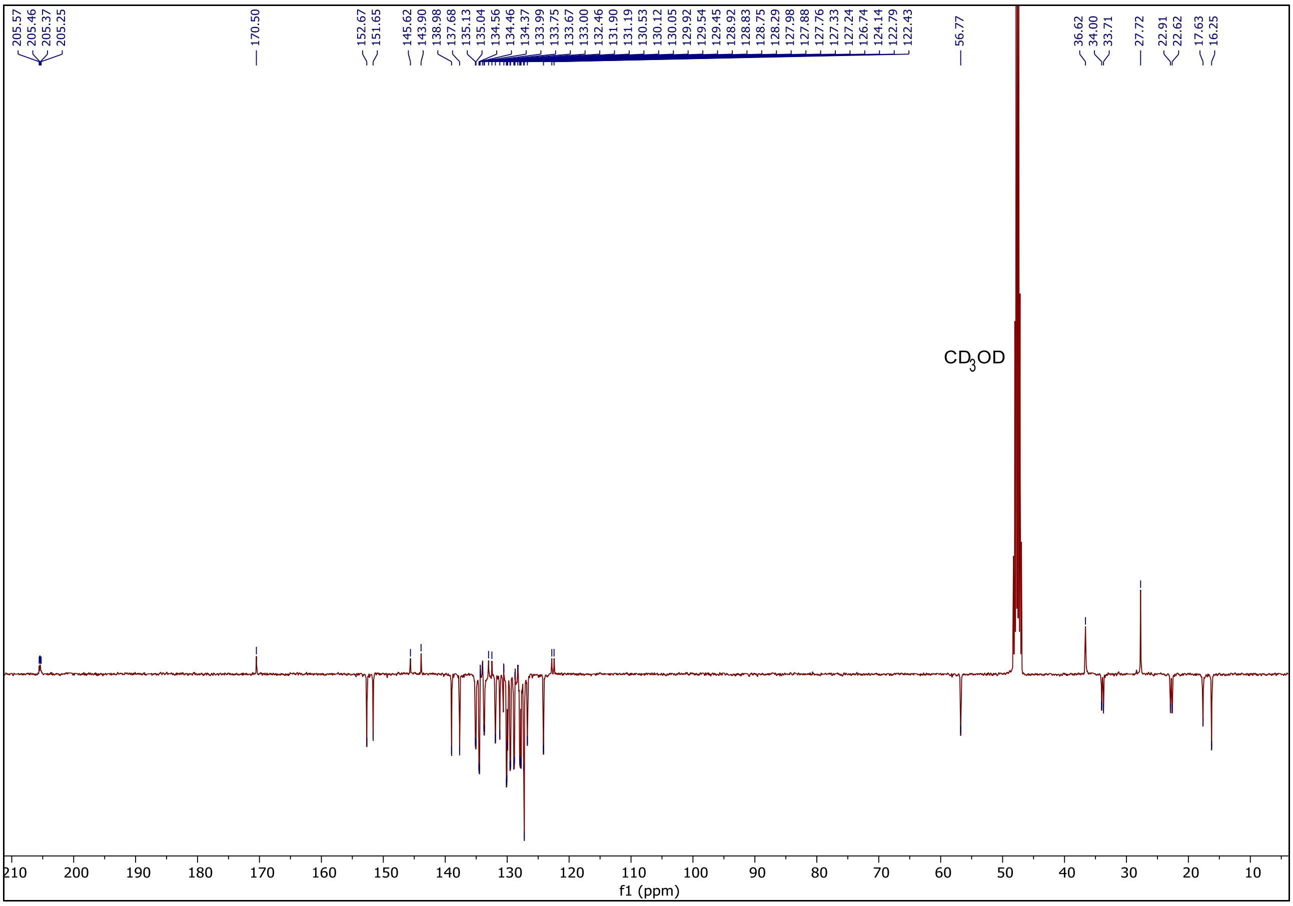

Figure S25. $^{13}C\{^1H\}$ DEPTQ NMR spectrum (100.6 MHz) of [Ru(L-Cys)(CO)((*S*,*S*)-Skewphos)(phen)]PF_6 (**2^S-Cys**) in CD_3OD at 298 K.

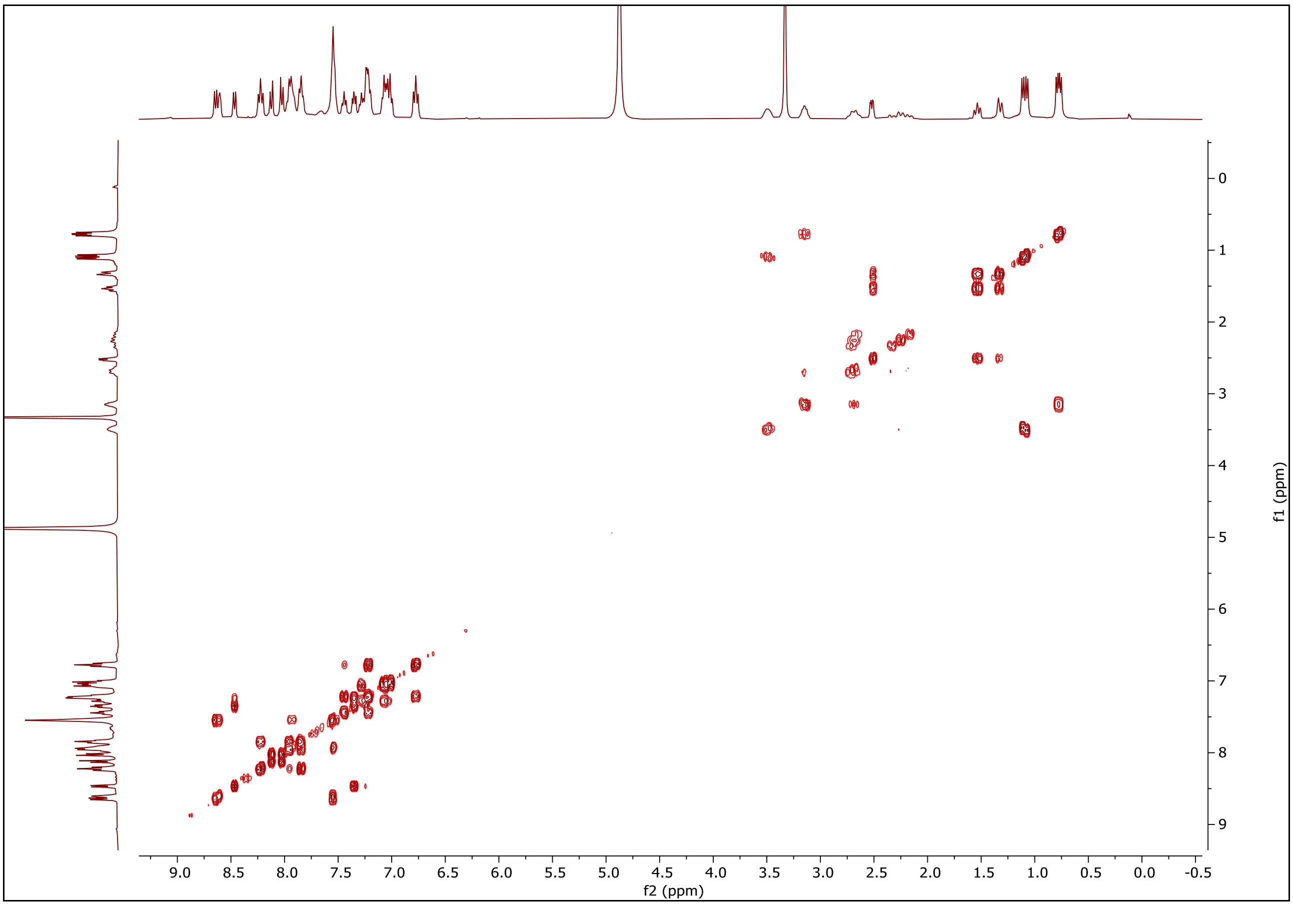

Figure S26. ^{1}H-^{1}H COSY NMR spectrum (400.1 MHz) of [Ru(L-Cys)(CO)((*S*,*S*)-Skewphos)(phen)]PF_6 (**2*S*-Cys**) in CD_3OD at 298 K.

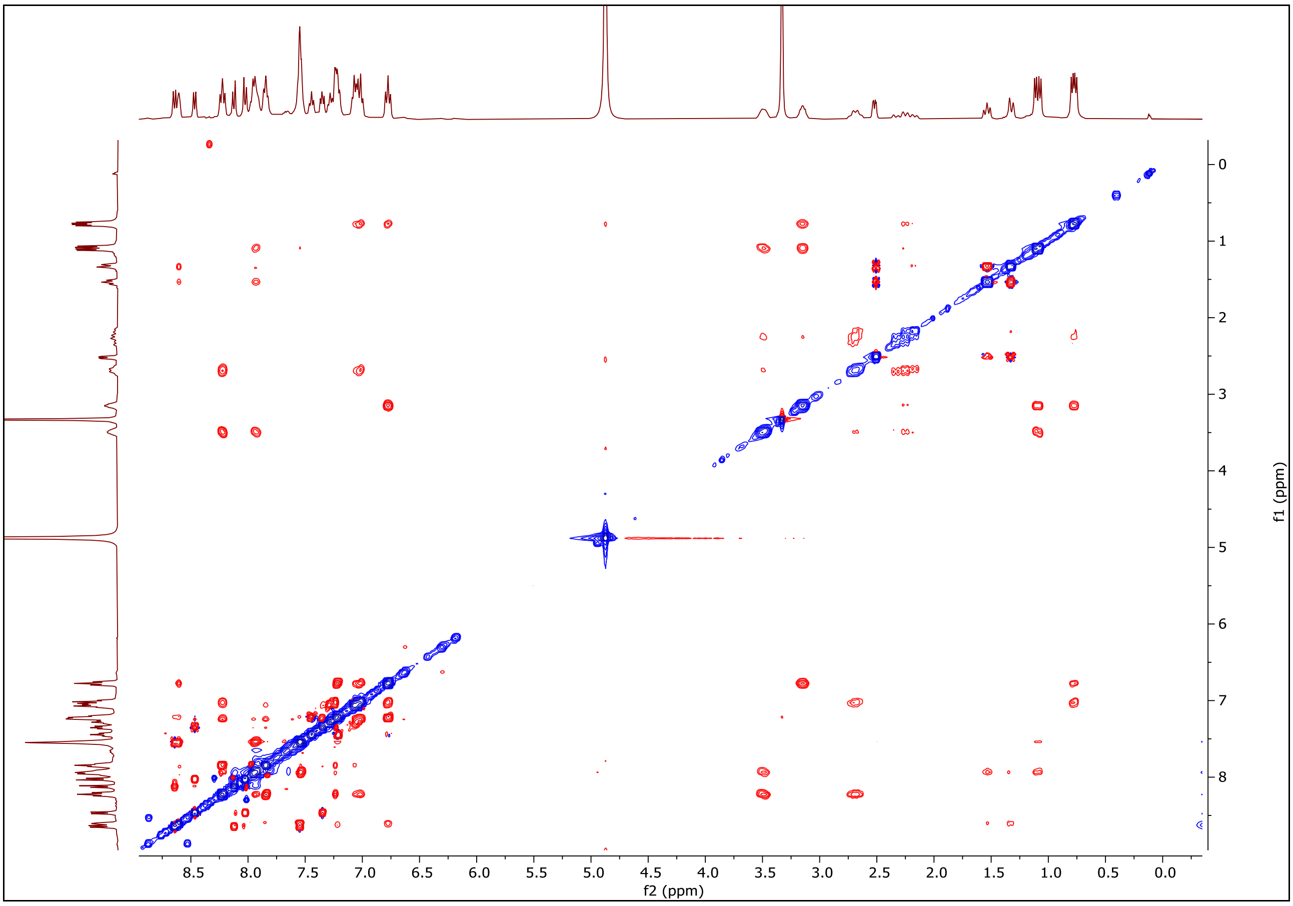

Figure S27. ^1H-^1H NOESY NMR spectrum (400.1 MHz) of [Ru(L-Cys)(CO)((*S*,*S*)-Skewphos)(phen)]PF_6 (**2^S-Cys**) in CD_3OD at 298 K.

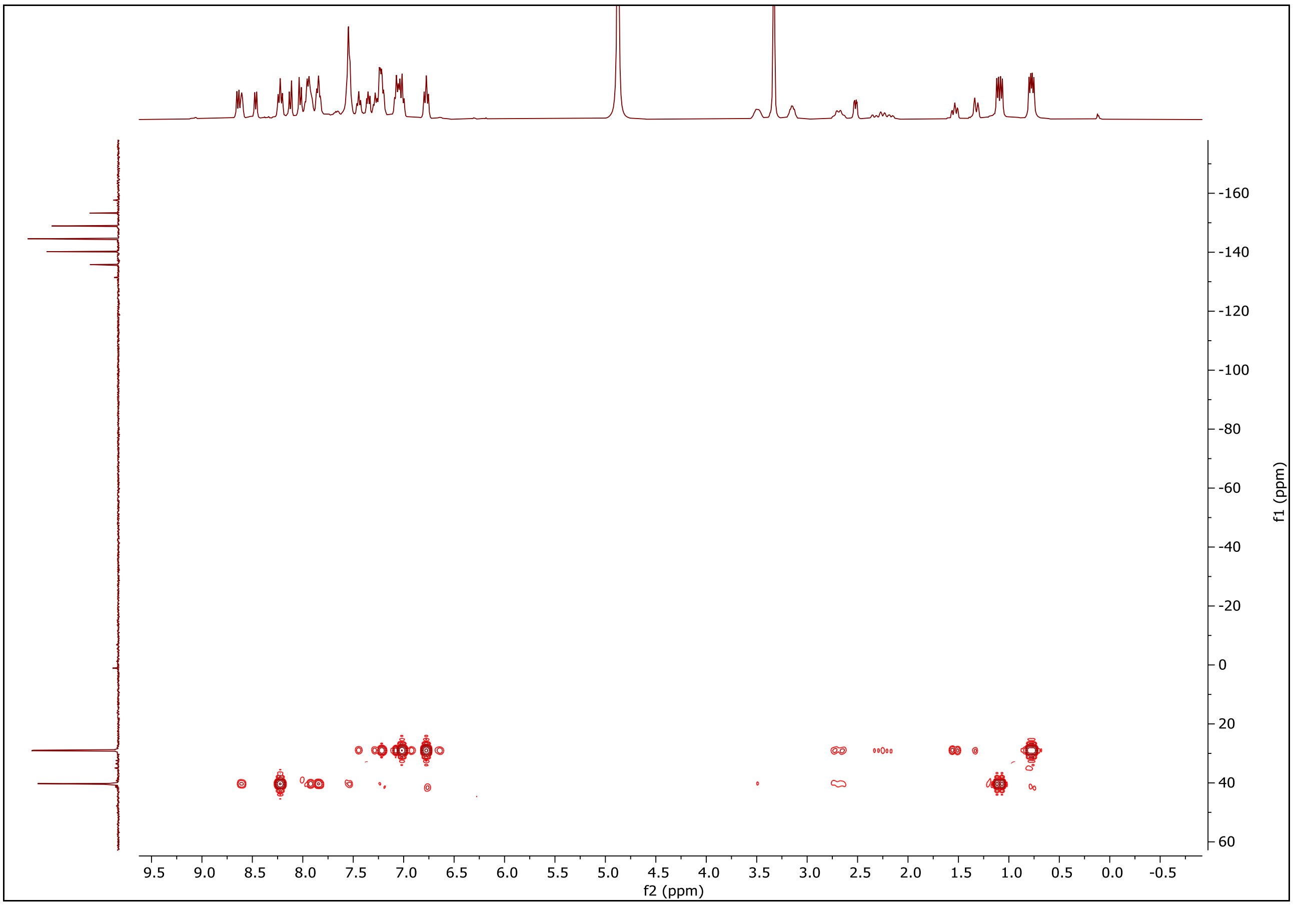

Figure S28. ^{31}P-^{1}H HMBC NMR spectrum of [Ru(L-Cys)(CO)((*S*,*S*)-Skewphos)(phen)]PF_6 (**2**^{S}**-Cys**) in CD_3OD at 298 K.

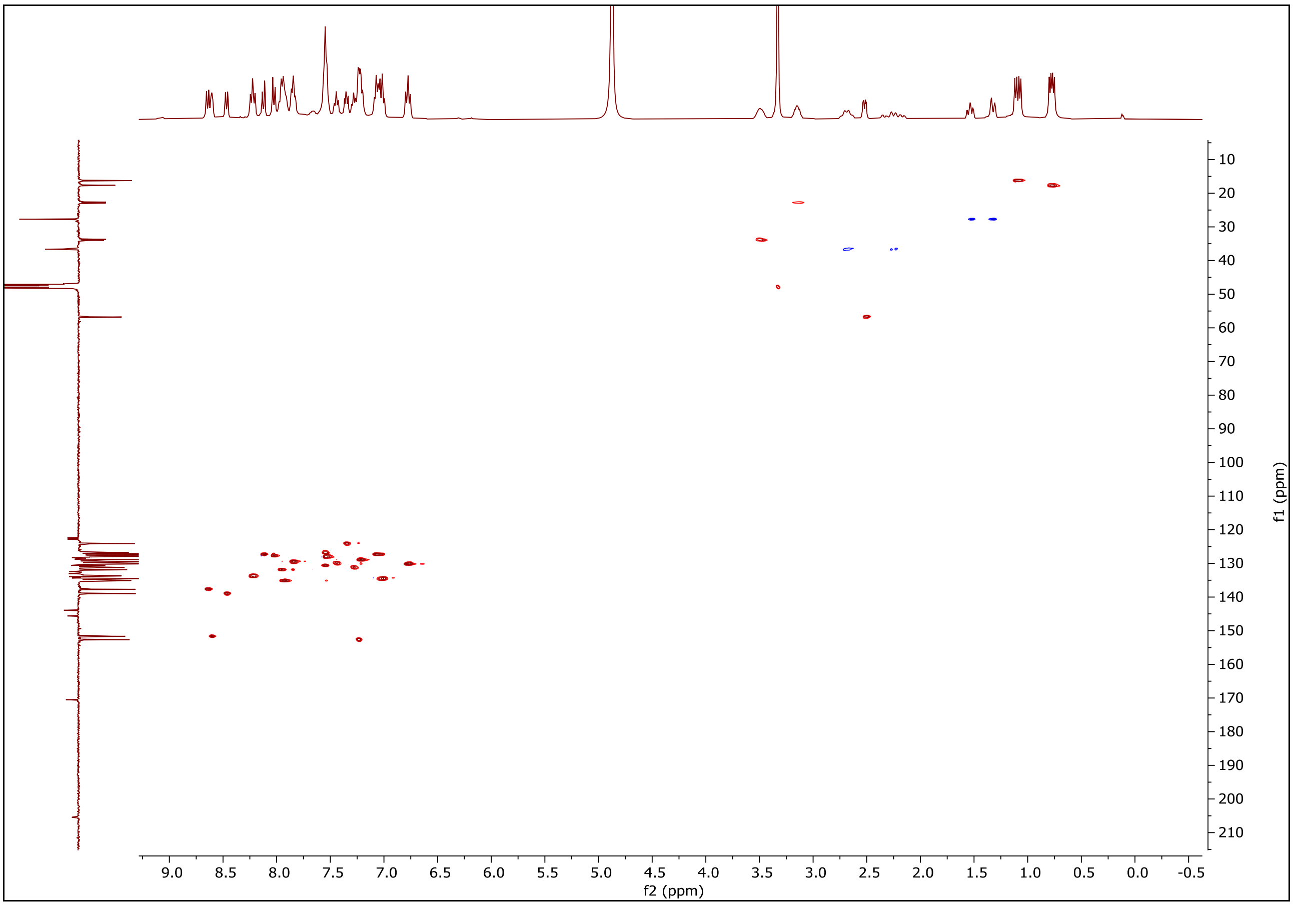

Figure S29. ^{13}C-^{1}H HSQC NMR spectrum of [Ru(L-Cys)(CO)((*S*,*S*)-Skewphos)(phen)]PF_6 (**2^{S}-Cys**) in CD_3OD at 298 K.

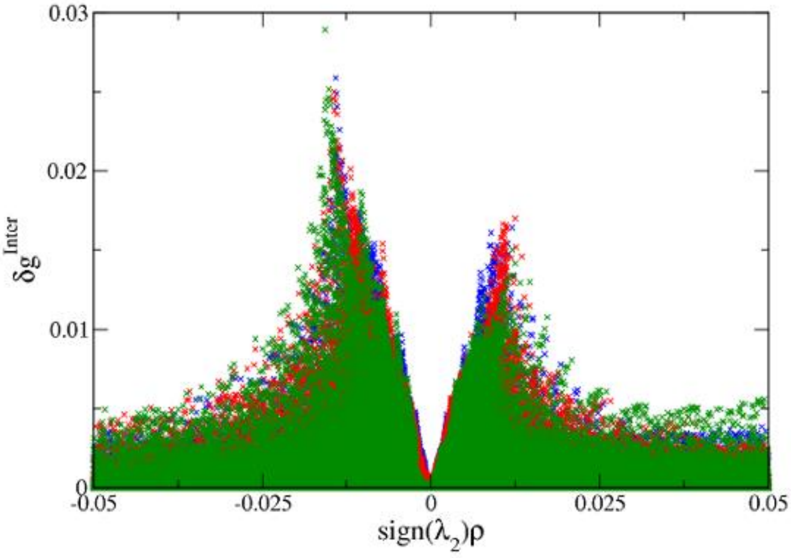

Figure S30. δg^{inter} plot for: a) *trans*-**2^{R+}** green cross; b) **2$^{R'+}$** red cross; and c) **2^{R+}** blue cross.

Table S1. Selected bonds lengths (Å) and angles (°) for the isomer of [Ru(η^1-OAc)(CO)((R,R)-Skewphos)(phen)]$^+$ (atom labels in Figure S2).

Bonds			
bond	2^{R+}	*trans*-2^{R+}	$2^{R'+}$
Ru-P1	2.374	2.444	2.392
Ru-P2	2.379	2.395	2.377
Ru-N1	2.219	2.180	2.220
Ru-N2	2.144	2.187	2.146
Ru-CO	1.842	1.847	1.843
Ru-OAc	2.108	2.103	2.110
Angles			
	2^{R+}	*trans*-2^{R+}	$2^{R'+}$
P1-Ru-P2	92.3	92.66	96.51
N1-Ru-N2	76.56	76.78	76.82

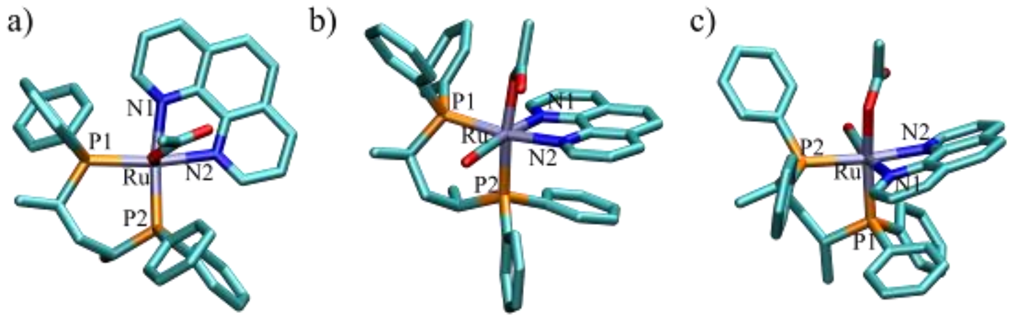

Figure S31. Minimum energy structures for: a) *trans*-$\mathbf{2}^{R+}$; b) $\mathbf{2}^{R'+}$; and c) $\mathbf{2}^{R+}$ with atom label (see Table S1)

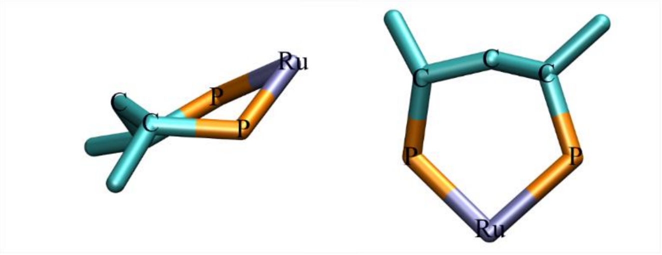

Figure S32. Boat conformation of six-membered-cyclo structures for $\mathbf{2}^{R+}$ (the other atoms were removed for clarity).

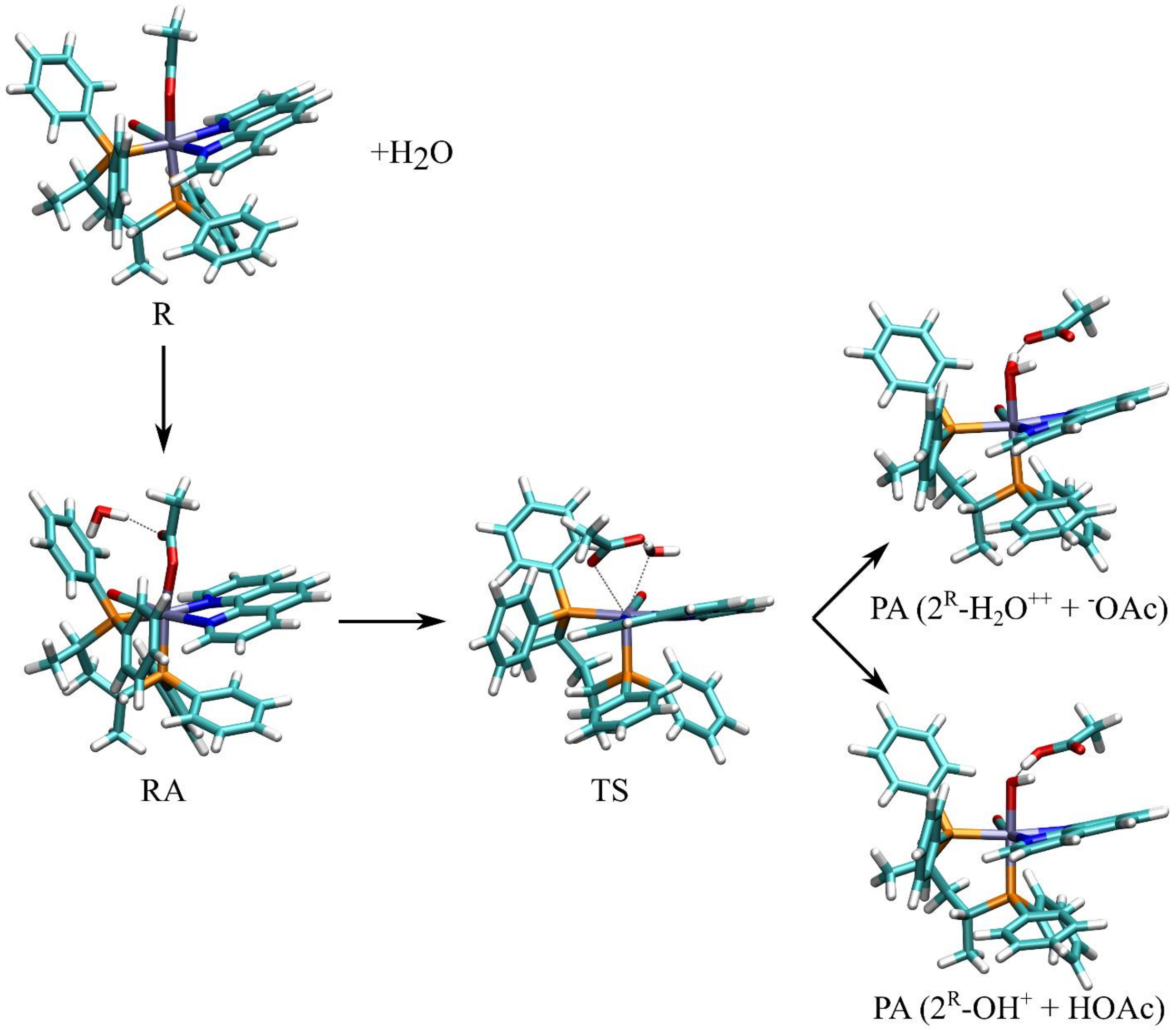

Figure S33: minimum energy structures for R, RA, TS, PA (**2*R*-H₂O**$^{++}$ and **2*R*-OH**$^{+}$) of the hydrolysis reaction of **2*R*+**.

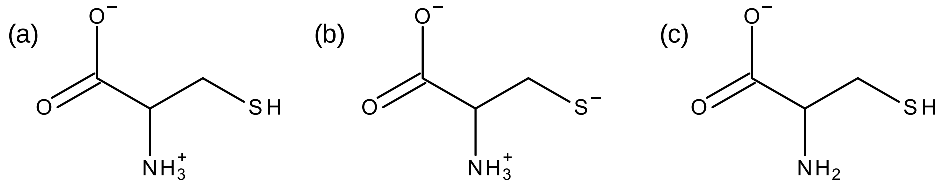

Scheme S1 Species of L-cysteine present in acidic (pH 5-7) and basic (pH ~ 9) solution: (a) HS-(*S*)-cysteine (b) S-(*S*)-cysteine, (c) HS-(*S*)-cysteine$^{(-)}$.

Table S2. Reactive adduct formation ($\Delta G_{fRA} = G_{RA} - G_{reactants}$), activation ($\Delta G^{\ddagger} = G_{TS} - G_{RA}$) and reaction ($\Delta G_r' = G_{PA} - G_{RA}$ and $\Delta G_r = G_{PA} - G_R$) free energies (kcal mol^{-1}). To make the table more readable, the protonation state of the S atom of the (*S*)-cysteine is reported.

**Reactions**	**ΔG_{fRA}**	**$\Delta G^{\ddagger}$**	**$\Delta G_r'$**	**ΔG_r**
2^{R+} + H_2O → 2^R-H_2O^{++} + $^-$OAc	–2.0	18.4	4.4	2.4
2^{R+} + H_2O → 2^R-OH^+ + HOAc	–2.0	18.4	4.3	2.3
Basic conditions				
2^R-H_2O^{++} + $^-$S-(*S*)-Cysteine → Ru-S-(*S*)-Cysteine-NH_3^+ + H_2O	-17.2	11.2	-15.7	-33.0
2^R-OH^+ + $^-$S-(*S*)-Cysteine → Ru-S-(*S*)-Cysteine-NH_2 + H_2O	-7.2	14.3	-14.8	-22.1
Acidic/neutral conditions				
2^{R+} + HS-(S)-Cysteine → Ru-S-(*S*)-Cysteine + HOAc	-5.6	32.9	-0.1	-5.7
2^R-H_2O^{++} + HS-(*S*)-Cysteine-NH_3^+ → Ru-HS-(*S*)-Cysteine-NH_3^+ + H_2O	-0.4	10.1	-3.8	-4.3
2^R-H_2O^{++} + $^-$OCC-(*S*)-Cysteine-NH_3^+ → Ru-OOC-(*S*)-Cysteine-NH_3^+ + H_2O	-13.4	9.7	-6.0	-19.4
2^R-OH^+ + HS-(*S*)-Cysteine-NH_3^+ → Ru-S-(*S*)-Cysteine-NH_3^+ + H_2O	-11.0	17.4	-9.6	-20.6
2^R-OH^+ + $^-$OOC-(*S*)-Cysteine-NH_3^+ → Ru-OOC-(*S*)-Cysteine-NH_2^+ + H_2O	-16.5	15.5	-6.4	-23.1

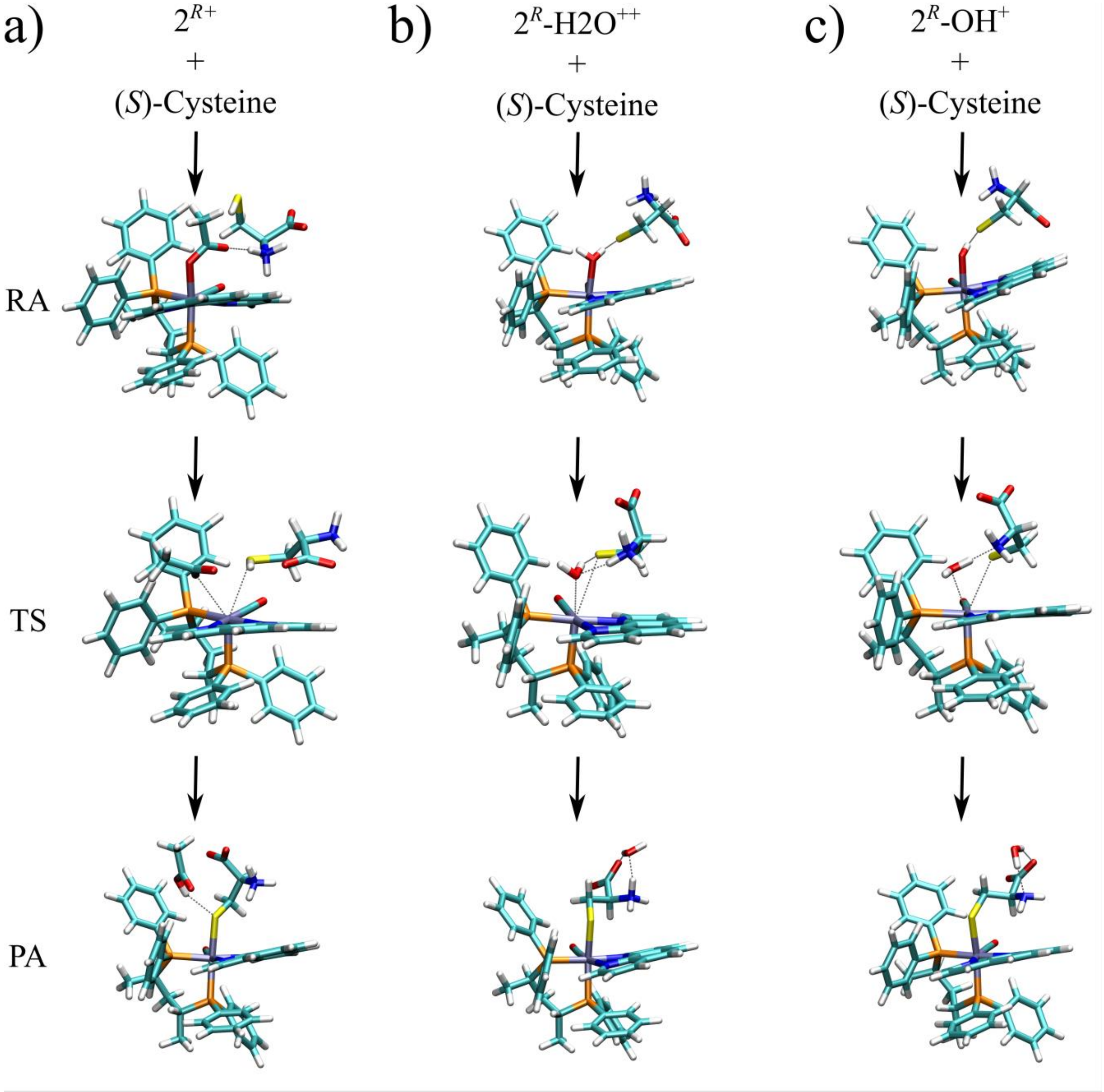

Figure S34. (a) Minimum energy structures for RA, TS and PA for the reactions between zwitterionic form of SH-(*S*)-Cysteine and $\mathbf{2^{R+}}$. Reactions between anionic form of $^{-}$S-(*S*)-Cysteine and (b) $\mathbf{2^{R}}$**-**$\mathbf{H_2O^{++}}$ (c) $\mathbf{2^{R}}$**-**$\mathbf{OH^{+}}$ in basic conditions. A proton transfer between NH_3 and H_2O was observed in 2^{R+}-OH.

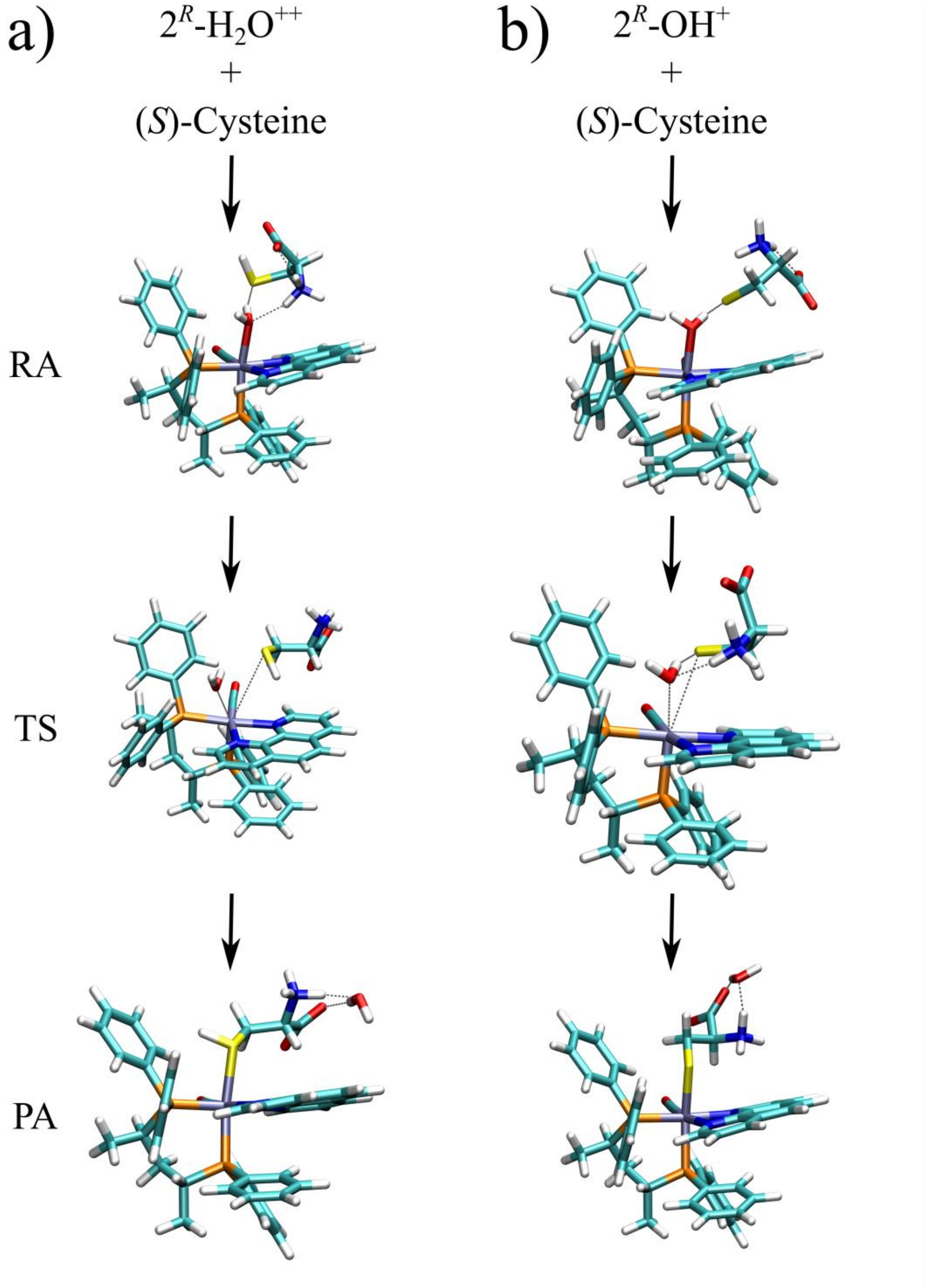

Figure S35. Minimum energy structures for RA, TS and PA for the reactions between zwitterionic form of SH-(*S*)-Cysteine in acidic-neutral conditions and: a) **2^R-H_2O^{++}** b) **2^R-OH^+**. A proton transfer from SH of (*S*)-cysteine to OH of group of **2^R-OH^+** was observed.

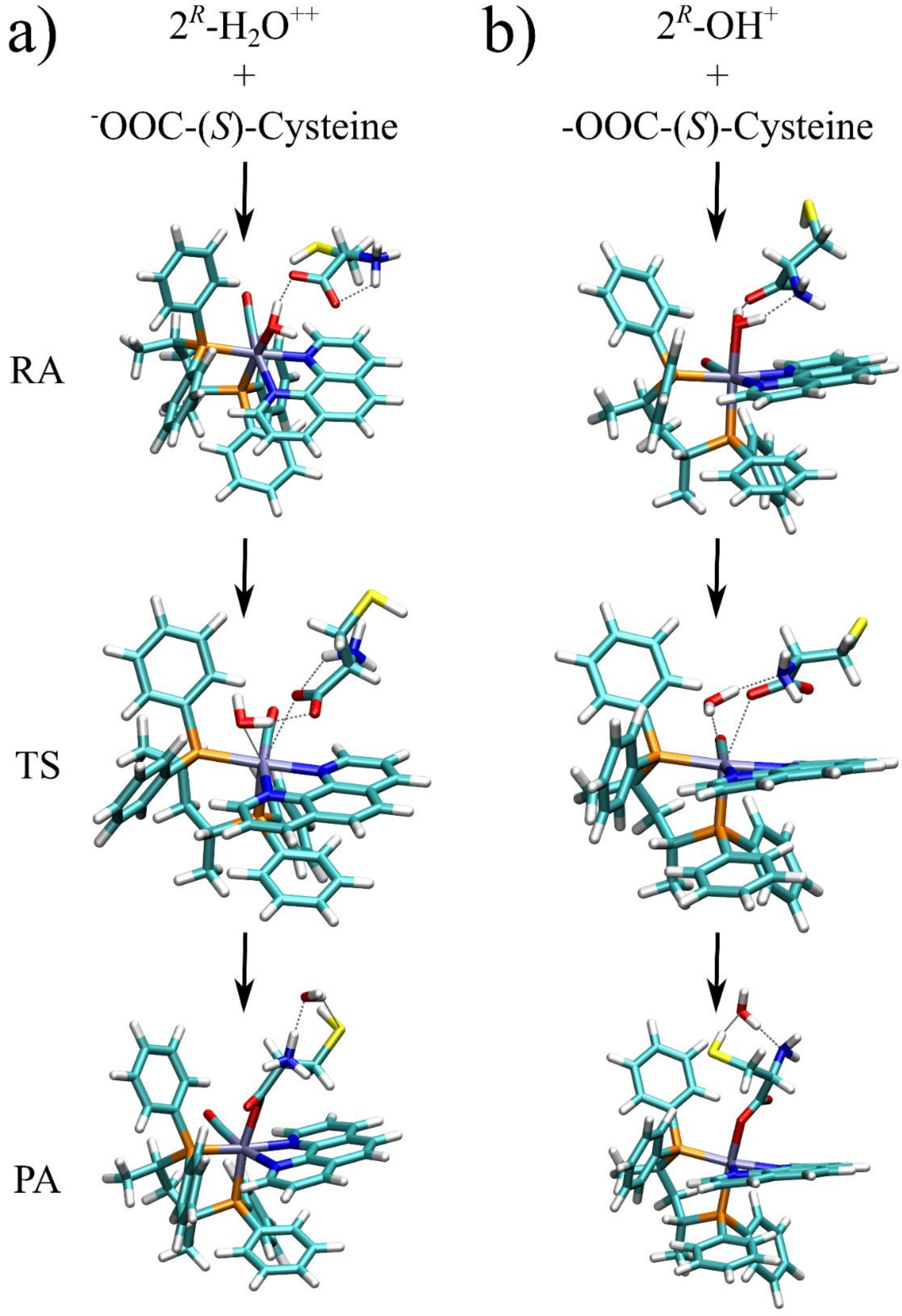

Figure S36. Minimum energy structures for RA, TS and PA for the side reactions between zwitterionic form of $^{-}$OOC-(*S*)-Cysteine in acidic-neutral conditions and: a) $\mathbf{2^{R}}$**-H**$_2$**O**$^{++}$ b) $\mathbf{2^{R}}$**-OH**$^{+}$, a proton transfer from NH_3^+ of (*S*)-cysteine to OH of group of $\mathbf{2^{R}}$**-OH**$^{+}$ was observed.

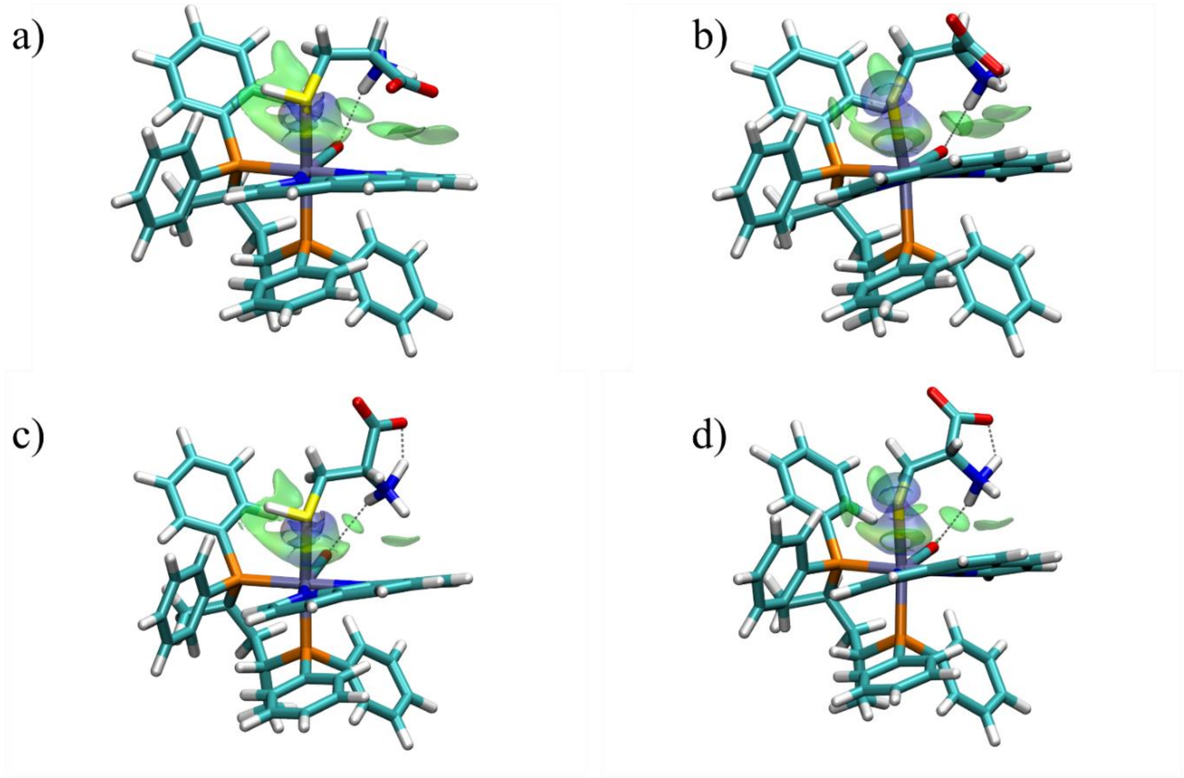

Figure S37 Minimum energy structures for: a) **2^{R++}-SH-(*S*)-Cysteine**; b) **2^{R+}-S-(*S*)-Cysteine**; c) **2^{R++}-SH-(*R*)-Cysteine**; d) **2^{R+}-S-(*R*)-Cysteine**. δg^{inter} isosurfaces (isovalue 0.0055 a.u.) in green.

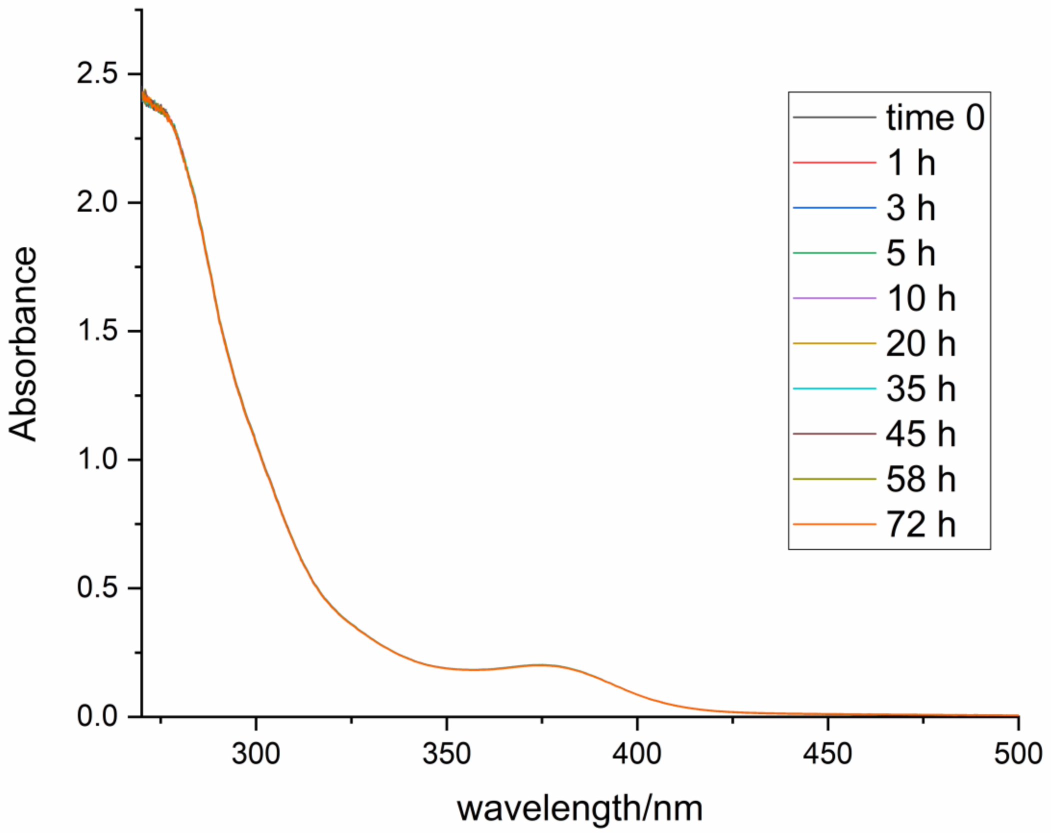

Figure S38. Selection of electronic spectra of the complex $\mathbf{2}^{S}$ in DMSO acquired during a 72-h scanning kinetics.

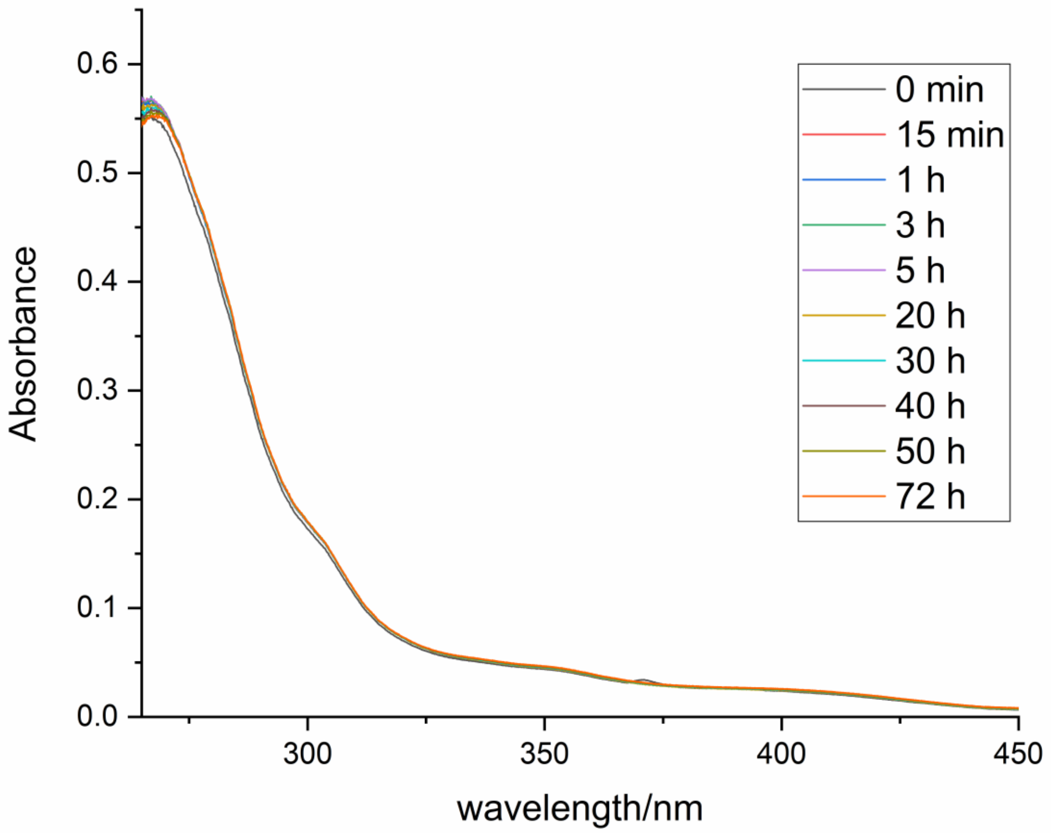

Figure S39. Selection of electronic spectra of the complex $\mathbf{4}^{S}$ in DMSO acquired during a 72-h scanning kinetics.

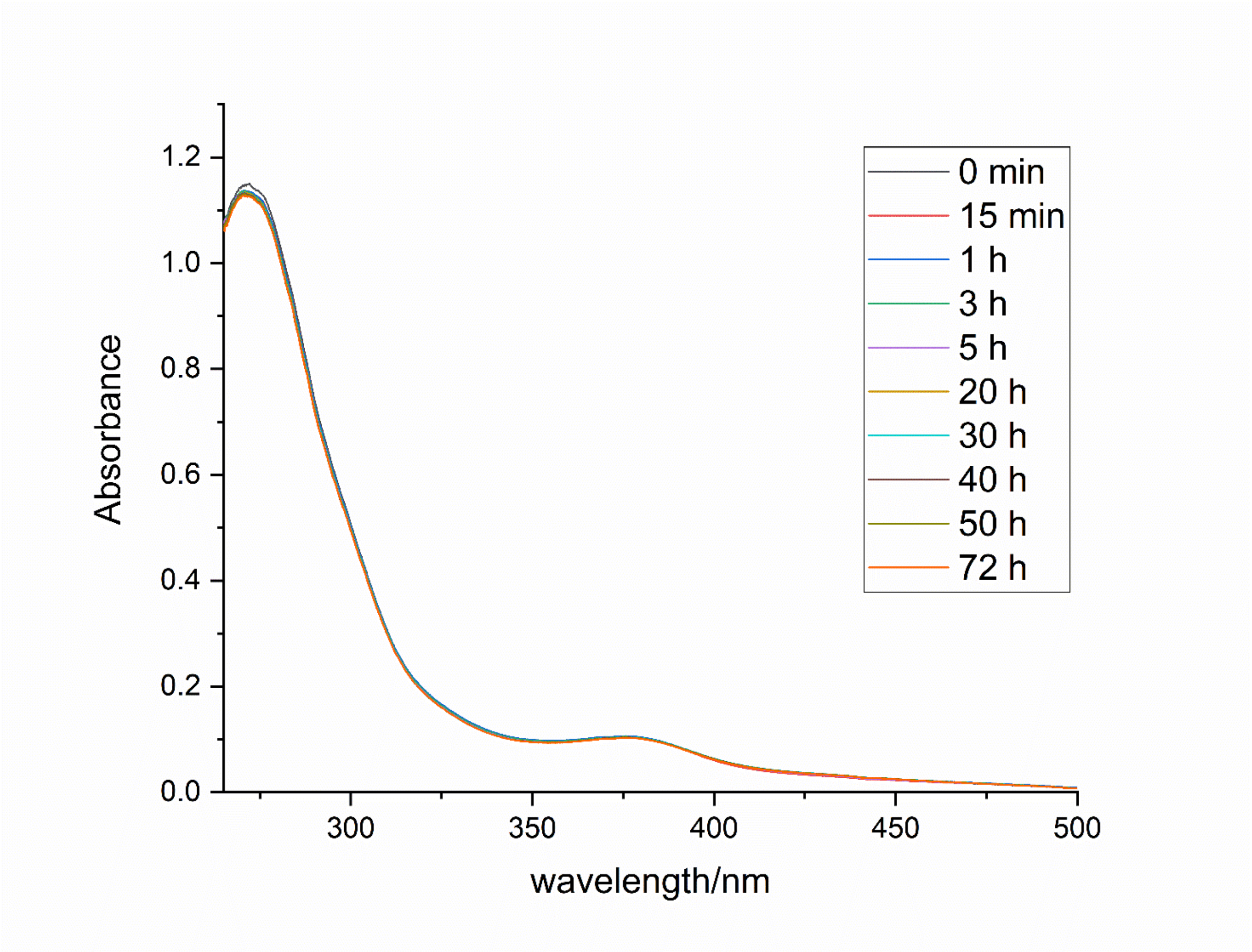

Figure S40. Selection of electronic spectra of the complex **3**^{S} in DMSO acquired during a 72-h scanning kinetics.

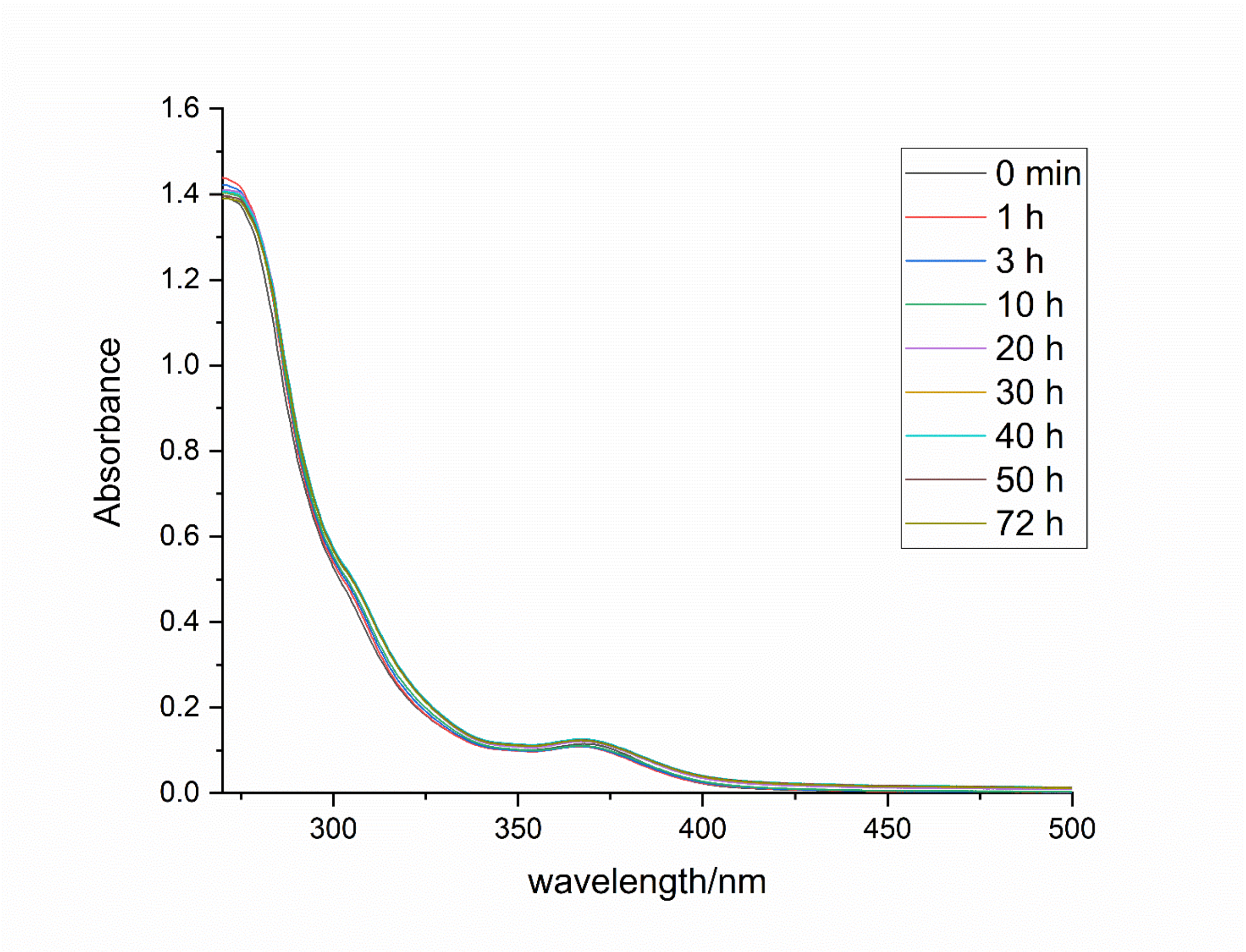

Figure S41. Selection of electronic spectra of the complex $\mathbf{2}^{S}$ in water acquired during a 72-h scanning kinetics.

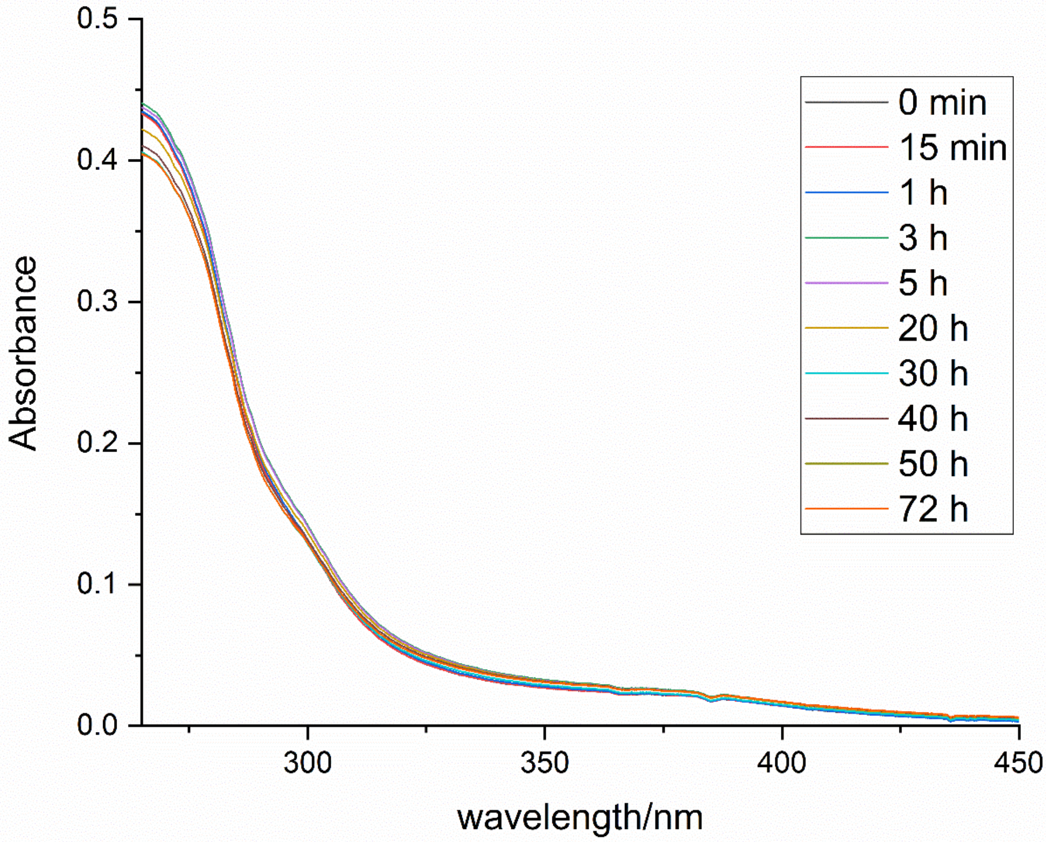

Figure S42. Selection of electronic spectra of the complex **4**^{S} in water acquired during a 72-h scanning kinetics.

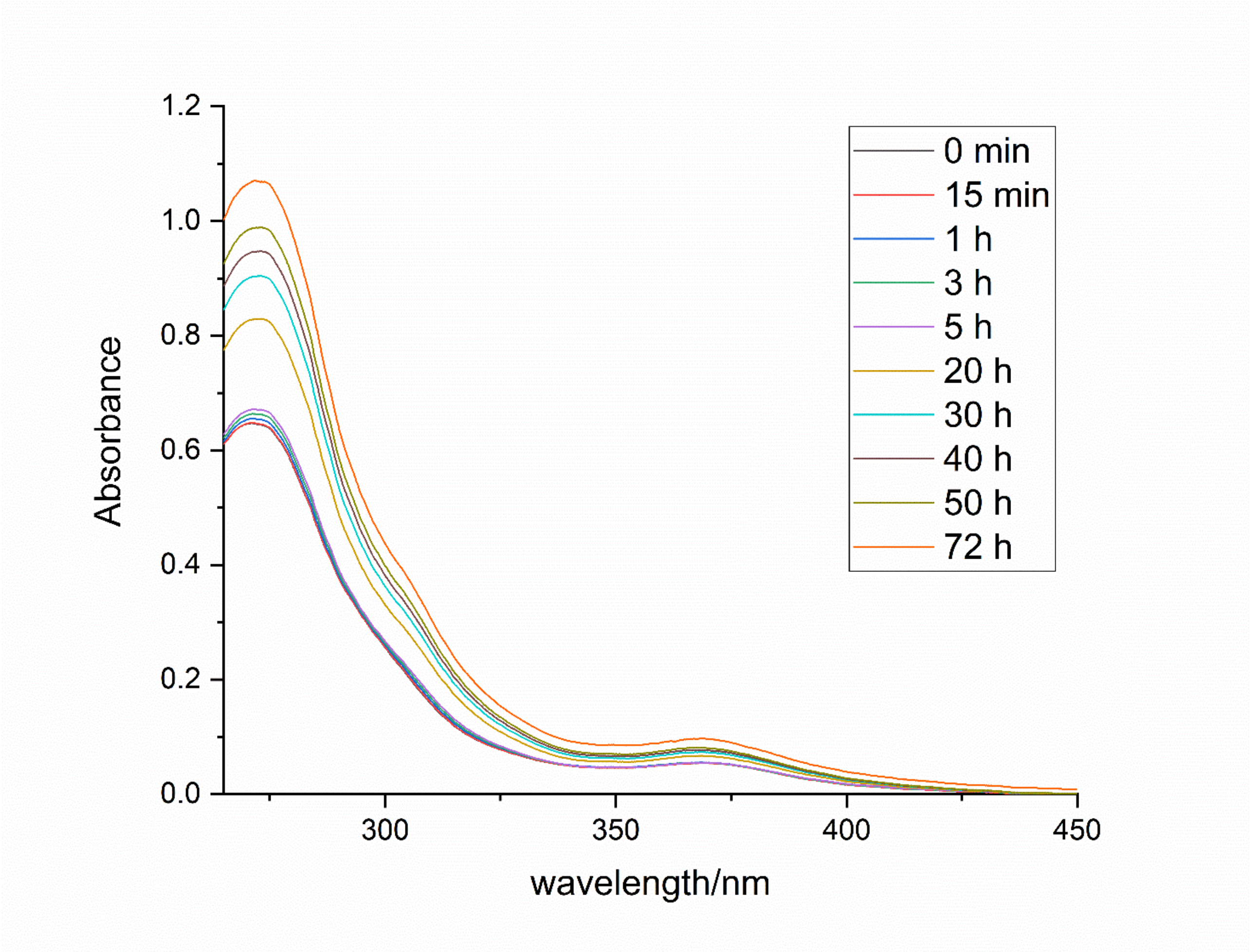

Figure S43. Selection of electronic spectra of the complex **3**^{S} in water acquired during a 72-h scanning kinetics.

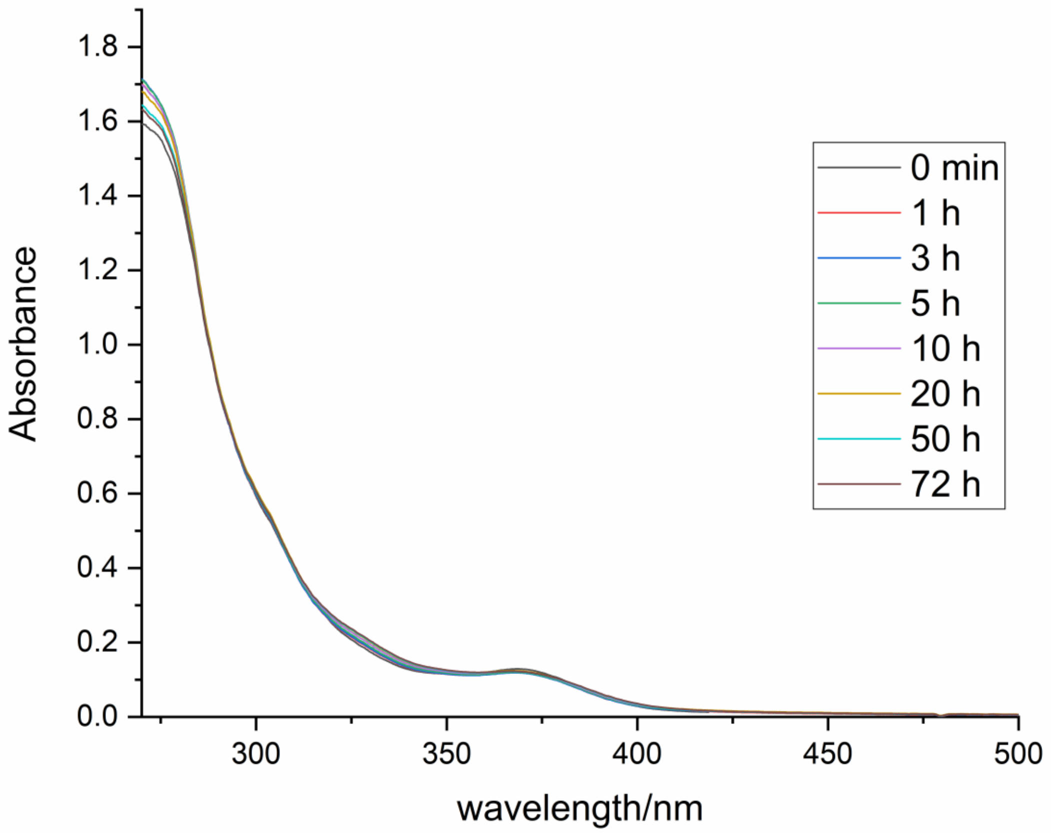

Figure S44. Selection of electronic spectra of the complex **2**^{S} in saline solution (0.9% w/v) acquired during a 72-h scanning kinetics.

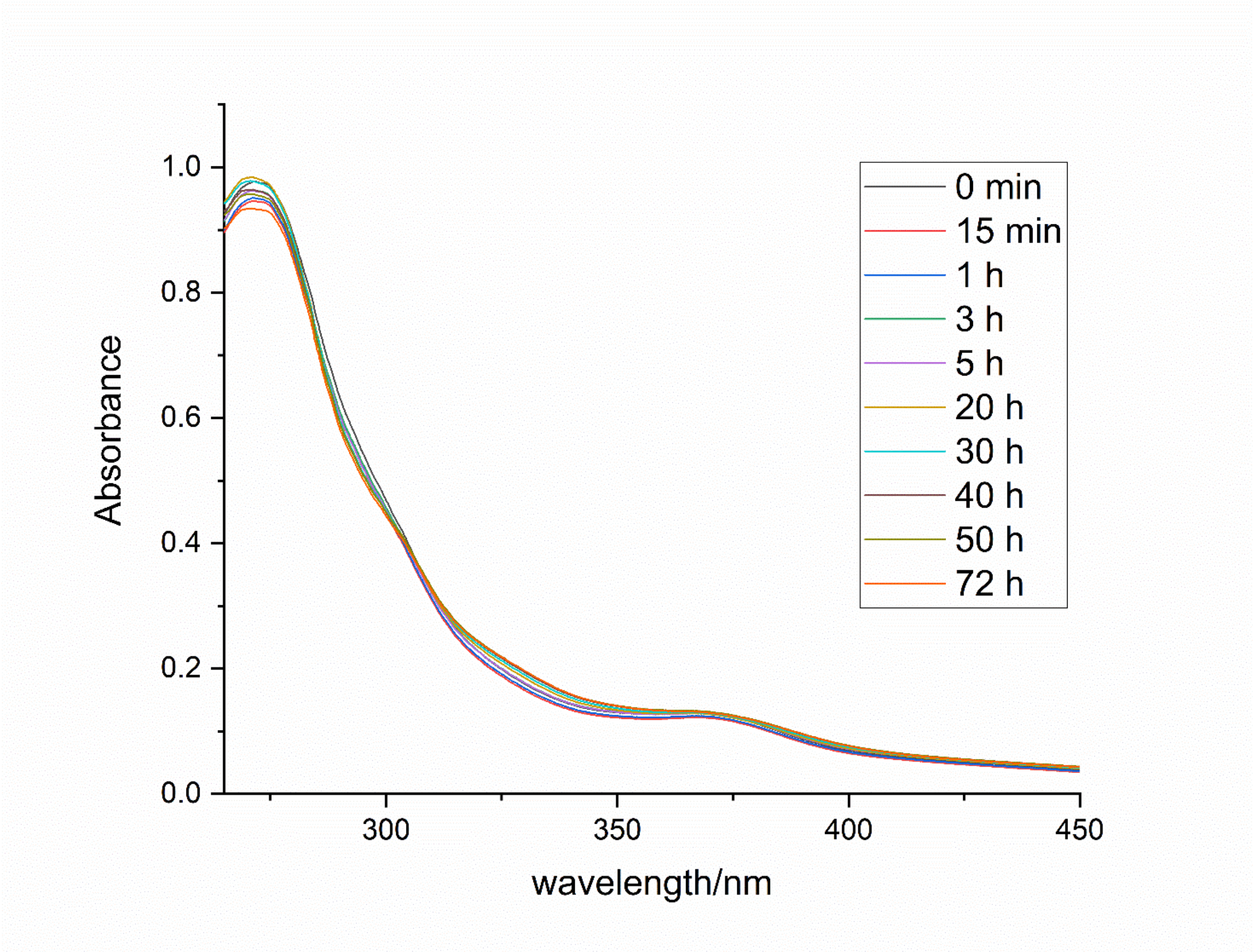

Figure S45. Selection of electronic spectra of the complex $\mathbf{3}^{S}$ in saline solution (0.9% w/v) acquired during a 72-h scanning kinetics.

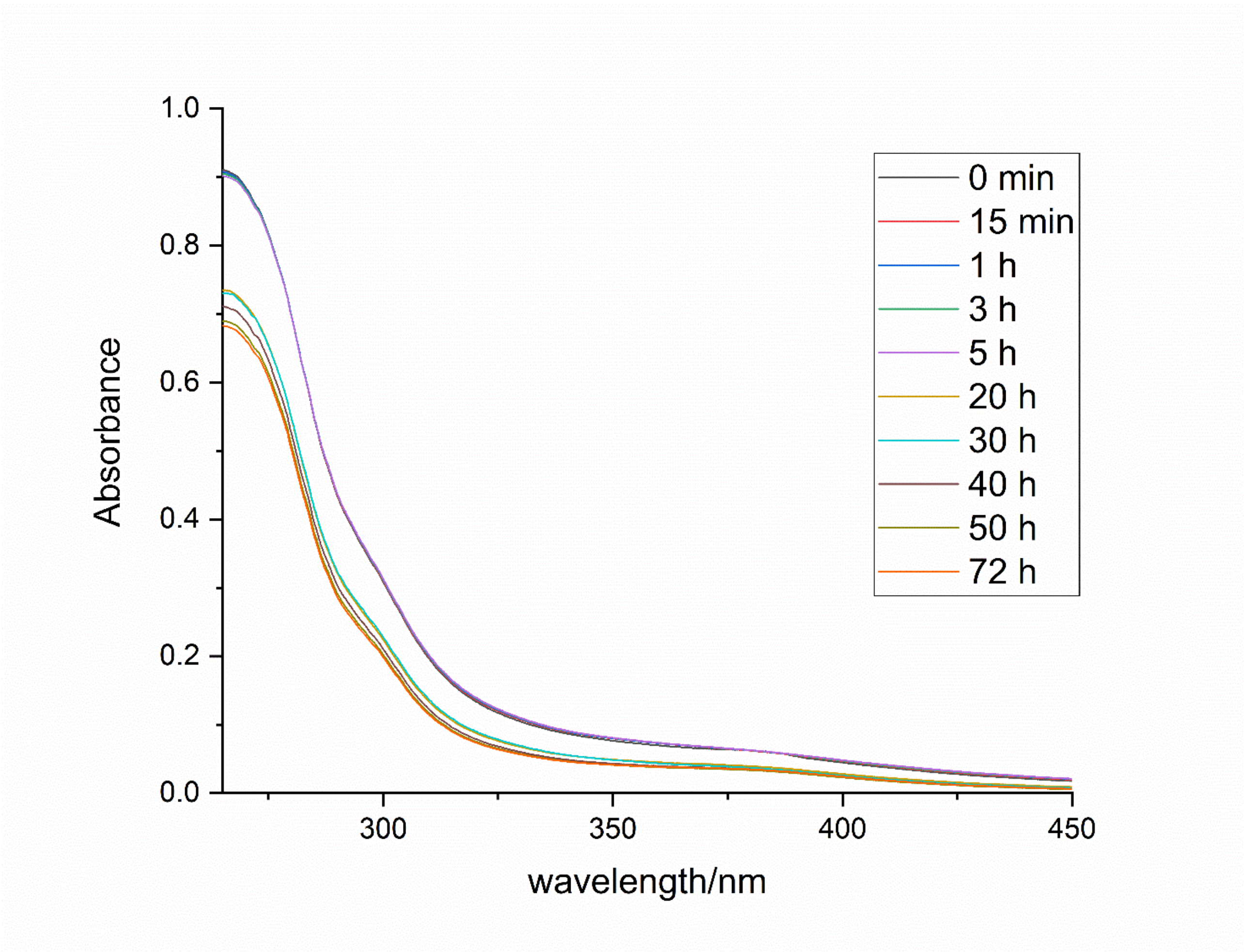

Figure S46. Selection of electronic spectra of the complex **4**^{S} in saline solution (0.9% w/v) acquired during a 72-h scanning kinetics.

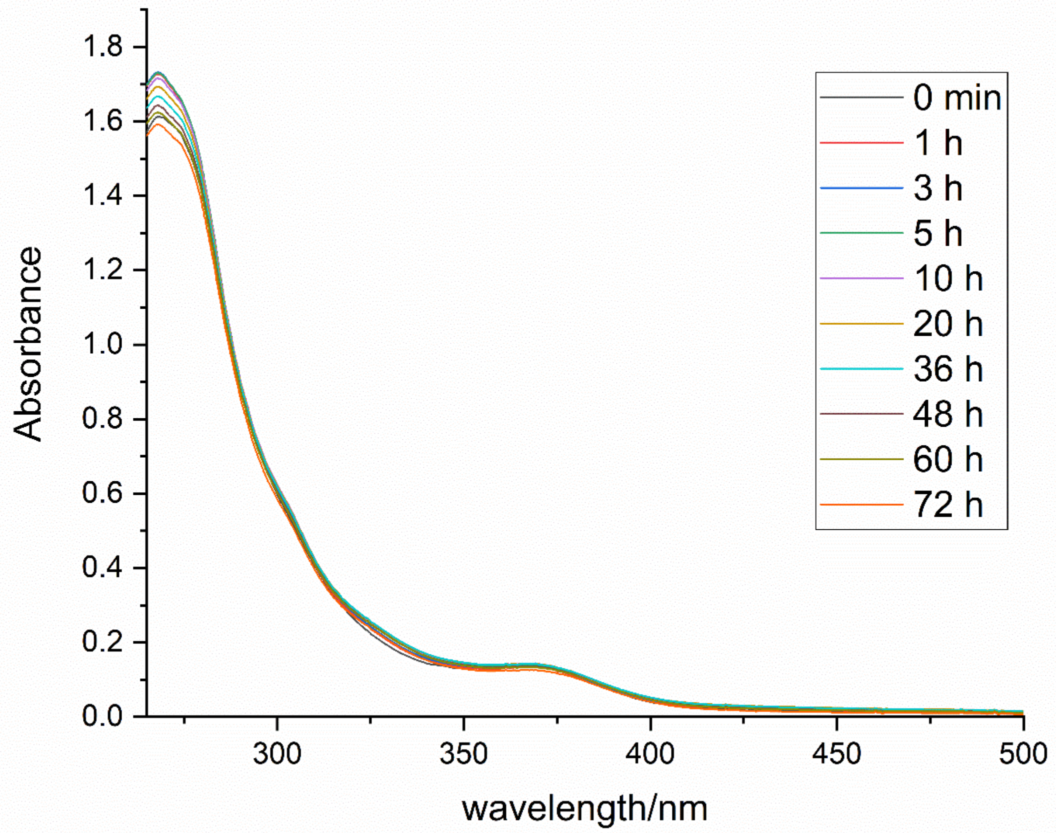

Figure S47. Selection of electronic spectra of the complex $\mathbf{2}^{S}$ in PBS acquired during a 72-h scanning kinetics.

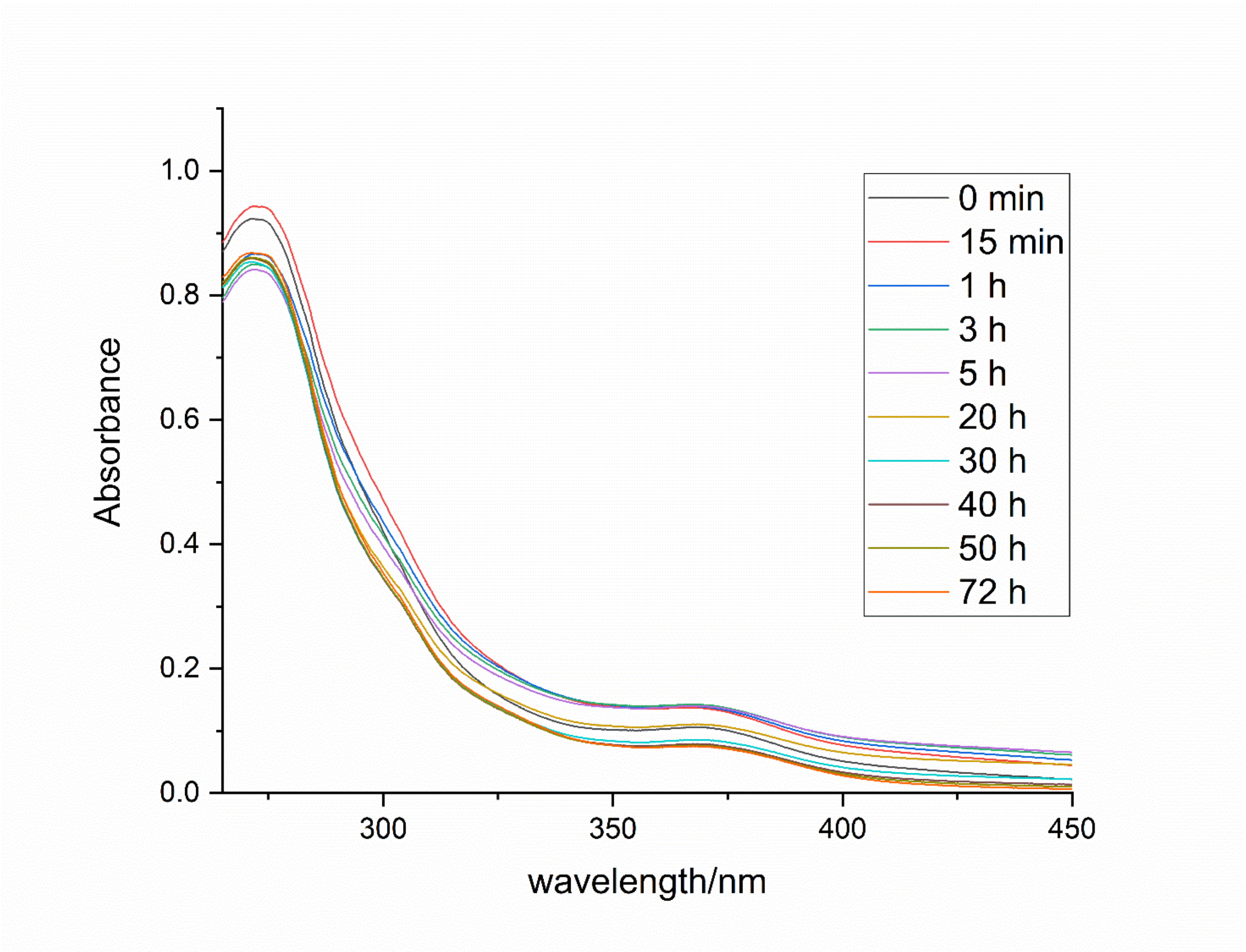

Figure S48. Selection of electronic spectra of the complex $\mathbf{3}^{S}$ in PBS acquired during a 72-h scanning kinetics.

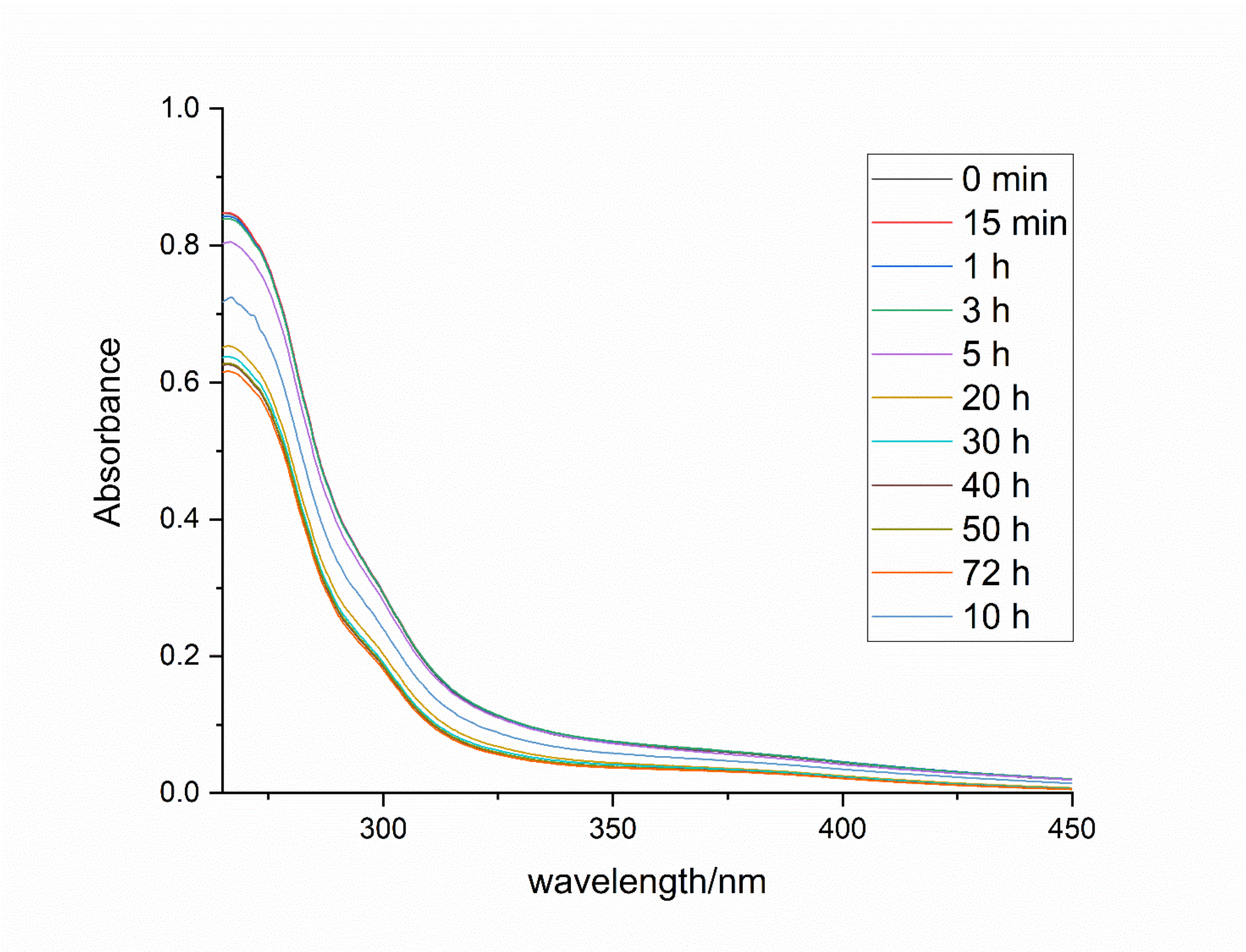

Figure S49. Selection of electronic spectra of the complex **4**^{S} in PBS acquired during a 72-h scanning kinetics.

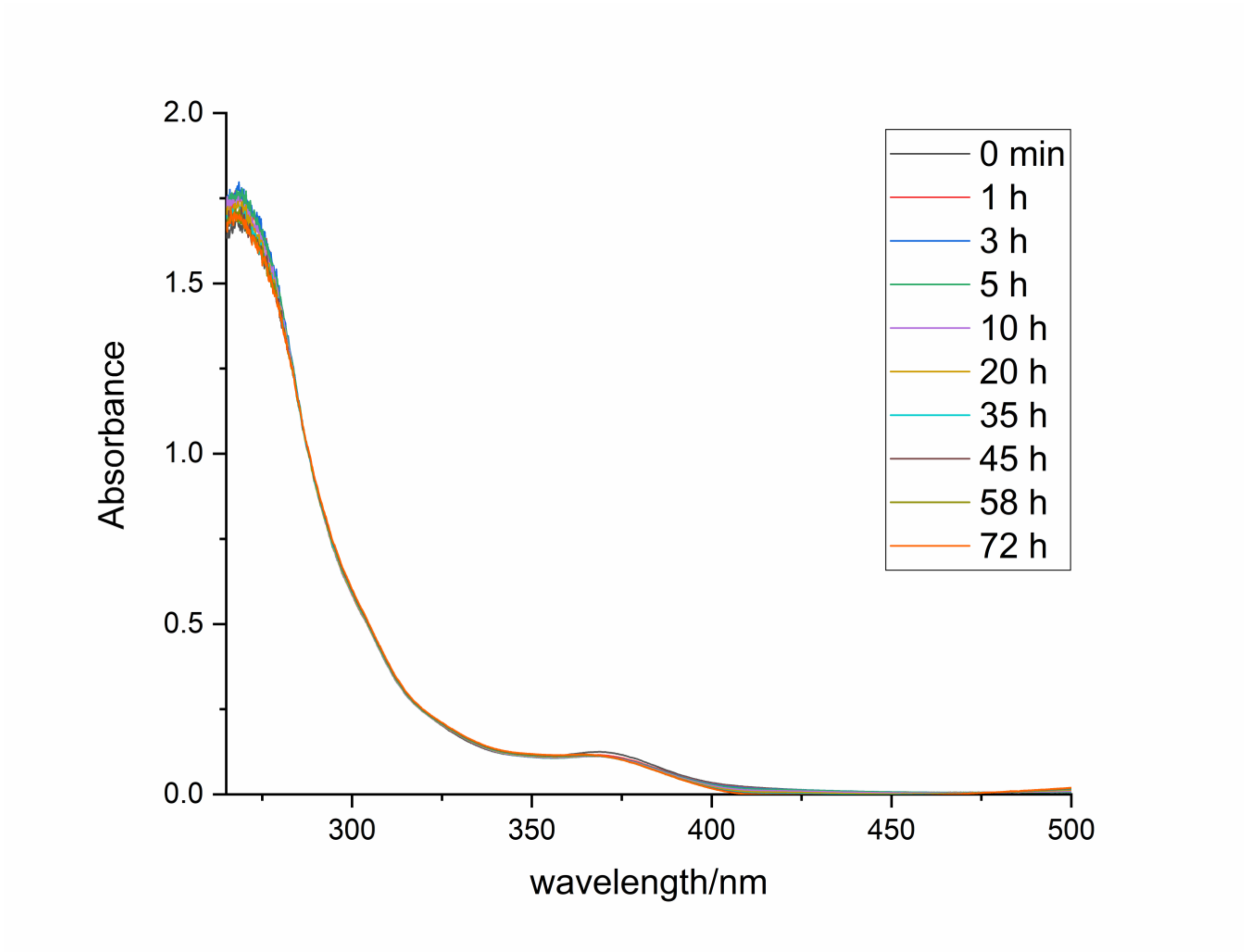

Figure S50. Selection of electronic spectra of the complex **2**^{S} in DMEM (25% v/v) acquired during a 72-h scanning kinetics.

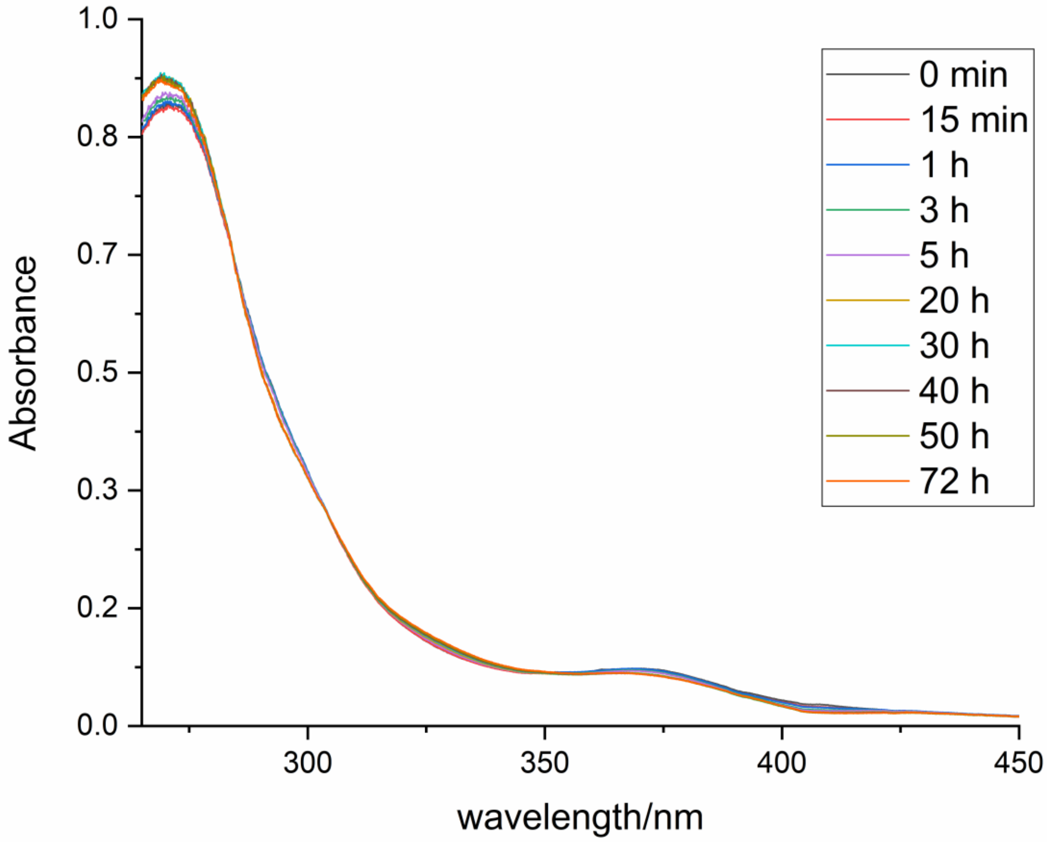

Figure S51. Selection of electronic spectra of the complex $\mathbf{3}^{S}$ in DMEM (25% v/v) acquired during a 72-h scanning kinetics.

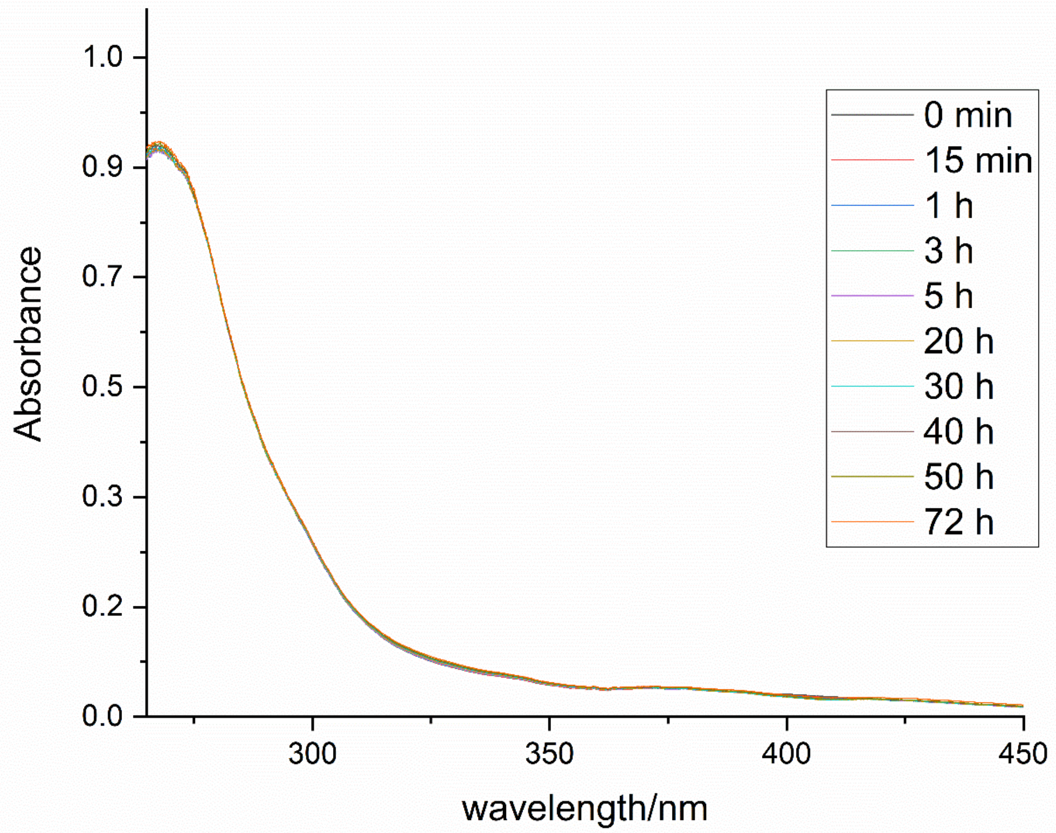

Figure S52. Selection of electronic spectra of the complex $\mathbf{4}^{S}$ in DMEM (25% v/v) acquired during a 72-h scanning kinetics.

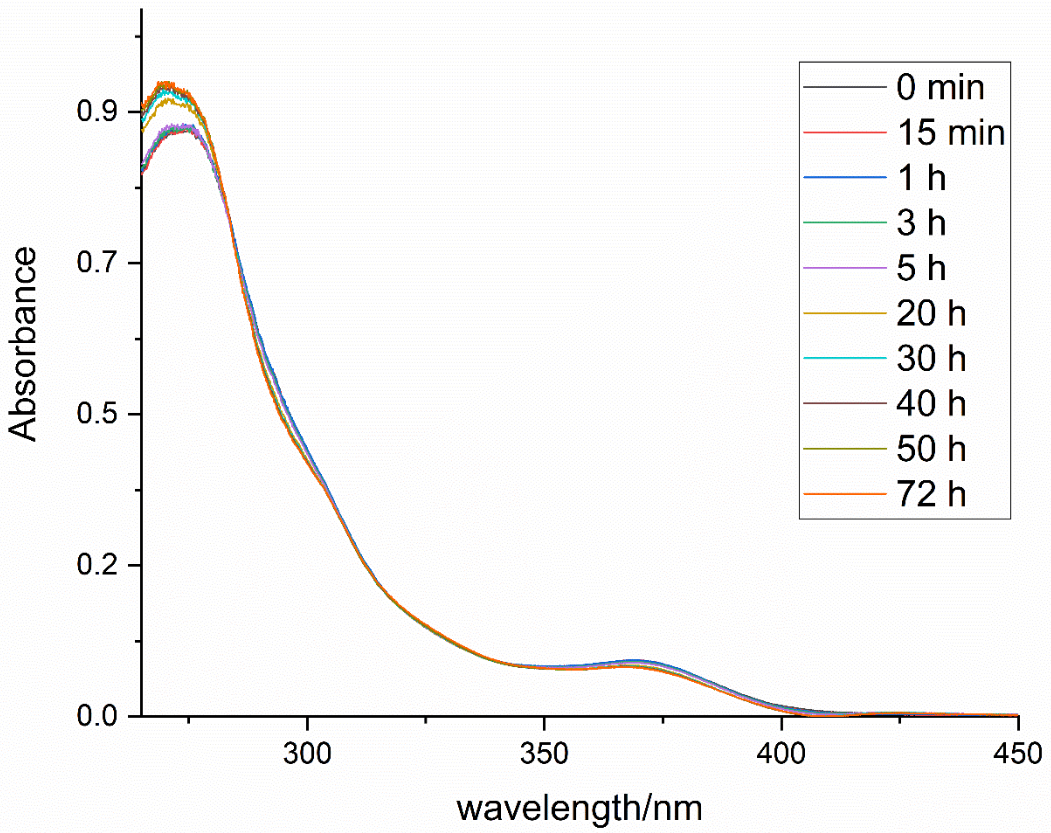

Figure S53. Selection of electronic spectra of the complex $\mathbf{3}^{R}$ in DMEM (25% v/v) acquired during a 72-h scanning kinetics.

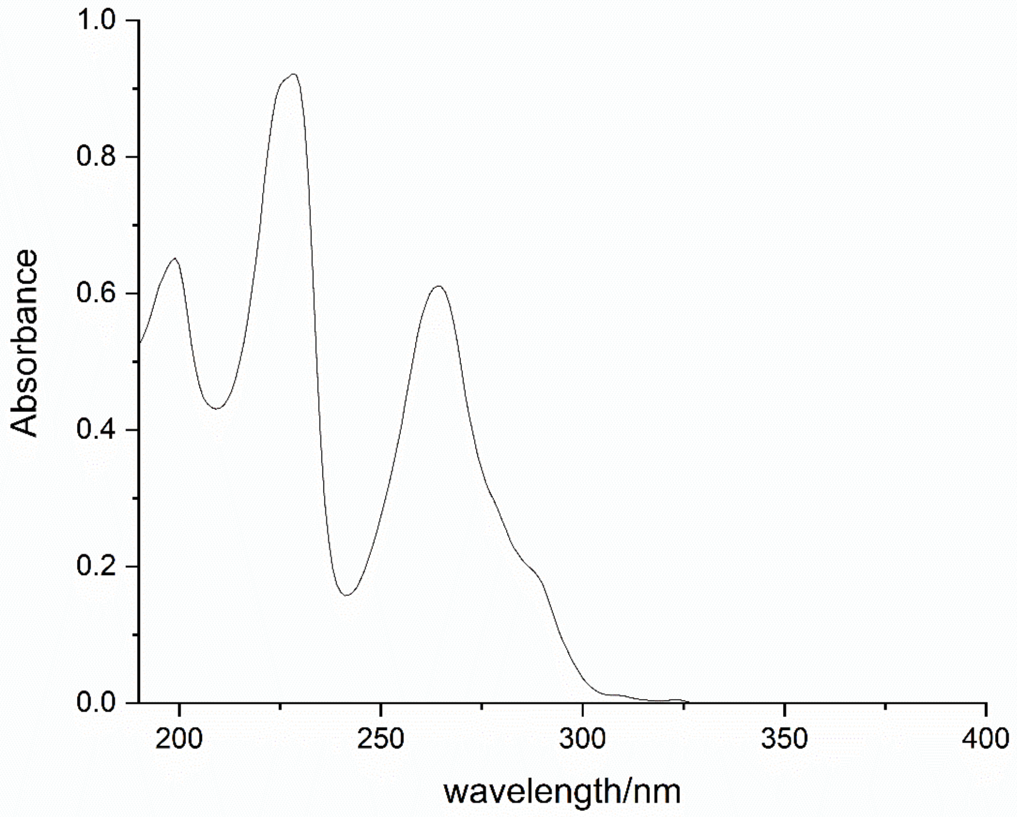

Figure S54. Electronic spectrum of 1,10-phenanthroline ligand in water.

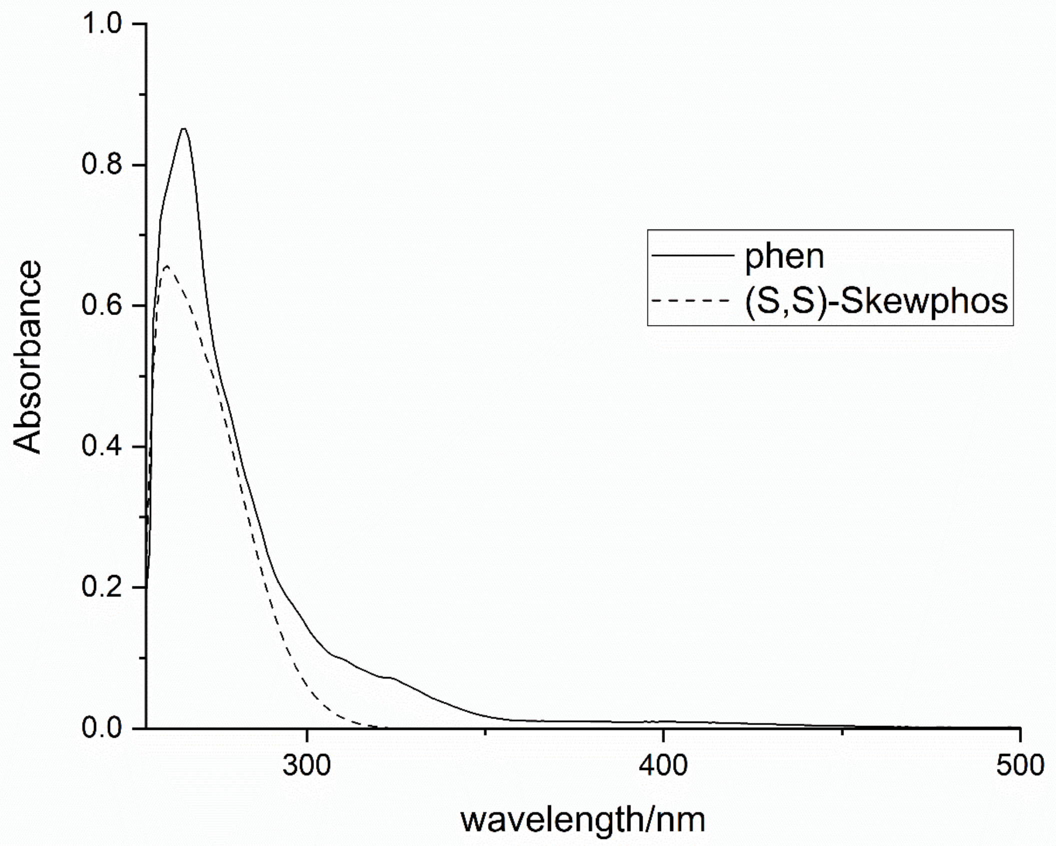

Figure S55. Overlapped electronic spectra of 1,10-phenanthroline and (S,S)-Skewphos in DMSO.

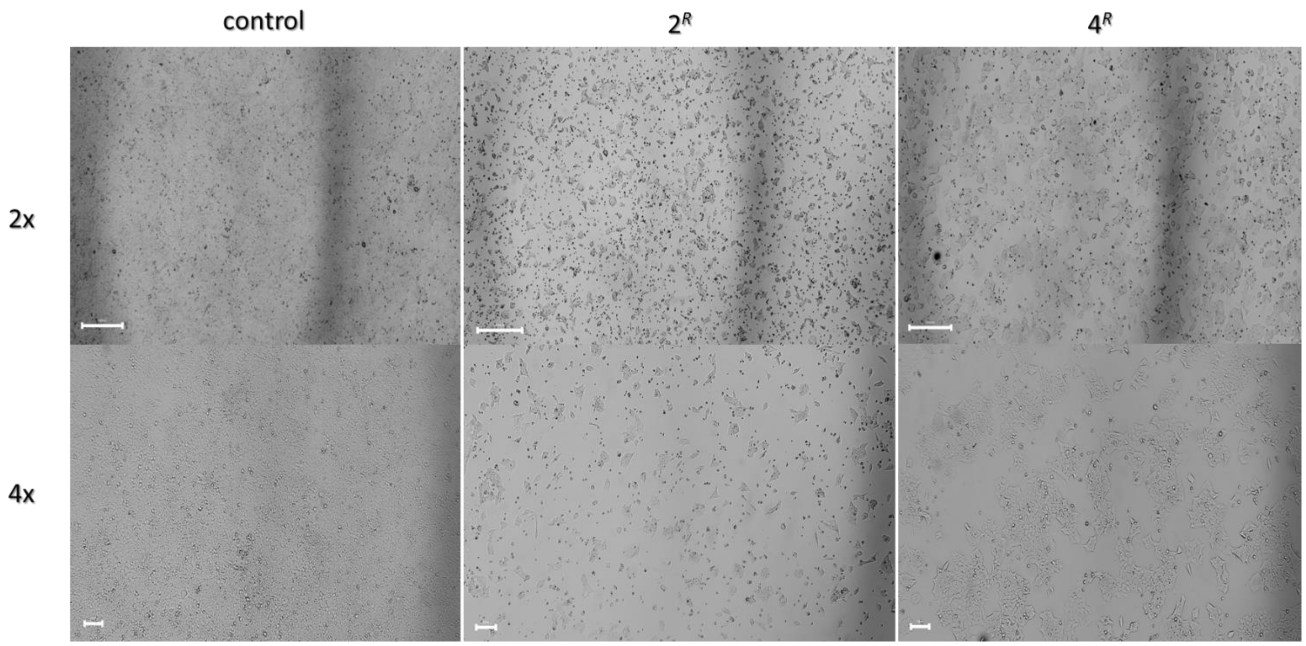

Figure S56. Images collected from Olympus IX70 inverted tissue culture microscope for the evaluation of cellular morphology changes after treatment with DMSO (control), $\mathbf{2^{R}}$ and $\mathbf{4^{R}}$.

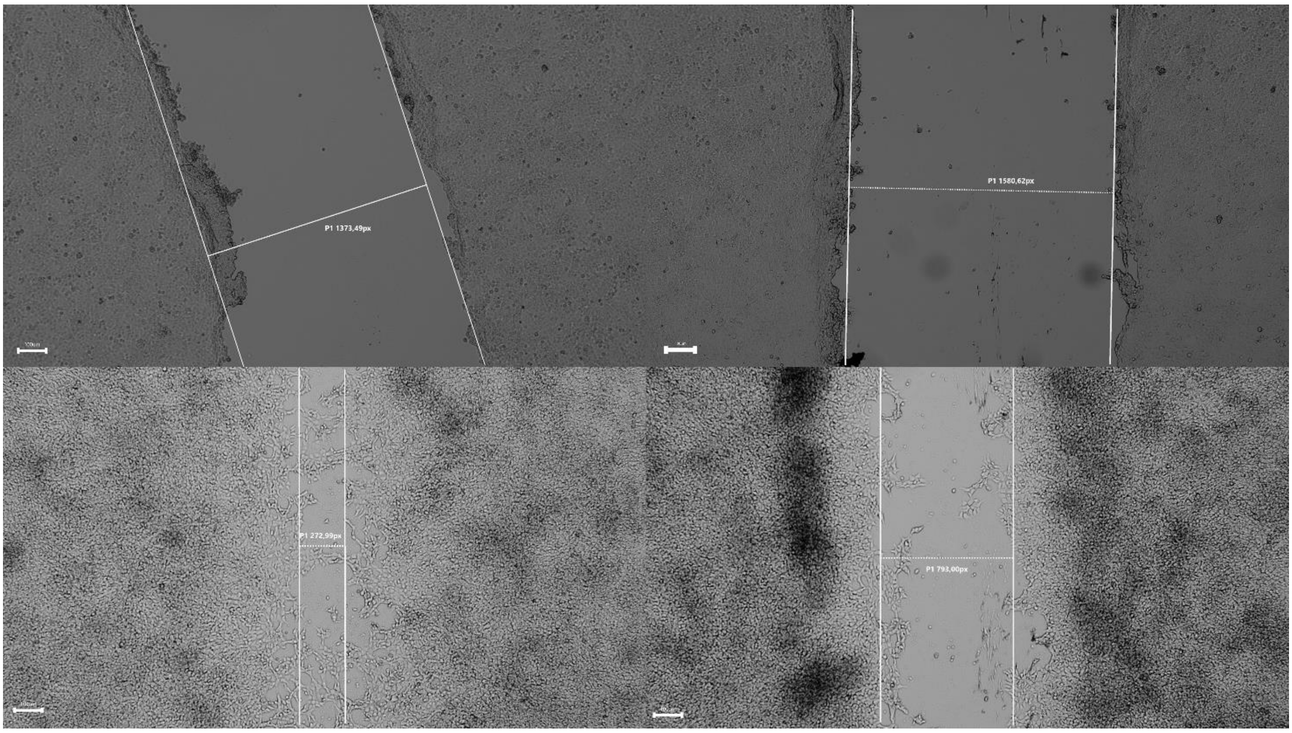

Figure S57. Images collected from Olympus IX70 inverted microscope when studying cell migration without (Control, left) or with complex $\mathbf{2^{Rv}}$ (right). Pictures here reported are representative of one of three different experiments (original magnification 4X; scale bar = 100 µm). Upper panel: day 0; lower panel: day 5.
